# A randomized parallel-designed study evaluating the effect of an app-based nutritional concept for weight reduction and further cardiovascular risk factors: Insights from the PreVA study

**DOI:** 10.3389/fnut.2026.1815820

**Published:** 2026-09-23

**Authors:** Lea Klein, Maren Biernath, Kristin Kipp, Michael Kiehntopf, Susann Scherag, Katharina Meier, Thomas Hübner, Stefan Lorkowski, Christine Dawczynski

**Affiliations:** 1 Junior Research Group Nutritional Concepts, Institute of Nutritional Sciences, Friedrich Schiller University Jena, Jena, Germany; 2 Department of Pediatrics Weimar, Sophien- und Hufeland-Klinikum, Weimar, Germany; 3 Institute of Clinical Chemistry and Laboratory Diagnostics, University Hospital, Jena, Germany; 4 Preventicus GmbH, Jena, Germany; 5 Competence Cluster for Nutrition and Cardiovascular Health (nutriCARD) Halle-Jena-Leipzig, Jena, Germany; 6 Institute of Nutritional Sciences, Friedrich Schiller University Jena, Jena, Germany

**Keywords:** App, cardiovascular risk factors, energy intake, nutritional concept, weight reduction

## Abstract

**Introduction:**

Overweight is a major public health concern. Beyond traditional dietary therapy, mobile health (mHealth) applications have gained interest for their potential to support weight loss. However, evidence on their efficacy remains limited. This study evaluated the effectiveness of an app-based dietary concept for weight reduction and improvement of further cardiovascular risk factors. Methods: The PreVa study is a single-center, randomized, four-arm parallel intervention study comparing an app-based nutrition program with standard therapy involving personal counseling. Eighty adults with a body mass index between 25 and < 35 kg/m^2^ were randomly assigned to one of four groups (1) app-based, (2) individual coaching, (3) general dietary recommendations and (4) control. All intervention groups received individualized, nutrient-optimized menu plans with integrated flaxseed oil supplementation, either via app or in printed form, combined with varying levels of counseling. Energy intake was gradually reduced every 4 weeks, with maintenance phases at the beginning and end of the 16-week intervention. Anthropometric and biochemical parameters were measured at four-week intervals.

**Results:**

After 12 weeks of energy reduction, weight loss was significantly greater in the intervention groups (−6.5 %, −7.3 %, −6.0 %) than in the control group (−2.1 %; *p* < 0.001) and remained stable during the maintenance phase (16 weeks; all *p* < 0.01). A clinically relevant (> 5 %) weight loss was achieved by 66.7 %, 78.9 %, and 53.3 % of participants for all interventions, respectively. Blood pressure was reduced in all intervention groups (*p* < 0.05), while the values differ not significantly from control group. Total and LDL cholesterol decreased in group 2 and 3 (*p* < 0.05), the changes differ significantly from the control group. Group 1 exhibited a significant decrease in fasting glucose and HOMA-IR compared to the other groups. α-Linolenic concentrations increased markedly (+ 52.7–79.4 %; *p* ≤ 0.01), whereas eicosapentaenoic acid and docosahexaenoic acid remained unchanged. No relevant changes were observed in micronutrient status.

**Conclusion:**

The intervention groups achieved clinically meaningful weight loss and an improvement in blood lipid levels. The greatest effect is likely attributable to the individualized, nutrient-optimized menu plans rather than the type of counseling or delivery mode.

**Clinical trial registration:**

https://clinicaltrials.gov/, identifier NCT04717076

## Introduction

The prevalence of overweight (body mass index (BMI) ≥ 25 to < 30 kg/m^2^) and obesity (BMI ≥ 30 kg/m^2^) is continuously increasing, making it a global public health concern. In 2021 and 2022, 43 % of adults aged 18 years and over were overweight and 12–16 % were living with obesity (1, 2). Since 1990, the number of adults has doubled and even quadrupled for adolescents (3). Overweight (including obesity) is a major risk factor for numerous comorbidities such as cardiovascular disease (CVD), type 2 diabetes mellitus (T2DM), respiratory diseases, various types of cancer and depression (4, 5). Beyond the substantial impact on individual well-being, this trend also imposes challenges for the healthcare system due to rising costs of the treatment of comorbidities (5).

The primary contributors to obesity are increasing physical inactivity and hypercaloric diets characterized by unhealthy eating patterns (6). A recent systematic analysis of the Global Burden of Disease Study revealed that 36.7 % of CVD deaths were attributable to an unbalanced diet (7). Especially in western countries, diets contain too many calories, salt, saturated fatty acids (SFA) and refined sugars, while the consumption of monounsaturated fatty acids (MUFA), polyunsaturated fatty acids (PUFA; especially *n*-3 PUFA) and fiber is too low.

According to the German Obesity Society, the basic principles of weight management are a combination of dietary changes, especially energy restriction in consideration of an adequate supply of micronutrients, increased exercise and behavioral modification. In addition to traditional nutritional therapy, there has been growing interest in the field of mobile health (mHealth) to develop app-based concepts with the potential to facilitate weight loss (8–11). These applications offer the advantage to remove barriers related to accessibility and availability of treatment centers and therefore reach more people in priority groups (12). As those applications can be used anytime and anywhere, this may also enhance compliance with dietary and lifestyle interventions. Although an abundance of mHealth apps, especially lifestyle apps, can be found, the data on their effectiveness remains heterogenous. This is partly due to the variability in app features and the limited availability of approved evidence-based applications (10).

Therefore, the present study developed a nutritional concept aimed at achieving weight loss while ensuring an optimized nutrient supply. The concept is based on a gradual calorie reduction and provides nutrient-optimized daily menu plans using a mHealth app for smartphones. The effectiveness of this intervention was compared to standard nutritional therapy, with or without daily menu plans. The primary objective was to achieve a clinically relevant weight reduction (5–10 % of initial body weight) by modulating dietary behavior and eating habits. In addition, the improvement of other cardiovascular risk factors and nutrient status were considered as secondary outcomes.

## Materials and methods

### Study design and dietary intervention

The PreVa study is a single-center, randomized, four-arm intervention trial in parallel design, which was conducted in Jena, Germany between April and Juli 2021. This controlled proof-of-concept study examines the influence of an individualized nutritional concept based on a gradual reduction in energy intake with an optimized nutrient profile on weight loss and further cardiovascular risk factors. In addition, the effectiveness of different communication methods [app-based vs (personal) nutritional counselling] was tested in a parallel design (allocation ratio: 1:1:1:1). Due to the nature of the intervention, blinding of participants and study personnel was not feasible. The primary objective was weight reduction (5–10 % of initial weight), while secondary endpoints included body composition, blood pressure, blood lipids, diabetes risk factors, erythrocyte fatty acid composition, and nutrient status assessed in blood and urine samples.

The study consisted of three phases: a seven-day run-in phase, during which participants were required to complete a dietary log, an intervention phase and a stabilization phase. Before starting the run-in phase, participants were randomly assigned to one of four groups (*n* = 20 per group; Figure 1):

**FIGURE 1:**
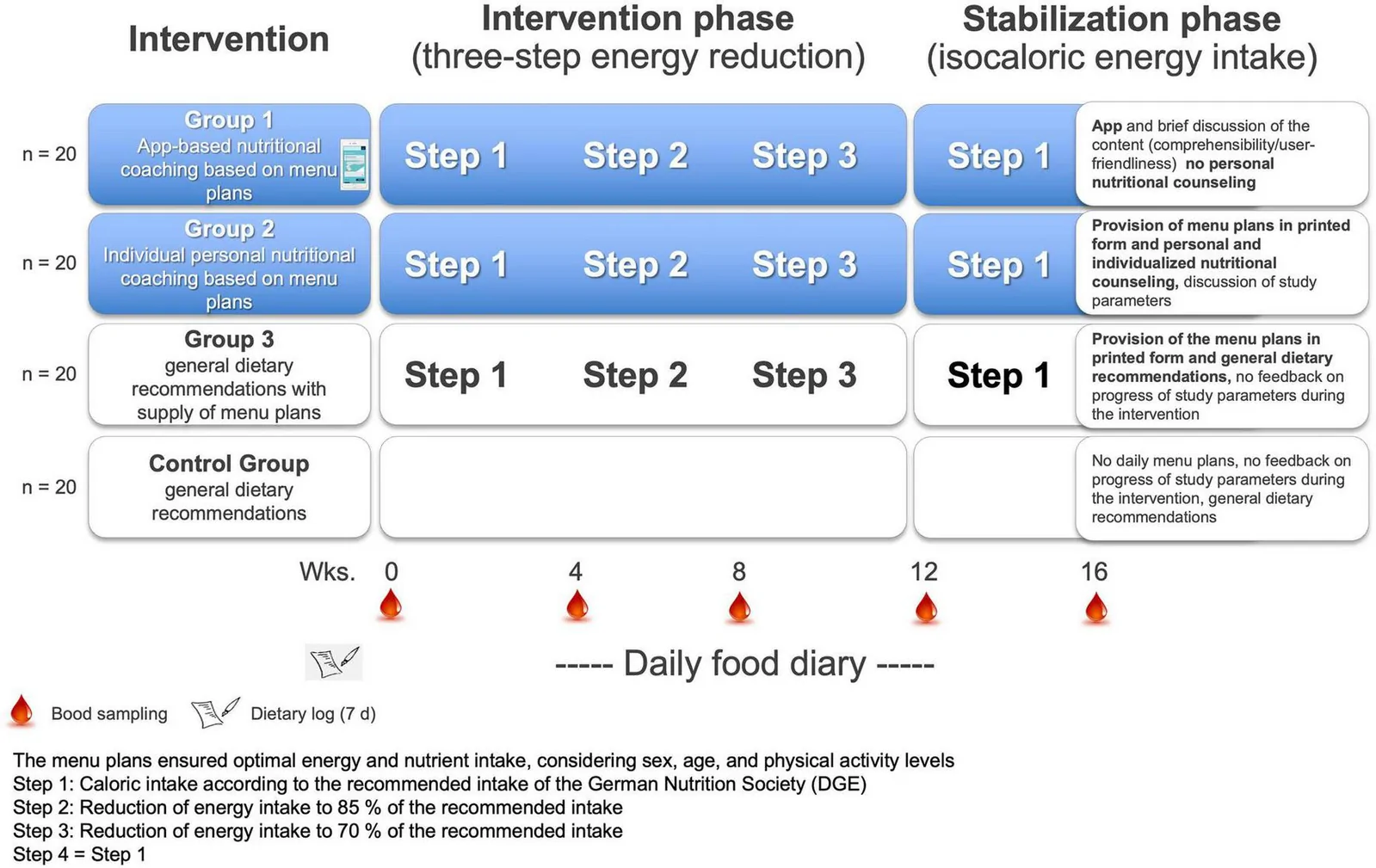
Study design of the PreVa study.

Group 1: App-based nutritional coaching (based on menu plans)

- Energy- and nutrient-optimized daily menu plans were delivered via a dedicated study app (Preventicus, Jena, Germany). The plans were updated regularly throughout the intervention
- Health information, nutritional information, and visualization of the cardiovascular risk profile was provided via app
- A shopping list could be created in the app to facilitate implementation of the menu plans

Group 2: Individual personal nutritional coaching (based on menu plans)

- Energy- and nutrient-optimized daily menu plans were provided in printed form
- Individual nutritional counseling sessions, inclusive feedback on the progress of study parameters

Group 3: Supply of menu plans (no further information, guidance)

- Energy- and nutrient-optimized daily menu plans were provided in printed form
- No feedback on progress of study parameters

Group 4: Control group

- No daily menu or recipe plans were provided
- General dietary recommendations based on the recommendations of the German Nutrition Society (Deutsche Gesellschaft für Ernährung, DGE)

Participants were immediately assigned to one of the three intervention and control group by permuted block randomization with block size 8 and an allocation ratio of 1:1:1:1. The random allocation sequence was generated by the study leader using statistical software R version 3.5.1 (The R Foundation for Statistical Computing, Vienna, Austria). Recruitment was carried out by the study team via social media accounts, press releases and leaflets. Participants included in the study were assigned a sequential number.

Recruitment was carried out by the study team via social media accounts, press releases and leaflets. Participants who met the inclusion criteria and were eligible for inclusion were assigned a sequential number. In accordance with the randomization list drawn up in advance, participants were allocated to one of the four groups on the basis of their sequential number.

The intervention phase was originally planned for 24 weeks with study visits every 6 weeks. However, due to the challenges of the COVID-19 pandemic, it was only possible to implement a duration of 16 weeks, with scheduled visits occurring every 4 weeks. At the study visits, blood samples and anthropometric data were taken, and the respective counseling session took place. The dietary intervention was structured as a three-step caloric reduction with the final phase serving as a stabilization phase (Figure 1). The menu plans were generated using PRODI software version 6.8 (Nutri-Science, Stuttgart, Germany) and were available in 13 distinct energy levels, customized to align with the individual energy and nutrient requirements, based on sex, age, and physical activity levels (PAL). Two PAL levels were considered: 1.4 (predominantly sedentary with little leisure activity) and 1.6 (predominantly sedentary with additional standing activities, e.g., drivers or students). In the initial phase of the intervention, the energy intake was set at a level corresponding to the recommended intake of the DGE. In the second and third phases, the energy intake was reduced to 85 % and 70 % of the recommended amount, respectively (Figure 1). The fourth phase was identical to the first (stabilization phase). The plans ensured an optimal nutrient intake and adhered to the following criteria:

- Macronutrient composition according to the DGE reference values: 40–55 total energy (En %) carbohydrates, 15 En % protein, 30–35 En % fat
- Optimized fatty acid intake: SFA: ≤ 7 En %, MUFA: ≥ 10 En %, PUFA: ≥ 10 En %, including an α-linolenic acid (ALA) intake of approximately 3 g/d and at least 500 mg/d of long-chain (LC) omega (*n*)-3 fatty acids
- Reduced cholesterol intake ≤ 200 mg/d
- Increased dietary fiber intake: minimum: 30 g for energy levels of 1300–1600 kcal; maximum: 80 g for energy levels of 2700–2800 kcal

Detailed instructions on how to draw up the used meal plans, including examples, have already been published (13, 14).

### Participants

The study investigates 80 participants (men and women) aged 25 to 51 years with a BMI ranging from ≥ 25 to < 35 kg/m^2^ corresponding to overweight or obesity class 1. Participants needed to demonstrate the willingness and ability to adhere to the study protocol and should own a smartphone. Only individuals with no or moderate alcohol consumption (≤ 2 glasses per week) were considered. Preferably, non-smokers were included. Participants were excluded if they presented with any of the following diagnosed conditions: hypercholesterolemia, diabetes mellitus or thyroid dysfunction (hyperthyroidism or hypothyroidism). In addition, individuals with known food intolerances or allergies were excluded, as were those taking lipid-lowering medications, glucocorticoids, or dietary supplements, particularly *n*-3 fatty acids. Further exclusion criteria were daily intense physical exercise, alcohol, drug and medication abuse as well as participation in other clinical observational studies during or within 4 weeks prior to the beginning. Individuals with severe behavioral, emotional, or psychiatric issues, which could hinder compliance in the investigator’s judgment, were not eligible. Pregnancy, breastfeeding, or unreliable contraception also led to exclusion.

Prior to the run-in, 125 subjects were recruited and assessed for eligibility; 45 were excluded as they did not meet the inclusion criteria or declined to participate. 80 subjects were randomly assigned to one of the four study groups. After completion of the run-in phase (*n* = 80), the intervention phases followed. 11 subjects did not finish the intervention due to personal reasons and were therefore not included in the statistical analysis (Figure 2).

**FIGURE 2:**
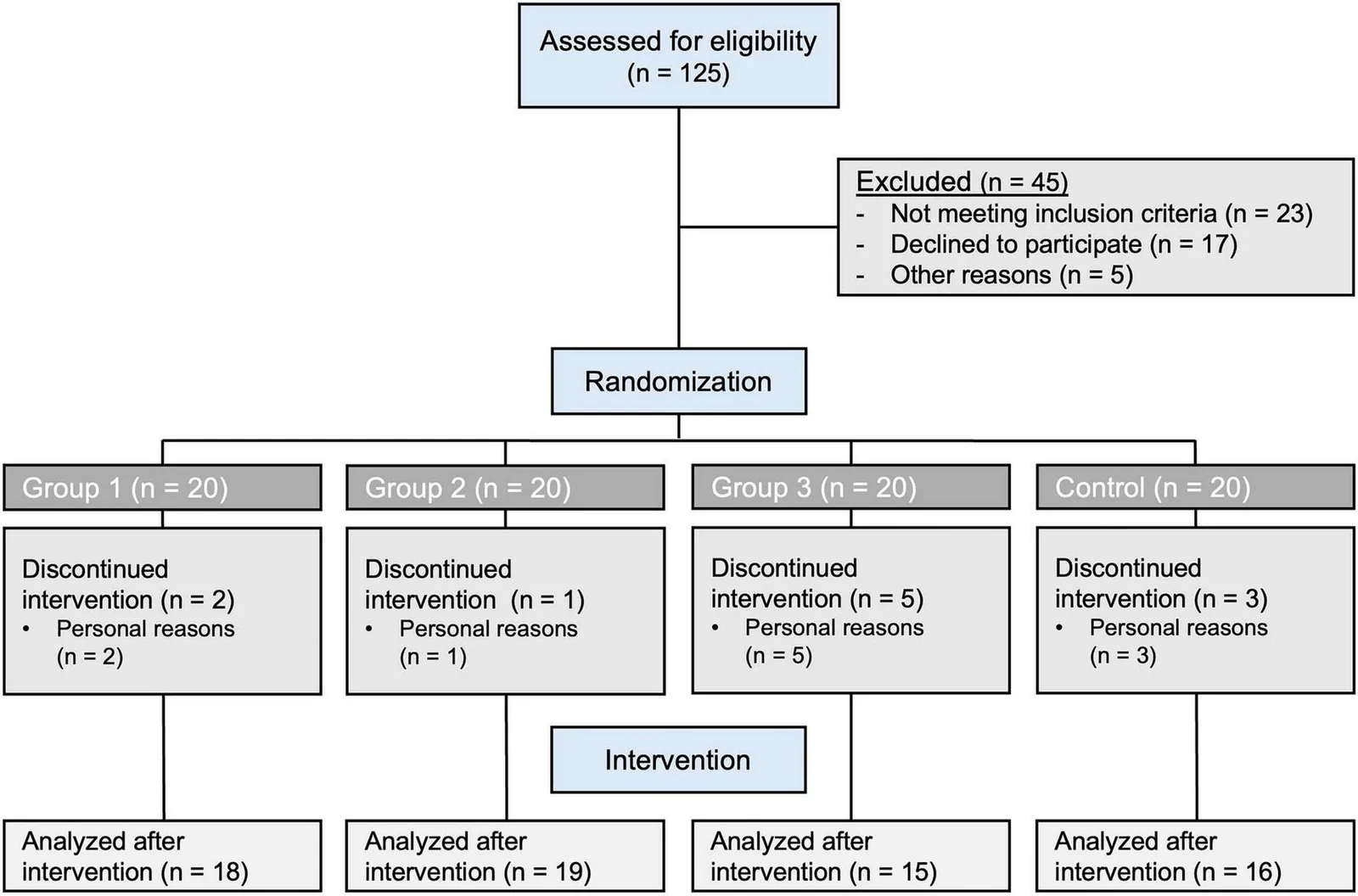
Flowchart diagram of the PreVa study.

### Assessment of nutritional habits

To record and document the intake of food and beverages a full self-reporting of individual dietary intake over 5 days was conducted by the participants. The dietary record is based on the template “Freiburger Ernährungsprotokoll” which was provided by PRODI^®^ version 6.8 (Nutri-Science, Stuttgart, Germany) and includes common foods and usual portion sizes. Foods that were not listed in the template were manually documented by the subjects including the name and the amount of the food consumed. The daily energy and nutrient intake was calculated using PRODI^®^.

Throughout the study period, satisfaction with the meal plans and the feasibility of following the dietary plans in everyday life were recorded and assessed on a daily basis. To this end, participants rated each meal on a four-point scale (1 = excellent and tasty, 2 = good, 3 = average, 4 = I would not prepare or eat this again). In addition, any deviations from the prescribed meals were to be briefly noted. A total of 50 participants from groups 1 to 3 kept the food diary. Meals were predominantly rated as 1 or 2; ratings in categories 3 or 4 were rare. The number of documented deviations from the dietary plan was predominantly between 0 and 2 per day; in isolated cases, 3 to 4 deviations per day were recorded. Reasons given for deviations included missing ingredients, lack of time, birthdays, leisure activities and shift work. In some cases, foods that were considered too rich were also omitted. Overall, these data suggest that the participants largely adhered to the dietary guidelines provided to them in the form of individualized meal plans.

### Sample collection, parameter analyses and further measurements

Blood samples were collected via venipuncture after an overnight fasting period of at least 12 h. Participants were instructed to refrain from excessive physical activity and alcohol consumption on the day preceding and the morning of the venipuncture. Urine samples were collected over a 24-h period prior to the baseline assessment and again prior to the end of the stabilization phase (week 16). Fasting peripheral venous blood samples were centrifuged to separate erythrocytes, plasma, and serum. The study parameters were either analyzed immediately after blood sampling or urine collection or were stored at −80 °C using aliquots (erythrocytes, serum, plasma) or at −20 °C (24-h urine) until the analysis. All samples were prepared according to standard operation procedures. All study parameters (with the exception of fatty acid distribution in erythrocytes) were analyzed in serum, plasma, and urine at the Institute of Clinical Chemistry and Laboratory Diagnostics, Jena University Hospital, using the Abbott Architect CI 16200 analyzer (Abbott, Wiesbaden, Germany). The analysis of fatty acid distribution in erythrocytes was conducted by the Institute of Nutritional Sciences, Friedrich Schiller University Jena. At first, fat was extracted using the Folch and Bligh and Dyer procedures (15, 16). Afterward, the extracted lipids were saponified and methylated with BF_3_ and the success of the methylation process was confirmed by separation on silica gel aluminum plates. The resulting fatty acid methyl esters (FAME) were then analyzed via gas chromatography (GC-17V3, Shimadzu, Duisburg, Germany). The quantification of each FAME was calculated using LabSolutions software version 5.92 (Shimadzu). The data are presented as a percentage of the total FAME content (%FAME). For calculations of the sum of *n*-3 PUFA, the following *n*-3 PUFA were included: α-linolenic acid (ALA, C18:3*n*-3), eicosatetraenoic acid (C20:4*n*-3), eicosapentaenoic acid (EPA, C20:5*n*-3, docosapentaenoic acid (DPA, C22:5*n*-3 and docosahexaenoic acid (DHA, C22:6*n*-3). The sum of *n*-6 PUFA comprises the following fatty acids: linoleic acid (LA, C18:2*n*-6), γ-linolenic acid (GLA, C18:3*n*-6), C20:2*n*-6, dihomo γ-linolenic acid (C20:3*n*-6), arachidonic acid (ARA, C20:4*n*-6), adrenic acid (C22:4*n*-6) and osbond acid (C22:5*n*-6). The *n*-3 index was calculated by using the sum of EPA and DHA in erythrocyte lipids. The *n*-3 LCPUFA correspond to the sum of EPA, DPA and DHA. Phenotypic markers of desaturase activity were used as surrogate measures of tissue desaturase activities based on the ratios of relevant product and precursor fatty acids, as follows: C18:1*n*-9/18:0 (Δ9-desaturases, D9D), C20:4*n-*6/C20:3*n-*6 (Δ5-desaturase, *n*-6 D5D), C20:5*n-*3/C20:4*n-*3 (*n*-3 D5D), C20:3*n-*6/C20:2*n-*6 (Δ6-desaturase, *n*-6 D6D), and C20:5*n-*3/C18:3*n*-3 (*n*-3 D6D).

Further, %*n-*6 and %*n*-3 in highly unsaturated fatty acids (HUFA), which comprise fatty acids with three or more double bonds in their carbon chains, were calculated according to formula (1) and (2), respectively.


$$\left(1\right)\%\,\text{n}-6\,\mathrm{in}\,\mathrm{HUFA}:100\times \frac{\,\mathrm{C20}:3\,\text{n}-6+\text{ARA}+\mathrm{C22}:4\,\text{n}-6+\mathrm{C22}:5\,\text{n}-6}{\,\mathrm{C20}:3\,\text{n}-6+\text{ARA}+\mathrm{C22}:4\,\text{n}-6+\mathrm{C22}:5\,\text{n}-6+\mathrm{C20}:3\,\text{n}-3+\mathrm{C20}:4\,\text{n}-3+\text{EPA}+\text{DPA}+\text{DHA}}$$

$$\left(2\right)\%\,\text{n}-3\,\mathrm{in}\,\mathrm{HUFA}:100\times \frac{\,\mathrm{C20}:3\,\text{n}-3+\mathrm{C20}:4\,\text{n}-3+\text{EPA}+\text{DPA}+\text{DHA}}{\,\mathrm{C20}:3\,\text{n}-6+\text{ARA}+\mathrm{C22}:4\,\text{n}-6+\mathrm{C22}:5\,\text{n}-6+\mathrm{C20}:3\,\text{n}-3+\mathrm{C20}:4\,\text{n}-3+\text{EPA}+\text{DPA}+\text{DHA}}$$

Anthropometric, body composition and blood pressure measurements were consistently obtained by a trained study nurse as single measurements using calibrated instruments. The circumference of the waist was measured with an ergonomic tape measure (Seca 212, Seca, Hamburg, Germany) at the midpoint between the inferior margin of the ribs and the iliac crest. Body height was measured using a scale with an integrated stadiometer (Seca 813, Seca, Hamburg, Germany). Arterial blood pressure was measured in a seated position on the upper arm with an automatic blood pressure device (Boso-medicus uno, Bosch + Sohn, Jungingen, Germany), following a minimum 10-min period of rest in this posture. Body composition was evaluated using a body impedance analyzer (seca 515/514, seca, Hamburg, Germany).

### Statistical methods

The power analysis was based on the study results of Lam et al. (17) and was conducted using the statistical software G*Power version 3.1.9.7 (The G*Power Team, Düsseldorf, Germany).

Lam et al. (17) randomly assigned 24 individuals to three groups: 1) control (weight maintenance, no intervention), 2) daily energy deficit (25%), and 3) daily energy deficit (25% plus exercise program). After a period of 6 months, the control group had a mean body weight of 82.3 ± 2.1 kg. The mean body weight after completion of the 6-month intervention was 73.2 ± 4.1 kg for group 2 (25% energy deficit). Based on these data, a group size of four subjects yields a statistical power of more than 95% to detect a difference of 9 kg.

The mean body weight after completion of the 6-month intervention was 75.7 ± 3.7 kg for group 3 (25% energy deficit plus exercise).

To illustrate potential differences between the treatment methods (app-based concept vs. in-person nutritional counseling), data obtained from groups 2 and 3 were used. Based on these data, a group size of 20 subjects yields a power of 80%. All other analyses were done exploratory.

Descriptive statistics were calculated for all variables, including means, standard deviations (SD), medians, as well as the first and third quartiles. Changes from baseline were analyzed after 12 and 16 weeks, respectively, using relative values. The Shapiro-Wilk test was applied to test for normality of the data. For normally distributed data, parametric tests were performed. Within-group comparisons over time were conducted using a one-factor repeated measures analysis of variance (ANOVA) or the Friedman test for non-parametric data, respectively. Between-group comparisons at individual time points were analyzed using one-factor ANOVA or Kruskal-Wallis test. For parameters assessed at only two time points, a paired *t*-test was applied for normally distributed data, whereas the Wilcoxon test was used when normality was not confirmed. Data are presented as medians (first quartile, third quartile). All *p*-values were corrected for multiple comparisons using the Benjamini-Hochberg procedure. The significance level was set at *p* < 0.05. We divided our study collective that followed an intervention (group 1–3) according to sex, body mass index (BMI;< 31 vs. ≥ 31), age (< 41 vs. ≥ 41), LA status (< 10.7 vs. ≥ 10.7 % FAME), ARA status (< 15.1 vs. ≥ 15.1 % FAME) and EPA status at baseline (< 0.7 % FAME vs. ≥ 0.7 % FAME) to evaluate the influence of these factors on the conversion of ALA into its LC metabolites. The limits represent the cut-off values of each 50 % of our participants (median). The statistical analysis of all data was performed using IBM SPSS Statistics software version 29.0.0.0 (IBM, Chicago, Illinois, USA), while plots were generated with R studio version 2022.12.0 (Public Benefit Corporation, Boston, Massachusetts, USA) running R software version 4.2.3 (The R Foundation for Statistical Computing, Vienna, Austria).

## Results

### Baseline characteristic

A total of 69 individuals completed the study to be included in the subsequent statistical analysis. This dropout rate of 13.8 % aligns with the projected range as determined through the power calculation (drop out: 25 %). Overall, 80 % of the participants were women. The distribution of age, weight and BMI was comparable between the groups at baseline (Table 1).

**TABLE 1:** Baseline characteristic of the PreVA subjects.

	Group 1		Group 2		Group 3		Control	
Sex [m/w]	15/3		14/5		11/4		14/2	
Age [years]	41 (± 8) 43 (37, 47)	a	38 (± 9) 38 (32, 45)	a	40 (± 8) 42 (38, 46)	a	38 (± 9) 40 (34, 44)	a
Weight [kg]	89.1 (± 20.0) 88.3 (73.4, 100.1)	a	91.8 (± 12.8) 90.6 (85.4, 102.1)	a	88.0 (± 13.6) 85.3 (79.5, 99.1)	a	88.4 (± 13.1) 87.9 (81.2, 93.2)	a
BMI [kg/m^2^]	30.9 (± 4.4) 31.2 (27.5, 33.5)	a	31.9 (± 3.3) 32.0 (29.4, 34.5)	a	30.0 (± 3.3) 29.9 (27.5, 32.0)	a	30.7 (± 3.6) 32.2 (28.2, 33.9)	a

Variable expressed as mean (± SD) and as median (25th, 75th percentile); groups without a common letter are significantly different, *p* < 0.05; BMI, Body mass index.

### Nutrient intake

The results of the 5-day dietary self-report before baseline assessment showed a comparable intake of energy, macronutrients and micronutrients between the four groups. In detail, group 2 exhibited a higher intake of total fatty acids (97.9 g/d) compared to group 3 (77.6 g/d) (*p* < 0.05). Furthermore, group 1 had higher intakes of EPA (109 mg/d) than group 3 (18 mg/d) (*p* = 0.03).

Compared to the reference values of the DGE, the average daily intake in all groups for total fatty acids, SFA, cholesterol and sodium was higher, while the intake of fiber, PUFA, vitamin A, vitamin D, vitamin E, folic acid, pantothenic acid, potassium and calcium was lower than recommended daily amounts (Supplementary Table 1).

### Anthropometric data and body composition during the intervention

During the reduction phase (12 weeks) the body weight dropped in all intervention groups (all *p* < 0.001) as well as the control group (*p* = 0.04). Weight reduction was significantly more pronounced in the three intervention groups compared to the control group after 12 weeks (−6.5; −7.3; −6.0 vs. −2.1 %; all *p* < 0.01) (Figure 3).

**FIGURE 3:**
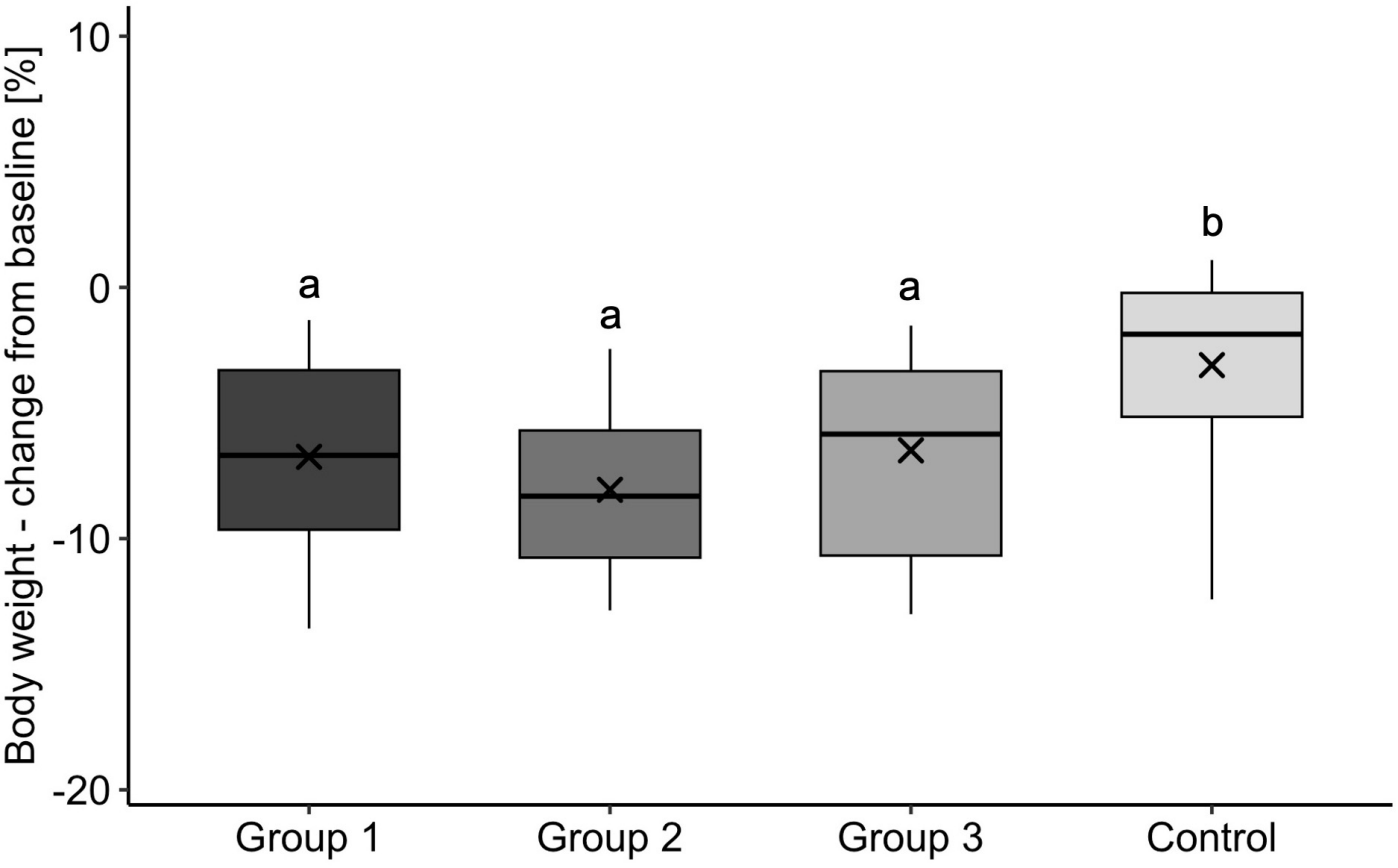
Comparison of percentual change from baseline of body weight after 16-weeks. The results are shown as box plot with median (indicated as line) and mean (indicated as cross). Groups that do not share indices (a,b) differ significantly [one-factor ANOVA or Kruskal-Wallis test depending on normal distribution followed by Benjamini-Hochberg correction (*p* < 0.05)].

Similarly, the BMI and waist circumference decreased in all intervention groups (all *p* < 0.001), while the control group only showed a reduction in waist circumference (*p* < 0.05). The reduction of BMI was more pronounced in the intervention groups compared to the control group (all *p* < 0.01), while the change from baseline of the waist circumference in group 2 and 3 was stronger compared to the control group (both *p* < 0.03). The significant reduction in all anthropometric parameters, including body weight, BMI, and waist circumference, could be maintained during the stabilization phase (16 weeks; all *p* < 0.01) and did even further improve in group 2 (*p* = 0.02). Notably, the change from baseline was still higher after 16 weeks in the intervention groups compared to the control group (*p* < 0.05). In total, 66.7 %, 78.9 %, and 53.3 % of subjects in group 1, 2, and 3 exhibited a weight loss greater than 5 % of their initial body weight. Besides, 25 % of the subjects in the control group demonstrated a weight loss of more than 5 % (Table 2).

**TABLE 2:** Anthropometric data and body composition during the intervention.

		Group 1 (*n* = 18)	T^1^	G^2^	Group 2 (*n* = 19)	T	G	Group 3 (*n* = 15)	T	G	Control (*n* = 16)	T	G
Anthropometric data													
Body weight [kg]	0	89.13 (± 20.05) 88.28 (73.41, 100.10)	a	a	91.83 (± 12.75) 90.60 (85.43, 102.10)	a	a	87.98 (± 13.44) 85.30 (79.48, 99.08)	a	a	88.39 (± 12.63) 87.88 (81.63, 93.16)	a	a
	4	86.25 (± 18.70) 86.25 (71.68, 96.90)	b	a	88.96 (± 12.41) 87.35 (83.25, 98.35)	b	a	85.94 (± 13.11) 83.25 (77.38, 96.68)	b	a	87.40 (± 12.81) 87.58 (78.96, 94.15)	a,b	a
	8	84.61 (± 18.09) 84.63 (71.36, 95.23)	c	a	87.25 (± 12.59) 84.55 (82.05, 94.93)	c	a	84.50 (± 12.70) 83.15 (75.88, 96.60)	c	a	86.79 (± 13.18) 87.30 (77.80, 92.39)	b	a
	12	83.19 (± 18.08) 82.68 (71.23, 94.11)	d	a	85.17 (± 12.37) 83.95 (79.40, 92.33)	d	a	82.66 (± 12.40) 80.95 (73.95, 93.43)	d	a	86.21 (± 13.42) 86.20 (75.21, 92.26)	b	a
	16	82.93 (± 17.94) 81.65 (70.83, 94.84)	d	a	84.50 (± 12.61) 83.20 (79.03, 91.28)	e	a	82.10 (± 11.86) 81.25 (72.20, 93.25)	d	a	85.41 (± 13.85) 84.98 (74.10, 89.68)	b	a
	%^0^→^12^	−6.48 (± 3.09) −6.63 (−8.46, −4.90)		a	−7.29 (± 2.64) −8.00 (−8.88, −5.41)		a	−5.96 (± 2.90) −5.27 (−8.79, −3.88)		a	−2.14 (± 3.21) 0.93 (−3.53, 0.16)		b
	%^0^→^16^	−6.74 (± 3.77) −6.69 (−9.65, −3.29)		a	−8.06 (± 3.04) −8.31 (−10.76, −5.70)		a	−6.49 (± 3.72) −5.84 (−10.68, −3.33)		a	−3.09 (± 3.85) −1.87 (−5.15, −0.22)		b
BMI [kg/m^2^]	0	30.92 (± 4.38) 31.20 (27.45, 33.46)	a	a	31.86 (± 3.30) 31.95 (29.41, 34.71)	a	a	30.04 (± 3.17) 29.94 (27.49, 31.55)	a	a	30.69 (± 3.46) 32.17 (28.18, 33.90)	a	a
	4	30.02 (± 4.11) 29.92 (26.59, 32.69)	b	a	30.88 (± 3.12) 30.80 (28.63, 33.62)	b	a	29.41 (± 3.07) 29.43 (26.51, 31.33)	b	a	30.28 (± 3.46) 30.84 (27.63, 32.70)	a	a
	8	29.55 (± 4.20) 29.83 (25.64, 31.87)	c	a	30.25 (± 3.23) 30.00 (28.01, 32.71)	c	a	29.01 (± 3.19) 28.70 (26.03, 30.52)	c	a	29.92 (± 3.43) 30.14 (27.34, 32.16)	a	a
	12	28.97 (± 4.08) 28.85 (25.80, 30.77)	d	a	29.52 (± 3.25) 29.74 (27.01, 31.83)	d	a	28.32 (± 3.06) 27.78 (25.63, 30.42)	d	a	29.85 (± 3.42) 30.33 (27.17, 32.36)	a	a
	16	28.92 (± 4.06) 29.17 (25.66, 30.84)	d	a	29.22 (± 3.25) 29.46 (26.99, 31.28)	e	a	28.21 (± 2.90) 27.29 (25.52, 30.56)	d	a	29.53 (± 3.38) 29.65 (26.98, 32.27)	a	a
	%^0^→^12^	−6.30 (± 2.94) −5.94 (−8.28, −4.90)		a	−7.36 (± 2.42) −8.00 (−8.85, −6.48)		a	−5.60 (± 3.43) −5.28 (−8.88, −2.98)		a	−2.25 (± 3.30) −1.29 (−3.54, 0.41)		b
	%^0^→^16^	−6.57 (± 3.53) −6.72 (−9.07, −3.28)		a	−8.30 (± 2.72) −8.30 (−10.76, −6.04)		a	−6.15 (± 4.12) −3.95 (−10.67, −2.93)		a,b	−3.14 (± 3.79) −1.86 (−5.10, −0.16)		b
Waist circumference [cm]	0	96.22 (± 13.60) 97.00 (91.25, 103.50)	a	a	99.00 (± 11.22) 99.50 (92.00, 106.50)	a	a	97.93 (± 11.36) 98.00 (89.00, 104.00)	a	a	96.39 (± 11.66) 92.75 (91.25, 101.75)	a,b	a
	4	95.39 (± 13.75) 93.50 (88.00, 101.50)	a	a	97.68 (± 11.76) 98.00 (90.75, 104.75)	a	a	96.10 (± 11.05) 97.00 (88.25,102.25)	a	a	97.04 (± 10.24) 95.75 (92.25, 100.38)	a	a
	8	91.36 (± 13.40) 91.00 (83.13, 97.50)	b	a	94.82 (± 10.68) 93.00 (87.50, 102.25)	b	a	93.50 (± 10.15) 94.50 (86.50, 97.50)	b	a	94.82 (± 11.22) 92.75 (90.25, 96.88)	b	a
	12	90.33 (± 12.85) 89.25 (84.00, 96.63)	b	a	92.13 (± 10.74) 89.00 (84.50, 98.75)	c	a	90.77 (± 7.97) 91.50 (85.00, 95.50)	c	a	93.11 (± 10.89) 91.00 (86.13, 96.88)	c	a
	16	88.72 (± 13.23) 88.25 (82.63, 94.88)	c	a	90.92 (± 10.36) 90.50 (86.25, 97.75)	c	a	89.20 (± 7.78) 88.00 (83.00, 95.25)	d	a	92.18 (± 11.70) 90.00 (85.38, 97.88)	c	a
	%^0^→^12^	−6.03 (± 4.13) −6.10 (−9.02, −3.61)		a,b	−6.90 (± 3.10) −6.84 (−9.69, −5.16)		a	−6.97 (± 4.30) −6.38 (−9.53, −3.62)		a	−3.14 (± 3.79) −4.08 (−4.86, −0.89)		b
	%^0^→^16^	−7.81 (± 3.82) −7.75 (−10.11, −5.63)		a	−8.05 (± 4.36) −8.44 (−10.37, −5.41)		a	−8.49 (± 5.51) −7.23 (−12.33, −3.92)		a	−4.26 (± 3.80) −4.09 (−5.64, −3.06)		b
Body composition													
Body fat [kg]	0	35.16 (± 12.56) 34.29 (26.04, 44.90)	a	a	37.33 (± 8.09) 38.90 (31.15, 44.05)	a	a	33.51 (± 9.28) 30.34 (27.46, 35.41)	a	a	34.93 (± 7.37) 34.09 (31.46, 41.54)	a	a
	4	32.54 (± 11.32) 33.00 (24.49, 41.37)	b	a	34.92 (± 8.01) 36.25 (28.71, 41.51)	b	a	31.74 (± 8.97) 28.22 (25.10, 34.78)	a,b	a	33.90 (± 7.66) 34.56 (30.34, 39.98)	b	a
	8	30.97 (± 11.31) 31.51 (21.48, 38.05)	c	a	33.34 (± 7.87) 34.30 (26.86, 39.56)	c	a	30.25 (± 8.73) 26.28 (24.10, 33.76)	b,c	a	32.62 (± 7.41) 32.41 (28.68, 39.30)	c	a
	12	30.32 (± 11.04) 29.99 (21.96, 36.28)	c,d	a	31.90 (± 8.36) 33.56 (25.25, 38.81)	d	a	29.27 (± 8.76) 25.08 (23.73, 32.75)	c	a	32.15 (± 7.32) 33.41 (26.61, 39.04)	c	a
	16	29.75 (± 10.74) 29.88 (21.21, 35.13)	d	a	30.98 (± 8.86) 33.53 (24.07, 37.59)	e	a	28.61 (± 8.22) 25.49 (23.46, 32.52)	c	a	32.14 (± 7.75) 33.20 (25.85, 37.63)	c	a
	%^0^→^12^	−14.58 (± 7.90) –15.34 (−21.37, −10.62)		a	−15.42 (± 7.08) −13.82 (−18.72, −10.10)		a	−13.78 (± 6.05) −15.52 (−17.52, −8.82)		a	−7.16 (± 6.45) −5.58 (−10.21, −3.34)		b
	%^0^→^16^	−16.24 (± 8.12) −17.15 (−22.93, −10.08)		a	−18.23 (± 9.84) −16.93 (−23.39, −10.11)		a	−15.53 (± 8.59) −12.93 (−23.58, −8.71)		a	−7.29 (± 7.03) −4.75 (−13.36, −1.92)		b
Viceral adipose tissue [l]	0	2.40 (± 1.63) 2.20 (1.55, 2.75)	a	a	2.65 (± 1.48) 2.40 (1.70, 3.20)	a	a	2.22 (± 1.22) 2.25 (1.33, 2.78)	a	a	2.23 (± 1.71) 1.70 (1.40, 2.30)	a	a
	4	2.31 (± 1.51) 1.80 (1.50, 2.95)	a	a	2.51 (± 1.42) 2.20 (1.55, 3.20)	a,b	a	1.98 (± 1.15) 1.80 (1.05, 2.60)	a,b	a	2.25 (± 1.59) 1.80 (1.50, 2.40)	a	a
	8	1.87 (± 1.36) 1.40 (1.05, 2.50)	b	a	2.14 (± 1.33) 1.85 (1.30, 2.55)	b	a	1.75 (± 0.97) 1.55 (1.00, 2.53)	b	a	1.98 (± 1.53) 1.50 (1.10, 2.50)	a,b	a
	12	1.82 (± 1.30) 1.30 (1.05, 2.35)	b	a	1.87 (± 1.32) 1.55 (1.05, 2.18)	c	a	1.48 (± 0.72) 1.40 (0.98, 2.10)	b,c	a	1.80 (± 1.38) 1.30 (1.10, 2.20)	b	a
	16	1.65 (± 1.22) 1.30 (1.00, 2.05)	b	a	1.76 (± 1.23) 1.50 (1.00, 1.98)	c	a	1.33 (± 0.76) 1.10 (0.78, 2.00)	c	a	1.85 (± 1.62) 1.20 (1.10, 2.30)	b	a
	%^0^→^12^	−28.71 (± 21.19) −29.02 (−40.41, −13.85)		a	−29.96 (± 16.56) −29.71 (−39.09, −22.92)		a	−30.77 (± 18.70) −32.80 (−40.88, −16.67)		a	−14.21 (± 24.21) −20.59 (−24.26, −12.95)		a
	%^0^→^16^	−35.89 (± 18.58) −38.10 (−45.45, −31.25)		a	−32.53 (± 22.20) −32.67 (−45.20, −22.55)		a	−37.05 (± 19.17) −45.24 (−49.68, −22.55)		a	−17.74 (± 23.24) −20.34 (−31.15, −12.40)		a
Body water [l]	0	40.18 (± 9.42) 37.70 (33.05, 42.83)	a	a	41.44 (± 7.02) 39.10 (36.60, 45.30)	a	a	38.13 (± 5.24) 36.80 (34.48, 41.30)	a	a	40.02 (± 6.18) 38.20 (35.60, 40.20)	a	a
	4	39.99 (± 9.51) 37.75 (32.63, 42.85)	a,b	a	40.96 (± 7.00) 38.85 (35.43, 45.50)	a,b	a	37.95 (± 5.31) 36.05 (34.40, 41.30)	a,c	a	39.96 (± 6.14) 38.00 (35.50, 42.40)	a	a
	8	40.18 (± 9.33) 37.65 (32.80, 43.35)	a	a	40.94 (± 6.86) 39.15 (35.30, 45.83)	a,b	a	37.93 (± 5.04) 36.40 (34.80, 41.45)	a	a	40.14 (± 6.42) 37.60 (36.20, 40.20)	a	a
	12	39.54 (± 8.88) 37.45 (32.65, 42.35)	b	a	40.41 (± 6.41) 38.75 (35.50, 45.60)	b	a	37.18 (± 4.94) 35.90 (33.55, 39.60)	b	a	40.38 (± 6.60) 38.00 (35.40, 40.50)	a	a
	16	39.91 (± 9.31) 37.55 (32.65, 42.85)	a,b	a	40.58 (± 7.00) 38.55 (34.55, 46.75)	b	a	37.30 (± 4.81) 35.90 (34.45, 40.15)	b,c	a	39.83 (± 6.55) 36.90 (35.80, 39.30)	a	a
	%^0^→^12^	−1.42 (± 1.71) −1.33 (−2.55, −0.31)		a	−2.33 (± 2.75) −2.48 (−3.34, −0.69)		a	−1.69 (± 3.65) −2.83 (−3.75, −0.91)		a	1.16 (± 2.13) 1.33 (−0.41, 2.20)		b
	%^0^→^16^	−1.01 (± 2.16) −1.09 (−2.29, 0.36)		a	−2.10 (± 2.66) −2.28 (−3.47, −1.77)		a	−1.76 (± 2.28) −1.95 (−3.58, 0.42)		a	−0.34 (± 2.65) −0.51 (−2.11, 1.48)		a
Lean body mass [kg]	0	25.90 (± 7.40) 23.26 (20.42, 28.15)	a	a	27.15 (± 5.56) 25.26 (23.07, 30.32)	a	a	24.54 (± 3.95) 23.22 (22.13, 26.93)	a	a	25.76 (± 4.79) 24.05 (22.75, 26.01)	a	a
	4	25.64 (± 7.39) 23.38 (20.05, 28.02)	a,b	a	26.73 (± 5.38) 25.12 (22.73, 30.20)	a,b	a	24.33 (± 3.95) 22.83 (21.76, 26.65)	a,b	a	25.79 (± 4.91) 24.16 (22.50, 27.84)	a	a
	8	25.51 (± 7.33) 23.03 (20.14, 27.84)	a,b	a	26.60 (± 5.28) 25.24 (22.23, 30.06)	b	a	24.13 (± 3.85) 23.02 (21.71, 26.30)	b	a	25.69 (± 5.13) 23.66 (22.40, 26.32)	a	a
	12	25.25 (± 6.87) 23.17 (20.07, 27.42)	a,b	a	26.17 (± 4.93) 24.97 (22.33, 29.91)	c	a	23.65 (± 3.79) 22.43 (21.22, 25.07)	c	a	25.89 (± 5.20) 23.76 (22.34, 26.12)	a	a
	16	25.33 (± 7.30) 22.81 (19.96, 27.96)	b	a	26.23 (± 5.41) 24.64 (21.96, 30.66)	c	a	23.51 (± 3.70) 22.20 (21.34, 25.08)	c	a	25.63 (± 5.19) 23.21 (22.64, 25.11)	a	a
	%^0^→^12^	−2.26 (± 2.50) −2.41 (−4.76, −0.01)		a	−3.29 (± 3.30) −3.02 (−4.77, −0.96)		a	−2.93 (± 3.67) −4.20 (−5.64, −0.66)		a	0.66 (± 2.79) 0.42 (−1.21, 2.45)		b
	%^0^→^16^	−2.66 (± 2.97) −2.46 (−5.20, −1.29)		a	−3.38 (± 3.23) −3.17 (−5.55, −2.35)		a	−3.72 (± 2.77) −4.39 (−6.20, −1.27)		a	−0.76 (± 3.73) −0.66 (−3.47, 1.96)		a
Meatbolic rate [kcal/d]	0	1667.1 (± 309.4) 1601.0 (1440.5, 1855.3)	a	a	1738.5 (± 205.9) 1726.0 (1607.3, 1837.0)	a	a	1616.2 (± 207.8) 1555.5 (1469.3, 1816.0)	a	a	1659.5 (± 233.1) 1589.0 (1516.0, 1775.0)	a	a
	4	1634.8 (± 288.2) 1578.0 (1416.5, 1819.5)	b	a	1706.2 (± 200.7 1691.0 (1556.5, 1810.5)	b	a	1593.00 (± 202.2) 1529.50 (1443.25, 1776.25)	b	a	1648.1 (± 233.9) 1579.0 (1476.0, 1772.0)	a	a
	8	1618.9 (± 281.2) 1554.5 (1398.5, 1792.8)	c	a	1686.7 (± 195.7) 1674.0 (1547.3, 1756.0)	c	a	1575.92 (± 197.26) 1512.50 (1426.25, 1745.50)	c	a	1636.6 (± 239.3) 1571.0 (1463.0, 1759.0)	a	a
	12	1606.1 (± 278.9) 1521.5 (1398.5, 1774.0)	d	a	1665.3 (± 189.8) 1656.5 (1538.3, 1737.8)	d	a	1555.2 (± 193.5) 1502.5 (1402.50, 1698.3)	d	a	1635.9 (± 241.0) 1589.0 (1451.0, 1736.0)	a	a
	16	1608.6 (± 279.2) 1531.5 (1396.3, 1767.0)	c,d	a	1656.0 (± 191.3) 1645.5 (1521.5, 1735.8)	e	a	1549.4 (± 188.3) 1494.0 (1382.0, 1671.8)	d	a	1625.8 (± 248.8) 1586.0 (1430.0, 1683.0)	a	a
	%^0^→^12^	−3.68 (± 2.01) −4.18 (−5.26, −1.75)		a	−4.13 (± 1.98) −3.95 (−5.76, −2.67)		a	−3.70 (± 1.95) −3.70 (−4.98, −2.07)		a	−1.22 (± 1.82) −0.72 (−2.14, 0.13)		b
	%^0^→^16^	−3.70 (± 2.34) −3.55 (−5.36, −1.55)		a,b	−4.68 (± 2.18) −4.18 (−6.47, −3.03)		a	−4.15 (± 2.48) −3.76 (−6.23, −2.20)		a	−1.79 (± 2.24) −0.63 (−3.33, 0.12)		b

Variable expressed as mean (± SD) and as median (25th, 75th percentile);

^1^comparison of time points within the respective diet group (T, column by column);

^2^comparison between diet groups at individual time points (G; line by line); groups without a common letter are significantly different, *p* < 0.05; BMI, body mass index.

Most of the weight loss was due to the reduction in body fat, which was gradual in all intervention groups (all *p* < 0.001) and the control group (*p* < 0.01). This was shown after the reduction phase and maintained through the stabilization phase. The loss of body fat was more pronounced in the three intervention groups compared to the control (all *p* < 0.03). Visceral adipose tissue decreased significantly in all groups, reaching the lowest values after 16 weeks (all *p* < 0.02), but there were no differences in the change from baseline after 12 and 16 weeks between the intervention and control groups. Body water, lean mass, and resting energy expenditure showed slight but significant decreases in all intervention groups compared to baseline (all *p* < 0.05). These changes were maintained during the stabilization phase and still more pronounced in the intervention groups (*p* < 0.05). The control group, however, did not exhibit significant changes in these parameters. However, by the end of 12 and 16 weeks, respectively, there were no between group differences in any of the investigated parameters (Table 2).

### Cardiovascular risk factors during the intervention

At the start of the intervention, all groups exhibited a blood pressure in the normal to high normal range (Table 3). Over the course of the study, blood pressure improved in the intervention groups with a reduction of systolic blood pressure by −6.7 to −8.4 % (*p* < 0.01) and diastolic blood pressure by −9.1 to −12.6 % (*p* ≤ 0.02) after the reduction phase. While systolic blood pressure remained lower in all groups after 16 weeks (all *p* < 0.01), diastolic blood pressure was only lower in group 2 and 3 compared to baseline (both *p* < 0.02). The control group did not improve their blood pressure after 12 and 16 weeks, respectively. Despite these differences, neither the change from baseline nor the absolute values differed significantly between the four groups at 12 or 16 weeks (Table 3).

**TABLE 3:** Cardiovascular risk factors during the intervention.

		Group 1 (*n* = 18)	T^1^	G^2^	Group 2 (*n* = 19)	T	G	Group 3 (*n* = 15)	T	G	Control (*n* = 16)	T	G
Blood pressure													
Systolic blood pressure [mmHG]	0	126.83 (± 16.06) 126.00 (115.50, 132.75)	a	a	128.11 (± 15.98) 129.00 (119.50, 132.50)	a	a	127.00 (± 10.88) 126.00 (121.50, 135.50)	a	a	125.71 (± 12.95) 124.50 (118.00, 128.75)	a	a
	4	121.67 (± 14.05) 119.00 (113.25, 129.50)	a,b	a	117.79 (± 11.81) 121.00 (111.50, 124.00)	b	a	120.73 (± 9.23) 122.00 (115.00, 127.00)	b	a	121.29 (± 10.14) 120.50 (114.75, 122.75)	a	a
	8	120.94 (± 15.09) 120.50 (114.00, 130.00)	a,b	a	116.47 (± 9.88) 115.00 (110.50, 125.00)	b	a	115.40 (± 8.88) 114.00 (108.50, 121.50)	c	a	117.36 (± 11.32) 117.50 (107.00, 126.25)	a	a
	12	117.89 (± 13.31) 116.00 (109.25, 126.00)	b	a	116.74 (± 11.17) 120.00 (107.50, 124.50)	b	a	116.67 (± 9.05) 114.00 (111.00, 122.00)	b,c	a	117.29 (± 12.10) 116.00 (108.50, 125.50)	a	a
	16	119.83 (± 13.92) 118.00 (110.50, 124.75)	b	a	117.58 (± 8.19) 120.00 (113.00, 123.00)	b	a	118.33 (± 9.31) 116.00 (113.00, 126.50)	b,c	a	118.86 (± 11.95) 117.00 (110.00, 125.50)	a	a
	%^0^→^12^	−6.72 (± 6.76) −4.46 (−10.87, −3.02)		a	−8.37 (± 7.19) −7.69 (−11.04, −2.62)		a	−7.85 (± 6.32) −7.38 (−12.26, −4.86)		a	−5.02 (± 8.96) −5.79 (−13.13, 1.03)		a
	%^0^→^16^	−5.28 (± 5.77) −4.92 (−8.22, −3.08)		a	−7.49 (± 7.45) −6.87 (−11.16, −2.47)		a	−6.46 (± 7.61) −8.20 (−12.56, 0.84)		a	−4.50 (± 8.38) −3.69 (−8.79, −0.07)		a
Diastolic blood pressure [mmHG]	0	87.17 (± 10.49) 87.00 (80.00, 92.50)	a	a	88.37 (± 9.39) 89.00 (79.50, 94.00)	a	a	89.80 (± 8.30) 89.00 (84.00, 96.00)	a	a	86.14 (± 11.95) 85.00 (77.25, 92.75)	a	a
	4	81.89 (± 13.63) 79.00 (72.25, 91.25)	a,b	a	80.21 (± 8.64) 82.00 (73.50, 86.50)	b	a	81.67 (± 8.42) 82.00 (77.00, 85.50)	b	a	85.07 (± 12.22) 78.50 (77.25, 89.50)	a	a
	8	82.94 (± 11.14) 82.00 (73.75, 88.75)	a,b	a	80.26 (± 9.36) 78.00 (74.00, 86.50)	b	a	77.27 (± 7.96) 77.00 (72.00, 82.50)	b	a	80.00 (± 10.16) 79.00 (72.25, 89.00)	a	a
	12	79.17 (± 13.25) 76.00 (70.00, 87.50)	b	a	78.79 (± 8.57) 80.00 (71.50, 85.50)	b	a	78.13 (± 8.57) 74.00 (73.00, 83.00)	b	a	81.36 (± 14.02) 83.00 (70.25, 93.75)	a	a
	16	83.72 (± 11.21) 81.50 (76.50, 88.75)	a	a	80.42 (± 9.32) 80.00 (74.00, 86.50)	b	a	78.27 (± 8.54) 76.00 (72.00, 85.00)	b	a	81.21 (± 10.81) 82.50 (72.50, 86.00)	a	a
	%^0^→^12^	−9.08 (± 11.57) −8.44 (−16.94, −3.24)		a	−10.40 (± 9.58) −12.35 (−15.91, −6.25)		a	−12.64 (± 9.41) −13.40 (−19.99, −8.87)		a	−6.01 (± 8.78) −3.64 (−10.90, 0.00)		a
	%^0^→^16^	−3.38 (± 12.80) −5.25 (−11.66, 2.21)		a	−8.63 (± 8.80) −7.61 (−11.80, −4.43)		a	−12.26 (± 10.89) −10.13 (−17.76, −8.06)		a	−4.71 (± 8.77) −4.05 (−8.51, 0.27)		a
Blood lipids													
Total cholesterol [mmol/L]	0	4.85 (± 0.94) 4.92 (4.39, 5.37)	a	a	4.65 (± 0.62) 4.62 (4.30, 5.15)	a	a	5.01 (± 0.59) 5.06 (4.67, 5.54)	a	a	4.75 (± 0.86) 4.83 (4.05, 5.06)	a	a
	4	4.35 (± 0.76) 4.48 (3.94, 4.66)	b	a	4.00 (± 0.59) 4.14 (3.75, 4.38)	b	a	4.43 (± 0.61) 4.44 (4.00, 4.94)	b	a	4.56 (± 0.77) 4.55 (3.85, 5.01)	a,b	a
	8	4.34 (± 0.86) 4.16 (3.88, 5.16)	b	a	3.98 (± 0.59) 4.10 (3.65, 4.26)	b	a	4.34 (± 0.66) 4.25 (3.89, 4.80)	b	a	4.51 (± 0.73) 4.41 (4.03, 4.68)	a,b	a
	12	4.48 (± 0.94) 4.58 (3.93, 5.08)	b	a	4.07 (± 0.59) 4.19 (3.69, 4.43)	b	a	4.23 (± 0.59) 4.22 (3.95, 4.45)	b	a	4.43 (± 0.76) 4.25 (3.93, 4.98)	b	a
	16	4.54 (± 0.89) 4.66 (4.03, 5.17)	a,b	a	4.03 (± 0.63) 4.12 (3.72, 4.44)	b	a	4.41 (± 0.66) 4.42 (4.20, 4.87)	b	a	4.78 (± 0.78) 4.80 (4.20, 5.07)	a	a
	%^0^→^12^	−7.55 (± 9.98) −10.43 (−14.12, 1.43)		a	−13.35 (± 9.93) −14.86 (−19.88, −9.60)		a	−15.09 (± 9.62) −12.93 (−22.64, −8.55)		a	−5.99 (± 7.39) −6.40 (−10.68, −1.51)		a
	%^0^→^16^	−6.11 (± 9.71) −8.78 (−15.06, 1.51)		a,b	−12.96 (± 9.04) −13.59 (−18.14, −5.84)		a	−11.78 (± 10.06) −9.29 (−16.25, −4.92)		a	−0.55 (± 9.21) −2.81 (−6.32, 2.53)		b
Non-HDL-cholesterol [mmol/L]	0	3.28 (± 1.19) 3.55 (2.26, 3.80)	a	a	3.17 (± 0.71) 3.10 (2.85, 3.55)	a	a	3.58 (± 0.71) 3.62 (3.27, 4.01)	a	a	3.18 (± 0.95) 3.11 (2.55, 3.44)	a	a
	4	2.93 (± 0.87) 3.12 (2.21, 3.46)	a	a	2.73 (± 0.66) 2.60 (2.27, 3.21)	b	a	3.15 (± 0.72) 3.04 (2.69, 3.79)	b	a	3.03 (± 0.92) 2.95 (2.39, 3.23)	a	a
	8	2.93 (± 0.99) 2.89 (2.23, 3.74)	a	a	2.67 (± 0.59) 2.60 (2.24, 3.12)	b	a	3.10 (± 0.68) 3.02 (2.72, 3.27)	b	a	2.99 (± 0.85) 2.76 (2.45, 3.35)	a	a
	12	3.06 (± 1.04) 3.02 (2.40, 3.66)	a	a	2.75 (± 0.63) 2.80 (2.33, 3.22)	b	a	3.01 (± 0.61) 2.95 (2.56, 3.22)	b	a	2.96 (± 0.88) 2.92 (2.51, 3.36)	a	a
	16	3.07 (± 1.00) 3.23 (2.51, 3.57)	a	a	2.70 (± 0.60) 2.63 (2.43, 3.16)	b	a	3.10 (± 0.67) 3.08 (2.76, 3.27)	b	a	3.26 (± 0.93) 3.14 (2.72, 3.63)	a	a
	%^0^→^12^	−5.16 (± 13.32) −6.10 (−13.22, 3.89)		a	−12.14 (± 13.65) −13.33 (−17.88, −9.43)		a	−14.68 (± 12.59) −16.82 (−25.21, −1.43)		a	−6.69 (± 10.02) −8.72 (−10.29, 1.07)		a
	%^0^→^16^	−4.69 (± 12.52) −9.92 (−15.00, 1.91)		a	−13.49 (± 13.60) −9.12 (−20.75, −3.74)		a	−12.52 (± 12.90) −12.80 (−21.15, −5.13)		a	3.33 (± 12.90) −0.96 (−6.63, 13.35)		a
HDL cholesterol [mmol/L]	0	1.57 (± 0.41) 1.57 (1.30, 1.89)	a	a	1.49 (± 0.31) 1.41 (1.36, 1.61)	a	a	1.43 (± 0.38) 1.41 (1.08, 1.62)	a	a	1.56 (± 0.41) 1.56 (1.33, 1.81)	a	a
	4	1.42 (± 0.32) 1.33 (1.20, 1.75)	b	a	1.27 (± 0.24) 1.19 (1.12, 1.43)	b	a	1.27 (± 0.30) 1.15 (1.09, 1.40)	a	a	1.53 (± 0.43) 1.55 (1.36, 1.65)	a	a
	8	1.41 (± 0.28) 1.37 (1.23, 1.71)	b	a	1.31 (± 0.28) 1.25 (1.13, 1.47)	b	a	1.24 (± 0.27) 1.17 (1.06, 1.32)	a	a	1.52 (± 0.37) 1.55 (1.32, 1.82)	a	a
	12	1.42 (± 0.31) 1.42 (1.17, 1.68)	b	a	1.32 (± 0.29) 1.25 (1.14, 1.46)	b	a	1.22 (± 0.25) 1.20 (1.08, 1.39)	a	a	1.47 (± 0.35) 1.46 (1.21, 1.70)	a	a
	16	1.46 (± 0.32) 1.42 (1.26, 1.77)	a,b	a	1.34 (± 0.26) 1.31 (1.15, 1.53)	b	a	1.30 (± 0.31) 1.20 (1.12, 1.47)	a	a	1.51 (± 0.36) 1.58 (1.27, 1.73)	a	a
	%^0^→^12^	−7.63 (± 12.03) −8.87 (−15.80, −0.15)		a	−10.49 (± 15.25) −15.89 (−18.56, −7.24)		a	−12.62 (± 13.65) −14.18 (−22.00, −6.00)		a	−5.18 (± 9.61) −4.25 (−9.40, −0.86)		a
	%^0^→^16^	−5.08 (± 10.65) −3.32 (−10.53, 1.19)		a	−9.65 (± 10.86) −10.11 (−15.46, −6.55)		a	−6.81 (± 14.31) −7.35 (−18.43, 4.93)		a	−3.61 (± 10.08) −6.07 (−9.74, 1.42)		a
LDL cholesterol [mmol/L]	0	3.12 (± 1.03) 3.39 (2.43, 3.52)	a	a	2.98 (± 0.61) 2.90 (2.66, 3.35)	a	a	3.36 (± 0.59) 3.36 (3.12, 3.67)	a	a	2.88 (± 0.76) 2.73 (2.31, 3.31)	a	a
	4	2.64 (± 0.82) 2.70 (2.20, 3.29)	b	a	2.44 (± 0.55) 2.46 (2.01, 2.90)	b	a	2.80 (± 0.56) 2.71 (2.39, 3.37)	b	a	2.58 (± 0.55) 2.43 (2.22, 2.98)	a,b	a
	8	2.60 (± 0.84) 2.63 (1.99, 3.23)	b	a	2.32 (± 0.52) 2.35 (1.98, 2.77)	b	a	2.71 (± 0.54) 2.65 (2.33, 3.01)	b,c	a	2.63 (± 0.66) 2.37 (2.29, 2.92)	a,b	a
	12	2.61 (± 0.84) 2.68 (2.13, 3.10)	b	a	2.29 (± 0.53) 2.15 (1.96, 2.58)	b	a	2.53 (± 0.51) 2.50 (2.09, 2.80)	c	a	2.43 (± 0.69) 2.26 (2.03, 2.81)	b	a
	16	2.72 (± 0.87) 2.78 (2.25, 3.28)	b	a	2.30 (± 0.49) 2.20 (2.06, 2.75)	b	a	2.70 (± 0.55) 2.58 (2.44, 2.98)	b,c	a	2.69 (± 0.72) 2.60 (2.32, 2.94)	a	a
	%^0^→^12^	−15.86 (± 11.30) −5.02 (−25.86, −8.93)		a	−23.93 (± 13.21) −21.34 (−31.86, −16.18)		a	−23.71 (± 12.03) −19.35 (−34.39, −13.30)		a	−14.96 (± 8.02) −15.29 (−18.75, −11.19)		a
	%^0^→^16^	−12.31 (± 9.96) −14.03 (−19.60, −3.41)		a,b	−21.18 (± 12.59) −16.22 (−32.12, −10.86)		a	−19.08 (± 11.53) −16.67 (−25.98, −10.99)		a	−7.70 (± 12.65) −8.29 (−20.91, 1.99)		b
TAG [mmol/L]	0	1.18 (± 0.65) 0.96 (0.64, 1.71)	a	a	0.98 (± 0.48) 0.85 (0.63, 1.19)	a	a	1.11 (± 0.47) 1.09 (0.70, 1.48)	a	a	1.36 (± 0.52) 1.26 (1.03, 1.72)	a	a
	4	1.01 (± 0.51) 0.77 (0.66, 1.38)	a,b	a	0.93 (± 0.34) 0.89 (0.69, 1.10)	a	a	1.18 (± 0.70) 0.87 (0.76, 1.48)	a	a	1.48 (± 0.83) 1.17 (0.90, 1.93)	a	a
	8	0.97 (± 0.57) 0.75 (0.70, 0.97)	a,b	a	0.94 (± 0.44) 0.81 (0.66, 0.97)	a	a	1.10 (± 0.67) 0.97 (0.68, 1.12)	a	a	1.23 (± 0.51) 1.07 (0.85, 1.61)	a	a
	12	1.08 (± 0.62) 0.79 (0.75, 1.32)	a,b	a	0.93 (± 0.53) 0.76 (0.67, 1.05)	a	a	1.02 (± 0.45) 0.98 (0.69, 1.05)	a	a	1.28 (± 0.61) 1.19 (0.82, 1.78)	a	a
	16	0.89 (± 0.40) 0.75 (0.64, 1.24)	b	a	0.94 (± 0.39) 0.80 (0.72, 1.04)	a	a	1.12 (± 0.47) 0.92 (0.75, 1.35)	a	a	1.29 (± 0.61) 1.24 (0.80, 1.44)	a	a
	%^0^→^12^	−1.96 (± 29.85) −5.00 (−25.02, 16.39)		a	−0.83 (± 32.86) −6.72 (−16.05, 15.87)		a	−3.88 (± 25.41) −8.62 (−20.45, 17.17)		a	−2.36 (± 32.10) −17.53 (−28.59, 20.39)		a
	%^0^→^16^	1.18 (± 0.65) 0.96 (0.64, 1.71)		a	2.04 (± 28.84) −1.06 (−14.91, 16.76)		a	6.55 (± 30.26) −3.40 (−13.42, 18.15)		a	−6.86 (± 31.20) −19.77 (−23.81, −1.63)		a
Inflammation													
hsCRP [mg/L]	0	2.51 (± 3.28) 1.30 (0.90, 2.20)	a	a	1.76 (± 1.26) 1.10 (0.80, 2.50)	a	a	1.75 (± 1.34) 1.40 (0.70, 2.10)	a	a	3.09 (± 2.28) 2.15 (1.50, 4.67)	a	a
	4	2.22 (± 3.16) 1.00 (0.60, 1.40)	a,b	a	1.01 (± 0.71) 0.80 (0.50, 1.20)	b	a	1.26 (± 1.03) 0.80 (0.65, 1.60)	a	a	1.97 (± 1.86) 1.25 (0.58, 2.60)	a	a
	8	2.04 (± 2.80) 0.90 (0.60, 1.50)	b	a	1.27 (± 0.73) 1.00 (0.70, 1.80)	a,b	a	2.60 (± 4.91) 0.90 (0.60, 1.45)	a	a	2.31 (± 1.45) 2.15 (1.13, 3.53)	a	a
	12	2.26 (± 3.14) 1.00 (0.70, 3.10)	a,b	a	1.08 (± 0.62) 0.90 (0.70, 1.20)	a,b	a	1.25 (± 1.34) 0.90 (0.55, 1.20)	a	a	2.76 (± 2.15) 2.20 (1.03, 4.53)	a	a
	16	2.02 (± 2.98) 1.20 (0.70, 1.60)	a,b	a	1.66 (± 1.03) 1.20 (0.90, 2.20)	a	a	1.30 (± 0.94) 1.00 (0.70, 1.60)	a	a	2.59 (± 2.10) 1.70 (0.85, 4.28)	a	a
	%^0^→^12^	−8.35 (± 33.18) −6.69 (−30.77, 8.33)		a	−21.66 (± 51.13) −36.36 (−56.19, −4.00)		a	−8.61 (± 72.87) −20.00 (−44.05, −10.80)		a	134.87 (± 569.27) −7.50 (−43.25, 17.80)		a
	%^0^→^16^	−5.89 (± 52.96) −15.56 (−35.61, −1.10)		a	6.81 (± 67.08) −10.00 (−30.00, 10.10)		a	−12.99 (± 44.30) −25.00 (−35.71, −5.56)		a	−9.81 (± 30.94) −1.47 (−42.31, 20.23)		a
Markers of glucose metabolism													
Glucose [mmol/L]	0	5.52 (± 0.49) 5.55 (5.17, 5.80)	a	a	5.24 (± 0.50) 5.30 (4.80, 5.50)	a	a	5.40 (± 0.46) 5.30 (5.10, 5.50)	a	a	5.19 (± 0.43) 5.30 (4.90, 5.45)	a	a
	4	5.53 (± 0.51) 5.45 (5.25, 5.73)	a	a	5.09 (± 0.34) 5.10 (4.90, 5.30)	a	a	5.28 (± 0.49) 5.10 (5.00, 5.40)	a	a	5.15 (± 0.41) 5.00 (4.88, 5.40)	a	a
	8	5.31 (± 0.38) 5.35 (5.00, 5.70)	a,b	a	5.26 (± 0.32) 5.20 (5.00, 5.50)	a	a	5.31 (± 0.41) 5.30 (5.00, 5.50)	a	a	5.26 (± 0.39) 5.15 (4.98, 5.63)	a	a
	12	5.41 (± 0.46) 5.40 (5.10, 5.80)	a,b	a	5.19 (± 0.35) 5.20 (5.10, 5.40)	a	a	5.23 (± 0.36) 5.20 (5.05, 5.40)	a	a	5.25 (± 0.41) 5.20 (5.05, 5.55)	a	a
	16	5.23 (± 0.30) 5.20 (5.00, 5.40)	b	a	5.21 (± 0.37) 5.20 (4.90, 5.50)	a	a	5.31 (± 0.31) 5.20 (5.00, 5.55)	a	a	5.39 (± 0.40) 5.35 (5.20, 5.63)	a	a
	%^0^→^12^	−2.27 (± 6.03) −2.63 (−5.33, 1.47)		a	0.07 (± 9.33) 0.00 (−4.61, 7.23)		a	−2.72 (± 7.26) −1.85 (−8.99, 1.91)		a	−0.30 (± 6.43) −0.86 (−2.92, 3.61)		a
	%^0^→^16^	−5.32 (± 5.59) −3.64 (−8.93, −1.82)		a	−0.17 (± 7.52) 0.00 (−5.51, 2.85)		a,b	−1.33 (± 6.54) 1.79 (−3.85, 2.93)		a,b	3.49 (± 5.26) 2.67 (0.45, 5.88)		b
Insulin [mU/L]	0	13.21 (± 6.19) 10.10 (9.40, 19.40)	a	a	11.33 (± 4.90) 10.30 (8.90, 12.20)	a	a	12.06 (± 4.67) 13.50 (8.70, 15.20)	a	a	11.61 (± 5.44) 10.85 (7.07, 13.30)	a	a
	4	9.90 (± 4.44) 9.00 (7.90, 12.00)	b	a	10.01 (± 4.69) 9.30 (7.20, 10.50)	a,b	a	10.07 (± 5.26) 8.70 (7.10, 10.90)	a	a	12.15 (± 8.63) 9.25 (5.95, 14.50)	a	a
	8	9.14 (± 4.57) 8.90 (5.70, 11.60)	b	a	11.15 (± 5.93) 10.00 (8.30, 13.90)	a	a	11.01 (± 6.66) 9.90 (6.65, 11.50)	a	a	12.21 (± 4.32) 11.55 (9.48, 14.95)	a	a
	12	8.69 (± 3.73) 8.10 (6.20, 10.20)	b	a	9.21 (± 6.65) 6.30 (4.70, 10.30)	b	a	8.91 (± 5.07) 7.50 (5.40, 9.70)	a	a	10.01 (± 3.85) 9.75 (8.10, 12.88)	a	a
	16	7.77 (± 3.92) 7.10 (5.10, 10.30)	b	a	9.18 (± 5.99) 6.80 (5.50, 12.30)	b	a	10.87 (± 6.21) 10.00 (7.65, 11.65)	a	a	9.82 (± 4.40) 8.80 (6.45, 14.53)	a	a
	%^0^→^12^	−31.10 (± 24.51) −31.94 (−52.71, −21.72)		a	−19.68 (± 29.19) −21.82 (−38.18, 1.02)		a	−19.13 (± 41.33) −44.03 (−47.65, 0.01)		a	−6.55 (± 28.49) −9.77 (−22.21, 2.91)		a
	%^0^→^16^	−38.65 (± 23.65) −42.76 (−48.63, −33.97)		a	−15.56 (± 31.04) −22.73 (−40.21, 9.54)		b	0.70 (± 49.20) −6.02 (−37.24, 31.33)		b	−9.76 (± 26.82) −18.54 (−24.94, 16.24)		b
HOMA-IR	0	3.31 (± 1.72) 2.45 (2.22, 5.09)	a	a	2.68 (± 1.30) 2.29 (2.02, 3.14)	a	a	2.95 (± 1.31) 3.00 (2.03, 3.86)	a	a	2.52 (± 1.39) 2.27 (1.41, 2.90)	a	a
	4	2.46 (± 1.26) 2.18 (1.75, 2.83)	a,b	a	2.29 (± 1.18) 2.15 (1.63, 2.54)	a,b	a	2.38 (± 1.36) 2.10 (1.69, 2.47)	a	a	2.90 (± 2.41) 2.14 (1.05, 3.42)	a	a
	8	2.19 (± 1.19) 1.99 (1.31, 2.87)	b,c	a	2.65 (± 1.54) 2.37 (1.90, 3.46)	a	a	2.65 (± 1.77) 2.33 (1.54, 2.82)	a	a	2.72 (± 1.10) 2.49 (2.10, 3.64)	a	a
	12	2.03 (± 0.95) 1.87 (1.49, 2.54)	b,c	a	2.16 (± 1.65) 1.51 (1.15, 2.66)	b	a	2.12 (± 1.39) 1.70 (1.26, 2.40)	a	a	2.33 (± 1.12) 2.14 (1.70, 3.06)	a	a
	16	1.74 (± 0.92) 1.53 (1.21, 2.05)	c	a	2.18 (± 1.61) 1.43 (1.27, 3.17)	b	a	2.60 (±1.68) 2.24 (1.87, 2.80)	a	a	2.35 (±1.21) 2.15 (1.43, 3.59)	a	a
	%^0^→^12^	−31.87 (±27.18) −33.51 (−53.84, −23.75)		a	−17.77 (±35.11) −18.75 (−36.81, 0.13)		a	−19.52 (±47.96) −45.60 (−51.30, −2.44)		a	−5.44 (± 35.10) −8.12 (−25.34, 3.69)		a
	%^0^→^16^	−43.54 (± 23.97) −45.02 (−54.37, −38.30)		a	−14.41 (± 36.56) −22.61 (−42.99, 10.71)		b	0.90 (± 53.45) −3.23 (−40.60, 33.38)		b	−3.57 (± 27.25) −13.87 (−24.88, 21.51)		b
HbA1c [%]	0	5.36 (± 0.46) 5.35 (5.10, 5.73)	a	a	5.28 (± 0.33) 5.40 (5.20, 5.50)	a,b	a	5.23 (± 0.23) 5.20 (5.05, 5.30)	a	a	5.22 (± 0.30) 5.20 (5.00, 5.50)	a,b	a
	4	5.27 (± 0.47) 5.25 (5.10, 5.50)	a	a	5.24 (± 0.32) 5.30 (5.10, 5.40)	a,b	a	5.15 (± 0.28) 5.10 (4.95, 5.30)	a,b	a	5.19 (± 0.30) 5.20 (5.00, 5.40)	a,b	a
	8	5.21 (± 0.46) 5.20 (4.98, 5.50)	b	a	5.21 (± 0.32) 5.30 (5.10, 5.40)	a	a	5.10 (± 0.27) 5.00 (4.90, 5.30)	b	a	5.15 (± 0.30) 5.10 (4.90, 5.30)	a	a
	12	5.27 (± 0.43) 5.25 (5.08, 5.45)	a	a	5.22 (± 0.31) 5.30 (5.10, 5.40)	a,b	a	5.12 (± 0.25) 5.10 (4.95, 5.20)	b	a	5.17 (± 0.29) 5.10 (5.00, 5.30)	a	a
	16	5.34 (± 0.45) 5.25 (5.18, 5.70)	a	a	5.32 (± 0.29) 5.40 (5.20, 5.50)	b	a	5.18 (± 0.27) 5.10 (5.00, 5.35)	a,b	a	5.30 (± 0.29) 5.30 (5.10, 5.50)	b	a
	%^0^→^12^	−1.38 (± 3.28) −0.96 (−3.43, 0.00)		a	−1.60 (±2.59) –1.82 (–3.70, 0.00)		a	–2.04 (±2.26) –1.89 (–3.96, –0.88)		a	–0.93 (± 2.07) 0.00 (–2.01, 0.00)		a
	%^0^→^16^	–0.15 (± 3.48) 0.00 (–1.72, 1.96)		a	0.38 (± 2.04) 0.00 (–0.86, 1.85)		a	–0.90 (± 2.54) –1.82 (–2.91, 0.00)		a	1.61 (± 3.00) 0.00 (0.00, 4.17)		a

Variable expressed as mean (± SD) and as median (**25th,** 75**th** percentile);

^1^comparison of time points within the respective diet group (T, column by column);

^2^comparison between diet groups at individual time points (G; line by line); groups without a common letter are significantly different, *p* < 0.05: HDL, high density lipoprotein; LDL, low density lipoprotein; TAG, triacylglyceride; hsCRP, high sensitive C-reactive protein; HOMA-IR, Homeostasis Model Assessment of Insulin Resistance.

Regarding blood lipids, total and LDL cholesterol decreased in all intervention groups (all *p* < 0.05) as well as the control after 12 weeks (*p* < 0.05). The change from baseline of total cholesterol was greater in group 1 (−10.4 %), group 2 (−14.9 %) and group 3 (−13.0 %) compared to the control group (−6.4 %) but did not reach statistical significance. Following the stabilization phase, group 2 and 3 remained a greater change from baseline for both parameters compared to the control (both *p* ≤ 0.03), which showed comparable values to baseline (Figure 4).

**FIGURE 4:**
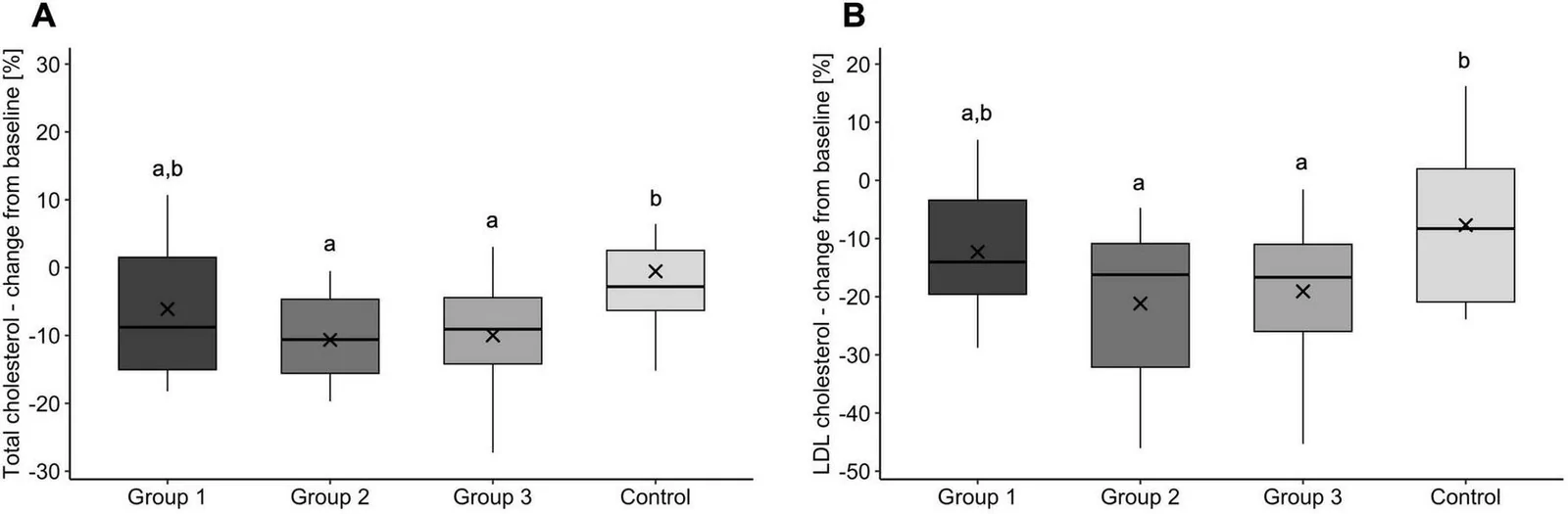
Comparison of percentual change from baseline of **(A)** total cholesterol and **(B)** LDL cholesterol after 16-weeks. The results are shown as box plot with median (indicated as line) and mean (indicated as cross). Groups that do not share indices (a,b) differ significantly [one-factor ANOVA or Kruskal-Wallis test depending on normal distribution followed by Benjamini-Hochberg correction (*p* < 0.05)].

Initially, 35.3 %, 23.5 %, and 42.9 % of subjects in groups 1, 2, and 3, respectively, exhibited elevated total cholesterol concentrations (> 5.2 mmol/L). Notably, 58.8 %, 47.1 %, and 78.6 % of these subjects exceeded the reference value for LDL cholesterol (> 3.0 mmol/L). After 16 weeks, only 23.5 % in group 1 and 7.1 % in group 3 exceeded the reference value for total cholesterol, while all subjects of group 2 exhibited normal values. 35.3 % in group 1, 5.9 % in group 2 and 28.6 % in group 3 remained high LDL cholesterol levels after the intervention. HDL cholesterol decreased in group 1 and 2 after 12 weeks (both *p* ≤ 0.03) and was only in group 2 significantly lower after 16 weeks compared to baseline (*p* = 0.02). HDL cholesterol levels of group 3 and the control remained unchanged throughout the study. Changes from baseline in HDL cholesterol after both the reduction and stabilization phases were comparable across all groups. Non-HDL-cholesterol concentrations decreased significantly within group 2 and 3 (*p* < 0.01), while the reduction in group 1 was not significant. The change from baseline did not differ between the four groups. Triacylglyceride (TAG) levels decreased significantly by −17.7 % within group 1 after 16 weeks (*p* < 0.05), but this reduction was not statistically significant compared to the other groups. Initially, TAG concentrations were elevated (> 1.7 mmol/L) in 29.4 %, 11.8 % and 14.3 % of the subjects in group 1, 2 and 3, respectively. After the intervention, none of the subjects in group 1 and only 5.9 % of the subjects in group 2 exhibited elevated TAG concentrations, while the proportion in group 3 remained unchanged. Absolute values of total, HDL, and LDL cholesterol remained comparable between the groups at all measured time points (Table 3).

Fasting glucose decreased by −3.6 % in group 1 after 16 weeks (*p* = 0.03). The change from baseline was greater than in the control group (*p* = 0.002), which recorded a minimal increase (2.7 %) (Figure 5A). Fasting insulin and HOMA-IR decreased significantly in group 1 and 2 after12 (both *p* < 0.05) and 16 weeks (*p* < 0.05), whereas group 3 and the control group showed no meaningful changes. Notably, group 3 displayed the largest reduction in insulin levels after 12 weeks (−44.0 %), but this difference was not statistically significant due to the high interquartile range (IQR). After the second stabilization phase, group 1 exhibited a significantly greater reduction in insulin and HOMA-IR compared to the other groups (both *p* < 0.05) (Figure 5B). HbA1c showed minor, non-significant fluctuations in all groups and remained stable compared to baseline after 12 and 16 weeks. Absolute values for all glucose metabolism parameters were comparable across groups at 12 and 16 weeks, respectively (Table 3).

**FIGURE 5:**
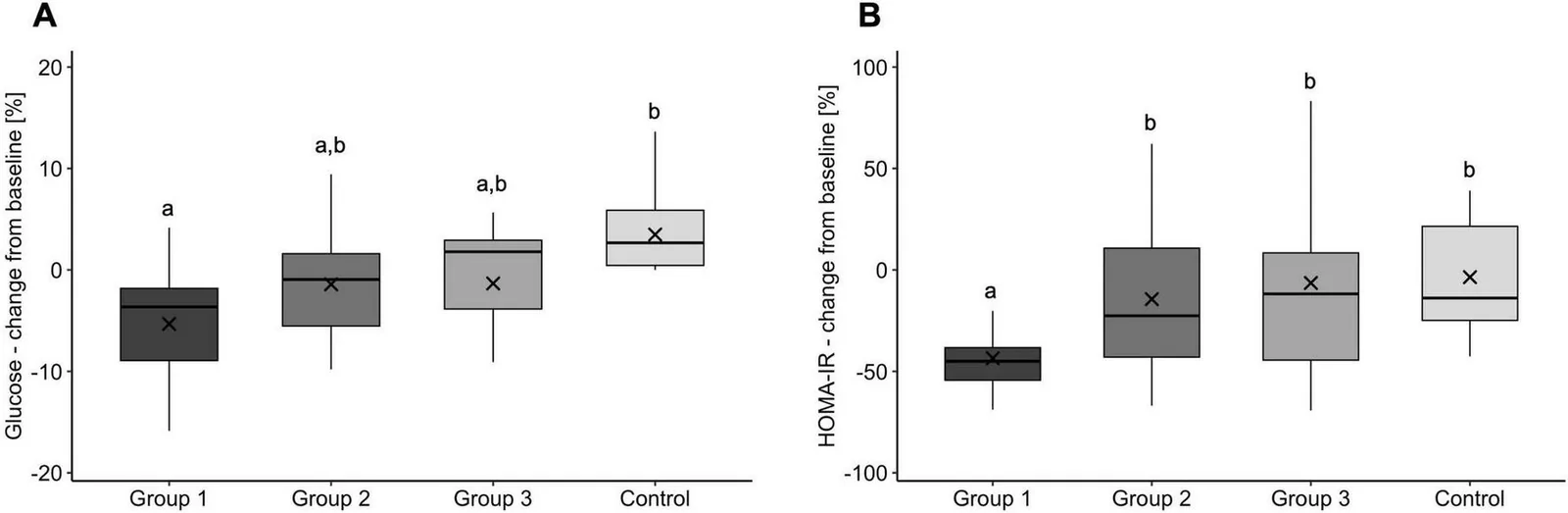
Comparison of percentual change from baseline of **(A)** glucose and **(B)** HOMA-IR after 16-weeks. The results are shown as box plot with median (indicated as line) and mean (indicated as cross). Groups that do not share indices (a,b) differ significantly [one-factor ANOVA or Kruskal-Wallis test depending on normal distribution followed by Benjamini-Hochberg correction (*p* < 0.05)].

### Fatty acid composition in erythrocytes during the intervention

Total SFA levels remained mostly stable in groups 1, 3, and the control throughout the study, with no significant changes compared to baseline. In group 2, an increase of 3.6 % was observed after 16 weeks (*p* < 0.01), mostly driven by a slight rise in palmitic acid (C16:0) of 6.0 % after 12 weeks and 5.3 % after 16 weeks (both *p* < 0.01). However, at the end of the intervention, group 3 had higher palmitic acid concentrations compared to group 1 (*p* = 0.03) and the change from baseline of group 2, 3 and 4 was significantly higher compared to group 1 (*p <* 0.01). Furthermore, myristic acid (C14:0) decreased significantly in group 1 (*p* < 0.01), while heptadecanoic acid (C17:0) increased in group 1 and 2 (both *p* ≤ 0.04) after 16 weeks. For both fatty acids, the change from baseline did not differ between the four groups (Supplementary Table 2).

Overall, MUFA concentrations, including oleic acid (C18:1*n*-9) and palmitoleic acid (C16:1*n*-7), showed no significant changes during the study, except in group 4. In this group, the sum of MUFA increased slightly, but significantly toward the end of the study (*p* = 0.03), with no significant change from baseline between groups (Supplementary Table 2).

Total PUFA, as well as *n*-6 PUFA, remained relatively stable across all groups. GLA, dihomo-γ-linoleic acid and adrenic acid concentrations decreased significantly in group 1,2 and 3 throughout the study (*p* < 0.01). For GLA, no group difference emerged across the five time points, but the change from baseline of group 2 was significantly higher compared to group 3 and 4 (*p* ≤ 0.04). Regarding dihomo γ-linoleic acid, group 3 and 4 exhibited higher concentrations after 16 weeks compared to group 2 (*p* ≤ 0.04), which showed the strongest change from baseline (−14.0 %). For adrenic acid the strongest decline was evident in group 3 (−9.5 %), which showed a significant difference in the change from baseline compared to group 1 and 4 (*p* ≤ 0.04) (Supplementary Table 2).

ALA concentrations increased significantly in all intervention groups, with changes of 41.7–101.0 % after 12 weeks (all *p* < 0.01) and 52.7–79.4 % after 16 weeks (all *p* ≤ 0.01). Therefore, changes from baseline after 16 weeks were higher in the three intervention groups compared to control group (all *p* ≤ 0.03) (Figure 6A) and at the end of the intervention all groups exhibited higher ALA concentrations compared to the control group (all *p* ≤ 0.03). EPA concentrations decreased by −13.9 % in group 1 after 12 weeks (*p* = 0.02) but returned to baseline after the stabilization phase. No group differences emerged for absolute amounts or the change from baseline. DPA and DHA fluctuated across the groups without significant within and between group differences, while the control group showed the greatest non-significant decrease after 16 weeks for both fatty acids. Consequently, the sum of *n*-3 PUFA, *n*-3 LCPUFA as well as the *n*-3 Index did not change significantly over the course of the study in any of the four groups, but comparing the change from baseline after 16 weeks, group 4 showed a small but significant decrease (−9.0 to −6.0%) compared to group 1 (1.7 to 5.2 %) and 2 (1.7 to 2.0 %; all *p* < 0.03) (Figure 6B).

**FIGURE 6:**
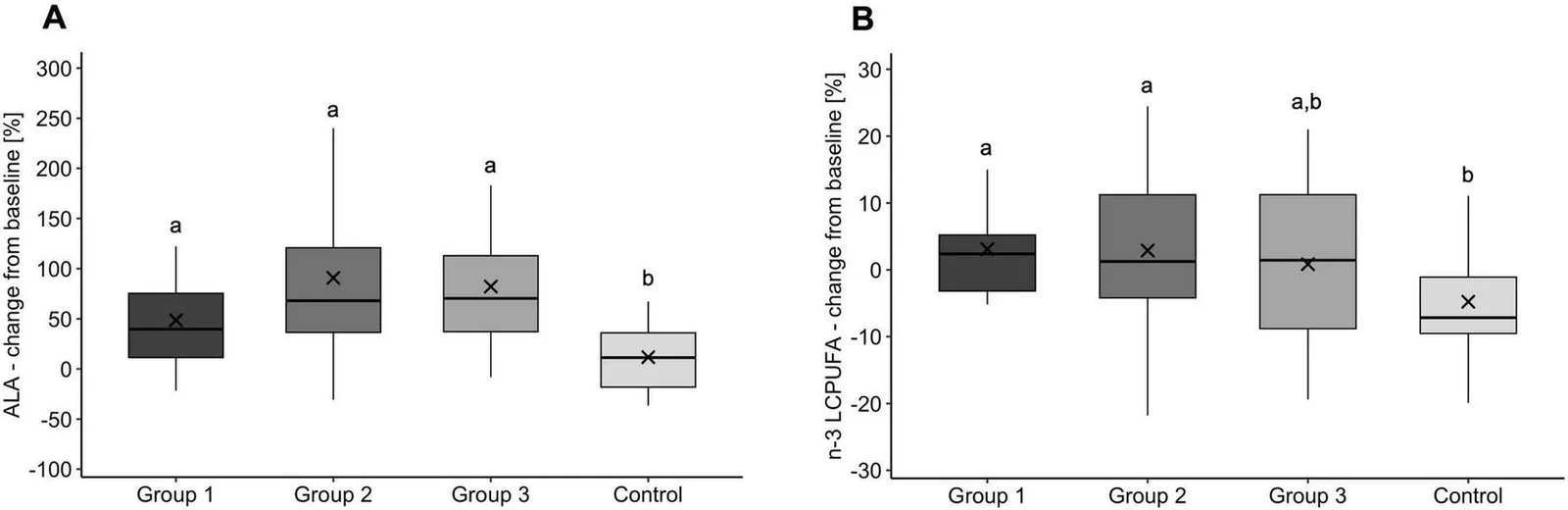
Comparison of percentual change from baseline of **(A)** α-linolenic acid (ALA) and **(B)** long chain *n*-3 polyunsaturated fatty acids (*n*-3 LC PUFA) after 16-weeks. The results are shown as box plot with median (indicated as line) and mean (indicated as cross). Groups that do not share indices (a,b) differ significantly [one-factor ANOVA or Kruskal-Wallis test depending on normal distribution followed by Benjamini-Hochberg correction (*p* < 0.05)].

The %*n*-6 and %*n*-3 in HUFA did also not change significantly over the course of the study in any of the four groups, but the change from baseline in the control group was slightly, but significantly higher for %*n*-6 and lower for %*n*-3 in HUFA (both, *p* = 0.04). Regarding different fatty acid ratios (*n*-6/*n*-3, ARA/EPA, ARA/DHA) no significant within and between group differences emerged after 16 weeks. In addition, D9D (C18:1*n*-9/C18:0) did not differ within and between the groups, but *n*-6 D5D (C20:4*n*-6/20:3*n*-6) significantly increased in group 1, 2 and 3 throughout the study (10.0 –13.4 %; all *p*< 0.05). In contrast both indices for D6D decreased after 16 weeks in group 1 and 2 (C20:3*n*-6/C18:2*n*-6) as well as group 3 (C20:5*n*-3/C18:3*n*-3), respectively (all *p* < 0.05). For *n*-6 D5D the highest decline was shown in group 2 (−12.0 %) compared to group 4 (−3.1 %) (*p* < 0.01), while the change from baseline for *n*-3 D5D was significantly higher in all three intervention groups (−28.6 to −46.3 %) compared to the control group (−11.9 %; all *p* < 0.01) (Supplementary Table 2).

To evaluate the conversion rate of ALA into its LC metabolites, in the three groups that followed a dietary intervention, we formed subgroups to examine sex (men vs. women), BMI (< 31 vs. ≥ 31), age (< 41 vs. ≥ 41), LA status (< 10.7 vs. ≥ 10.7 % FAME), ARA status (< 15.1 vs. ≥ 15.1 % FAME) and EPA status at baseline (< 0.7 % FAME vs. ≥ 0.7 % FAME) as potential influencing factors. No difference emerged for the change from baseline of ARA, adrenic acid, osbond acid, EPA, DPA, DHA or *n-*3 LCPUFA regarding sex, age, LA and ARA status between subjects with high or low values at the start of the intervention. However, subjects starting with an BMI < 31 showed a higher increase in EPA (16.8 % vs. −2.1 %), DPA (7.9 % vs. −1.6 %), DHA (4.4 % vs. −1.9 %) as well as *n*-3 LCPUFA (5.5 %vs. −0.6 %; all *p* ≤ 0.02). Furthermore, participants with lower baseline EPA status showed a higher increase in EPA (11.9 % vs. 1.3 %), DHA (6.4 % vs. −1.5 %) and accordingly *n*-3 LCPUFA concentrations (7.7 % vs. 0.5 %) compared to those with a higher status at the beginning (all *p* < 0.03) (Supplementary Table 3).

### Micronutrient status during the intervention

To prevent nutrient deficiencies due to the calorie-restricted diet, nutrient status was continuously monitored throughout the study. Concentrations of folic acid and holo-transcobalamin remained stable in all groups, with no significant differences between groups at any time point. Vitamin D_3_ concentrations increased in all groups by 39.1–45.8 % at week 12 (all *p* < 0.01) and 30.4–48.5 % at week 16 compared to baseline (all *p* < 0.01). The changes were consistent across all groups, with no significant intergroup differences. TSH concentrations remained stable in all groups during the intervention. However, fT_3_ and fT_4_ levels significantly decreased in all intervention groups (all *p* < 0.001) and the control group (both *p* < 0.001) over the course of the study. At week 12, the change from baseline for fT_3_ was greater in group 3 and the control compared to groups 1 and 2 (all *p* ≤ 0.03). However, no between group differences were observed at any time point. Ferritin concentrations decreased in all groups over the course of the study (all *p* < 0.001). After 16 weeks, concentrations were lowest and dropped −20.8 to −40.4 %, but the changes from baseline, as well as total ferritin concentrations were comparable across all groups. The excretion of electrolytes was only measured at baseline and after 16 weeks. Magnesium excretion in 24-h urine samples did not change after the intervention. Sodium excretion, however, decreased in group 1 by −14.2 % (*p* = 0.04), while it increased in the control group by 28.3 % (*p* < 0.01) compared to baseline. Overall, the control group exhibited a higher change from baseline compared to group 1 and 3 (both *p* ≤ 0.03) and exhibited the highest total sodium excretion after 16 weeks which was significant compared to group 1 (*p* < 0.05) (Supplementary Table 4).

## Discussion

The PreVa study evaluated the effectiveness of a gradual energy reduction using individualized daily menu plans within different levels of counseling intensity. The primary goal of the intervention was to achieve a clinically relevant weight reduction of at least 5 % of baseline weight and further to investigate potential improvements in cardiometabolic risk factors. All intervention groups achieved a more pronounced weight loss compared to the control group, with 53.3–78.9 % of participants in the intervention groups reducing their body weight by ≥ 5 % after 16 weeks. BMI and body composition improved in all four groups, but the effects were more pronounced in the intervention groups, with a significantly greater change from baseline in BMI, waist circumference and body fat compared to the control group. Overall, participants shifted from obesity (class 1) to overweight classification.

### Comparison with app-based weight loss interventions

The central objective of this study was to assess the efficacy of an app-based intervention compared with therapy based on personal counselling. Application of digital tools has the potential to enhance adherence by providing continous support and allowing flexible use tailored to participants’ needs and schedules. As a special feature compared to many previous app-based weight loss programs, the PreVa study incorporated menu plans as a core component, which makes a direct comparison difficult. However previous research has demonstrated that purely app-based interventions can induce clinically significant weight loss, depending on program design, digital tools and adherence levels. In contrast, self-monitoring apps alone, without regular visits or educational content, do not appear to be as effective for weight loss (18). However, Toro-Ramos et al. evaluated the effectiveness of a smartphone-based approach for behavioral and nutritional therapy and showed a comparable weight loss to our data (−7.5 %) after 15 weeks, accompanied by reductions in total body fat (−6.0 %) and visceral fat (−3.4 kg) (19). Furthermore Roth et al. (12 weeks) demonstrated that a purely app-based multimodal program resulted in a clinically relevant weight reduction (−7.8 %) and improved waist-to-height ratio in a German cohort after 12 weeks (20). In addition Gemesi et al. found in their evaluation of an app-based multimodal lifestyle intervention on body weight in persons with obesity a moderate weight loss of 3.2 kg after 12 weeks, suggesting that effectiveness may depend on program engagement and individualized adherence (21). Other studies suggest that additional personalized counseling, as implemented in the PreVa study, enhances adherence, our findings are therefore consistent with findings showing that a combination of app usage and personal counseling was more effective than app-based self-monitoring alone (22). In comparison with our results, studies comparing traditional, digital and hybrid behavioral interventions for weight loss have shown that although all groups achieved clinically relevant weight reduction (> 5 %) after 6 months, traditional in-person dietary counseling was the most effective (−8 %) (23). However, Thomas et al. observed comparable weight loss outcomes between a smartphone-based behavioral intervention and an intensive group therapy program after 18 months, suggesting that the intervention modality itself may not be the primary determinant of success (24). In summary, these findings support the hypothesis that digital health applications can complement traditional nutritional counselling, and in line with our data, suggest that a certain degree of personal support appears to be necessary. In the PreVa study, the lack of additional app-based features, such as health feedback or interactive nutritional counseling, may explain why the app did not provide an additional benefit for weight loss. While participants had continuous access to menu plans and weight-tracking functions, educational content and individual feedback were provided during in-person study visits. Given the relatively short intervention duration (16 weeks), follow-up studies are needed to determine whether long-term app usage could further enhance adherence and sustain weight loss, as the PreVa concept was designed to be repeated as needed.

### Effect of gradual energy restriction on weight and body composition

As the three intervention groups did not differ in any parameters over the course of the study, the structured nutrient-optimized menu plans were likely the primary determinant of weight loss and body composition improvements. The app-based publication of menu plans is therefore a suitable way of distributing the concept across the target group who can implement it in the long term.

Notably, participants also lost weight during the maintenance phases, despite receiving isocaloric diets. A higher energy intake than required prior to the start of the PreVA study is likely one of the main reasons for being overweight. Therefore, the maintenance phases lead to passive energy restriction, resulting in weight loss. In addition, the dietary concept encompassed a transition to a more plant-based diet. It has been documented that these dietary patterns are more effective for weight loss compared with to omnivorous approaches (25–27). A significant factor contributing to this observation is the increased consumption of fiber-rich plant-based foods, resulting in a higher dietary volume and delay of gastric emptying. A higher fiber intake therefore decreases hunger, promotes a sense of fullness and satiety and hence, plays a role in the control of energy balance (28).

The gradual energy reduction, incorporating maintenance phases, was planned to reduce the adaptive thermogenesis or metabolic adaptation of the organism to calorie restriction (29). Therefore, the plans were designed to minimize the reduction in basal metabolic rate that occurs with continuous calorie restriction, to prevent weight regain (30). Despite a small but significant reduction in basal metabolic rate, the loss of muscle mass across all intervention groups was small, with most of the weight loss attributable to fat mass reduction. Especially the decrease in visceral fat is a critical factor in improving metabolic health, especially with regard to insulin resistance (31, 32). The intervention resulted in a reduction of waist circumference, a well-established marker of visceral adiposity (33), almost twice as high compared to the control group. However, many participants remained above sex-specific reference values (< 102 cm for men, < 88 cm for women). A high proportion of abdominal fat is associated with systemic inflammation, which is reflected in elevated hsCRP concentrations (> 0.3 mg/L) (34). Although a reduction in hsCRP concentrations was observed in all groups, these changes did not reach statistical significance.

### Improvement of cardiovascular risk factors and erythrocyte fatty acid profile

Further cardiovascular risk factors such as blood lipids, hypertension, or impaired glucose tolerance belong to the secondary endpoints of the PreVA study. The study included subjects without known or diagnosed hypertension, dyslipidaemia, or impaired glucose tolerance. However, some of the participants displayed slightly elevated levels at baseline and overall an improvement in the cardiovascular risk markers was achieved during the intervention, with no significant differences between the three intervention groups. The improvements in the intervention groups differed significantly from the control group. The weight loss could be one of the reasons for the improvements in blood pressure, blood lipids and glucose metabolism (35, 36). However, we assume the observed reduction in cardiovascular risk factors occurred due to the holistic pattern of the intervention (weight loss plus optimized nutrient intake), which has synergistic adaptive effects. Regarding blood pressure, the menu plans resembled the Dietary Approaches to Stop Hypertension (DASH) diet, which is characterized by reduced sodium and sugar intake and increased consumption of fruits, vegetables, and fiber, contributing to blood pressure regulation (37).

The modification of carbohydrate quality in the menu plans, including a reduction in refined grains and added sugars while increasing fiber intake, may have contributed to improvements in insulin sensitivity (38, 39). However, only groups 1 and 2 showed a significant improvement in fasting insulin and insulin sensitivity (HOMA-IR). These results may be linked to the more intensive dietary advice and recommendations on physical activity provided to both groups. Physical exercise is recommended and known to improve glucose metabolism (40, 41). Originally, smartwatch integration was planned to enable closer monitoring of the activity in group 1 and to potentially reinforce app-based weight loss and related improvements. However, technical issues with smartwatch connectivity prevented its successful implementation.

The substitution of SFA with MUFA and PUFA, likely improved blood lipids, as this switch enhances LDL clearance (42, 43) contributing to the reduction in total and LDL cholesterol levels. We also observed a reduction in HDL cholesterol in group 2, which was unexpected, as weight loss and improved dietary fat quality, are generally expected to increase or maintain HDL cholesterol concentrations (44, 45). This decrease may reflect short-term effects of caloric restriction and reduced total fat intake. However, the clinical relevance of HDL cholesterol changes is increasingly questioned, since large cohort studies and meta-analyses indicate that raising HDL cholesterol does not provide substantial cardiovascular benefit independent of lowering LDL or non-HDL cholesterol (46, 47). Hence the observed reduction of HDL cholesterol does not offset the beneficial reduction in atherogenic lipoproteins and therefore more favorable cardiovascular risk profile. Besides the change in dietary fat quality, the increased fiber intake facilitates reduction in plasma cholesterol reduction likely due to increased excretion and reduced reabsorption of bile acids. Bile acids require cholesterol for replenishment, resulting in a decrease in blood levels (39, 48, 49). Notably, the changes in SFA (< 7 EN%) and MUFA (> 10 EN%) intake over the 16 weeks were not reflected in the fatty acid profiles of the erythrocytes. This could be explained by the fact that SFA and MUFA can also be synthesized endogenously, which could mask their intake through diet (45). Therefore, fatty acid profiles can reliable serve only as a compliance marker for PUFA intake.

Regarding fatty acids, an increased intake of *n*-3 PUFA was further emphasized in the menu plans. Especially EPA and DHA have various benefits for CVD including reduced plasma triglyceride concentration, size of lipoproteins and inflammation. In addition, improvements in plaque stability, vascular function and arrhythmias have been observed (50). Furthermore, the *n*-3 Index, which comprises the sum of EPA and DHA in erythrocyte lipids, is used as a risk factor for death from coronary heart disease with an *n*-3 Index of ≤ 4 % being associated with the lowest and an index of ≥ 8 % being associated with highest cardioprotective effect (51).

The increase in *n*-3 PUFA, was induced by the daily intake of approximately 5–10 ml flaxseed oil (∼3 g ALA/day). This led to a significant increase in the ALA concentration in the erythrocytes in the intervention groups reflecting the compliance with the menu plans, as the effect was particularly evident in comparison to the control group. Previous studies have already shown that an increased ALA intake via flaxseed oil leads to a significant increase in the erythrocyte membrane (52, 53). ALA can be converted to EPA, DPA and DHA in the organism via several desaturase and elongase steps and although the conversion rate is limited (EPA: 8 %, DHA: 0.02–4 %) (54), an increased intake may contribute to an improvement of the *n*-3 LCPUFA status (55). In the PreVa subjects there were no significant changes in EPA, DPA and DHA concentrations when comparing all groups. Egert et al. found that an energy-restricted diet enriched with 3.7 g of ALA from rapeseed oil led to significant reductions in body weight, systolic blood pressure, dyslipidemia, insulin resistance, inflammation, and endothelial dysfunction after 26 weeks, but did not systematically increase ALA or EPA in plasma lipids of overweight and obese subjects with metabolic syndrome (56). Furthermore, DHA decreased in both the ALA-enriched and control diets, which we did not observe in the PreVa study. Moreover, subjects with lower baseline EPA status showed a significant increase in EPA (+ 11.9 % vs. + 1.3 %), DHA (+ 6.4 % vs. -1.5 %), and consequently in total *n*-3 LCPUFA concentrations (+ 7.7 % vs. + 0.5 %) after 16 weeks compared to those with higher initial values. These findings are consistent with previous results from our research group, although the magnitude of improvement in the PrevA study was substantially smaller than in the KoALA and NuEva studies, where EPA increased by 63–79 % (52, 57).

One possible explanation for the lack of a systematic increase in *n*-3 LCPUFA in the PreVa study could be the higher BMI of the participants. It has been shown that obesity alters the PUFA profile, independent of age, with most studies reporting lower *n*-3 LCPUFA concentrations in plasma or erythrocyte lipids compared to healthy controls (58–61). In our analysis, subjects with a baseline BMI < 31 (*n* = 25) exhibited greater increases in EPA (+ 16.8 % vs. −2.1 %, *p* < 0.01), DPA (+ 7.9 % vs. −1.6 %, *p* < 0.001), DHA (+ 4.4 % vs. −1.9 %, *p* < 0.01), and total *n*-3 LCPUFA (+ 5.5 % vs. −0.6 %, *p* < 0.001) compared to the subjects with a baseline BMI ≥ 31 (*n* = 23), although these participants were still classified as overweight or obese.

Therefore a modified PUFA metabolism due to obesity would be conceivable and elevated D6D activity and reduced D5D activity in plasma phospholipids or serum cholesteryl esters have been observed in obese individuals (62, 63), reflecting a fatty acid pattern similar to that seen in insulin-resistant subjects (64). This is primarily attributed to hyperinsulinemia, which induces sterol regulatory element-binding protein 1c and thereby increases D6D expression and activity (65). Over the course of the PrevA study, D6D activity decreased and D5D activity increased, in parallel with improvements in fasting glucose and HOMA-IR, yet no significant enhancement of *n*-3 LCPUFA status was achieved.

The sustained energy deficit throughout the study may also have played a role, as the efficiency of ALA conversion to EPA might be influenced by energy availability. Egert et al. suggested, conversion efficiency depends not only on ALA intake but also on total energy intake, with a catabolic state limiting the availability of ALA for conversion (56). ALA serves not only as a precursor for EPA, DPA and DHA but also as a substrate for β-oxidation, particularly under conditions of negative energy balance (66). Thus, although the ALA supply was reflected in an increase in erythrocyte lipids, the 16-week intervention period may have been too short to enhance endogenous conversion while being in a caloric deficit.

Finally, the menu plans included did not limit the intake of fish, resulting in approximately 500 mg/d of EPA, DPA and DHA, when the menu plans were consumed as recommended. This direct supply of EPA, DPA and DHA could have exerted a stabilizing, rather than increasing effect on *n*-3 LCPUFA status, implying that higher intakes would have been required to achieve a measurable increase in *n*-3 LCPUFA status.

### Micronutrient status

The menu plans ensured an adequate intake of vitamins, minerals and trace elements according to the subject’s requirements following the reference values for nutrient intake of the DGE. Ensuring an adequate intake of micronutrients despite energy restriction is a key consideration in weight loss interventions (67). In the PreVa study, no major declines were observed, suggesting that the subjects had an adequate nutrients status at baseline and the menu plans further provided sufficient micronutrient intake through intervention phase. In group 1 a decline in sodium excretion was observed, which is most likely attributable to a reduction in salt consumption which was achieved via menu plans. In addition, a slight decrease in ferritin and vitamin B_12_ concentrations were noted, likely due to reduced meat consumption, as both nutrients are contained in higher amounts in these foods (68, 69). These markers may be considered indicators of dietary adherence, reflecting a shift toward a more plant-based diet. Given that the study cohort was predominantly female, especially the observed reduction in ferritin seems to be a disadvantage from a health perspective. Ferritin is also an acute-phase protein, and previous studies have shown positive associations between ferritin concentrations and BMI, waist circumference, and body fat percentage (70, 71). Therefore, the decline in ferritin may also partly reflect improvements in body weight and body composition rather than a clinically relevant depletion of iron stores. Importantly, baseline ferritin concentrations were in the upper reference range and remained within the desired range after the intervention. In addition, transferrin values did not indicate an adverse change in iron metabolism. Thus, despite the reduction in ferritin, the dietary intervention does not appear to have compromised iron status in the PreVa subjects. Furthermore, thyroid hormones decreased significantly in all four groups. A systematic review suggest that dietary calorie restriction can lead to a reduction in fT_3_ and TSH levels, whereas fT_4_ tends to increase in overweight individuals (72). Seasonal and biological variations due to pulsatile secretion, sleep, stress and current energy availability are another potential explanation that could explain why a reduction may also have occurred in the control group. Overall, thyroid levels remained within the reference range in all four groups, which is why no adverse health effects are assumed.

### Strengths and limitations

The PreVa study has several strengths. First, the energy restriction was introduced gradually with maintenance phases, which facilitated better adaptation to the energy deficit. Second, the participants could choose from a variety of menu plans ensuring an optimized intake of energy, as well as macro- and micronutrients. The menu plans contained precise instructions on ingredients and preparation and were handed along with the provision of study foods such as flaxseed oil. Third, during the 16-week intervention, blood samples were taken every 4 weeks to examine changes in parameters over time. Regular study visits also served to maintain compliance and, depending on the assigned group, to maintain motivation through feedback. Fourth, the frequency of study appointments and the analysis of a wide range of parameters, allowed a comprehensive view of the effects of the interventions over the course of time. However, given the nature of the intervention participants and study personnel were aware of group allocation, which is why performance and detection bias may have occurred. Regarding the adherence of the dietary intervention, motivation, engagement, or reporting behavior could have vary between groups. Especially the implementation of the menu plans in everyday life depended heavily on the motivation of the participants, as these are very time-consuming. Compliance was measured using self-reported information in combination with an analysis of nutritional status, which can also be influenced by various factors. Physical activity, which can contribute significantly to calorie deficit, was not systematically recorded, which must be considered a limitation. For group 1, it was planned to connect the app to a smartwatch specially provided for the study in order to record physical activity levels and further increase motivation of the participants. However, due to technical difficulties, this feature could not be implemented.

## Conclusion

The majority of participants in the intervention groups achieved clinically relevant weight reduction after 16 weeks, which was significantly greater compared to the control group. The app-based concept provided comparable benefits but did not yield additional advantages, suggesting that this approach achieves weight loss similar to (personal) nutritional counselling. It should be noted, however, that the sample size may not have been large enough to reveal differences between the effects of the three interventions on weight reduction and further cardiovascular risk factors. All three interventions improved cardiovascular risk factors, particularly reductions in blood pressure, LDL cholesterol, and markers of glucose metabolism. These effects are likely attributable to the individualized menu plans that combine gradual energy restriction and optimized nutrient intake resulting in a synergistic adaptive effect. As response to a moderate flaxseed oil supplementation, ALA concentrations in erythrocyte lipids increased during the intervention, but no improvement in *n*-3 LCPUFA status was achieved.

As part of the PrevA study, the dietary program—based on individualized meal plans—was evaluated for its effectiveness in promoting weight loss, reducing body fat percentage, and improving LDL cholesterol levels in participants with a BMI of ≥ 25 to < 35 kg/m^2^ (overweight or class 1 obesity). The practical approach is suitable for long-term implementation in daily life and leads to a reduction in the aforementioned cardiovascular risk factors.

## Data Availability

The original contributions presented in this study are included in this article/Supplementary material, further inquiries can be directed to the corresponding author.
