# Supplementary material for: A randomized parallel-designed study evaluating the effect of an app-based nutritional concept for weight reduction and further cardiovascular risk factors: Insights from the PreVA study
Source: Front Nutr. 2026 Sep 23;13:1815820. doi: 10.3389/fnut.2026.1815820 (PMC13644689; doi:10.3389/fnut.2026.1815820)
Supplement: Supplementary file 1 [file Data_Sheet_1.pdf]

Supplementary Material

Supplementary Figures and Tables

Table S1: Nutrient intake of study subjects in each group before baseline assessment (full self-reports, 5 days)

**Nutrient**	**Group 1** (*n*=18)		**Group 2** (*n*=19)		**Group 3** (*n*=15)		**Control** (*n*=16)	
Energy [kcal]<br>Men (2100–3100)[1]<br>Women (1700–2500)[1]	2130.39 (1983.43, 2293.74)<br>1576.75–10226.89	a	2240.96 (1924.07, 2897.87)<br>1570.44–20294.98	a	2049.01 (1685.85, 2534.14)<br>1089.90–3115.38	a	1988.34 (1657.24, 2559.72)<br>1249.12–3551.52	a
Carbohydrate [g/d]<br>> 50 en%[1]	225.60 (178.48, 253.63)<br>130.78–480.25	a	221.20 (182.82, 301.77)<br>145.81–2398.26	a	220.24 (161.96, 303.59)<br>141.95–379.25	a	206.65 (174.32, 258.81)<br>119.45–339.06	a
Total dietary fiber [g/d]<br>30 g/d[1]	21.45 (16.76, 27.50)<br>10.55–177.13	a	22.40 (19.26, 26.98)<br>6.38–152.32	a	20.90 (16.04, 25.56)<br>9.82–43.85	a	23.23 (18.18, 28.27)<br>11.09–36.21	a
Total dietary sugar [g/d]	101.19 (76.83, 125.58)<br>51.47–233.76	a	100.68 (78.45, 136.31)<br>46.40–397.03	a	103.42 (78.53, 142.22)<br>53.61–235.15	a	105.53 (92.75, 117.08)<br>58.90–166.68	a
Glucose [g/d]	16.50 (12.78, 21.10)<br>6.64–28.69	a	17.48 (13.39, 20.39)<br>8.97–47.75	a	19.00 (12.68, 28.11)<br>6.80–37.09	a	20.09 (16.75, 26.13)<br>8.20–39.25	a
Fructose [g/d]	19.13 (14.90, 28.51)<br>9.44–39.01	a	18.32 (14.69, 25.50)<br>5.34–47.37	a	27.47 (14.71, 34.36)<br>6.61–42.64	a	25.51 (19.62, 31.56)<br>7.11–40.05	a
Alcohol [g/d]	7.71 (0.10, 16.68)<br>0.00–23.68	a	4.77 (0.07, 15.43)<br>0.00–33.81	a	1.77 (0.23, 5.01)<br>0.00–10.67	a	2.05 (0.05, 10.76)<br>0.02–22.20	a
Protein [g/d]<br>0.8 g/kg body weight[1]	82.63 (72.20, 93.33)<br>49.93–456.03	a	85.33 (69.55, 116.10)<br>48.28–1093.31	a	79.54 (66.80, 100.77)<br>33.31–134.68	a	84.46 (71.55, 107.69)<br>44.87–153.75	a
Fat [g/d]<br>Approx. 30 en%[1]	86.74 (77.84, 101.02)<br>63.38–686.40	a,b	97.93 (79.91, 123.36)<br>63.50–1180.85	a	77.59 (59.85, 100.30)<br>37.93–132.56	b	82.72 (65.22, 115.07)<br>43.25–187.27	a,b
SFA [g/d]<br>< 10 en%[1]	39.57 (31.95, 47.12)<br>24.81–164.85	a	45.16 (35.09, 55.55)<br>28.18–474.12	a	32.90 (26.33, 42.89)<br>17.64–61.78	a	38.87 (26.42, 54.07)<br>18.01–82.16	a
MUFA [g/d]<br>≥ 10 en%[1]	29.02 (26.96, 37.17)<br>20.87–305.96	a	36.00 (28.35, 41.71)<br>21.82–514.65	a	24.01 (19.38, 34.23)<br>13.33–45.78	a	29.25 (22.59, 40.98)<br>16.13–70.62	a
PUFA [g/d]<br>≥ 10 en%[1]	12.03 (9.22, 14.07)<br>6.09–182.95	a	13.26 (8.18, 20.11)<br>4.74–106.37	a	9.87 (6.76, 12.73)<br>4.66–22.76	a	11.19 (8.41, 17.29)<br>4.86–23.01	a

Cholesterol [mg/d]<br>< 300 mg[1]	418.44 (311.62, 494.26)<br>196.78–617.20	a	351.69 (262.53, 450.59)<br>121.01–4469.09	a	251.26 (173.11, 478.49)<br>98.11–635.81	a	425.44 (364.35, 464.28)<br>207.36–791.13	a
LA [g/d]<br>2.5 en%[1]	9.90 (7.04, 11.52)<br>4.51–174.49	a	9.69 (6.57, 16.88)<br>3.93–94.29	a	8.37 (5.63, 10.57)<br>3.92–16.93	a	9.03 (6.92, 13.04)<br>3.64–18.80	a
ARA [g/d]	0.19 (0.17, 0.25)<br>0.05–0.43	a	0.20 (0.15, 0.30)<br>0.03–11.30	a	0.13 (0.08, 0.29)<br>0.02–0.62	a	0.23 (0.16, 0.33)<br>0.09–0.78	a
ALA [g/d]<br>0.5 en%[1]	1.14 (0.95, 1.70)<br>0.75–7.49	a	1.57 (1.08, 2.25)<br>0.58–19.06	a	1.19 (0.87, 1.43)<br>0.44–4.09	a	1.39 (0.91, 2.33)<br>0.56–3.11	a
EPA [g/d]	0.11 (0.06, 0.23)<br>0.01–0.85	a	0.10 (0.03, 0.22)<br>0.01–1.75	a,b	0.02 (0.01, 0.05)<br>0.01–0.47	b	0.06 (0.02, 0.32)<br>0.01–0.59	a,b
DPA [g/d]	0.04 (0.02, 0.10)<br>0.01–0.21	a	0.04 (0.01, 0.11)<br>0.00–0.42	a	0.01 (0.00, 0.07)<br>0.00–0.34	a	0.04 (0.02, 0.14)<br>0.00–0.22	a
DHA [g/d]	0.18 (0.14, 0.39)<br>0.02–0.88	a	0.19 (0.09, 0.30)<br>0.01–2.83	a	0.08 (0.03, 0.29)<br>0.00–1.03	a	0.23 (0.13, 0.48)<br>0.05–1.10	a
Vitamin A [mg/d]<br>0.7–0.85 mg/d[1]	0.49 (0.41, 1.09)<br>0.24–18.93	a	0.53 (0.36, 1.08)<br>0.28–2.45	a	0.41 (0.30, 0.55)<br>0.18–1.24	a	0.55 (0.42, 0.66)<br>0.26–1.58	a
Vitamin D [µg/d]<br>20 µg/d[1]	2.83 (1.85, 3.75)<br>1.19–15.97	a	2.59 (1.58, 3.77)<br>0.86–7.86	a	1.56 (1.17, 3.84)<br>0.66–10.19	a	2.85 (2.47, 3.86)<br>1.88–12.23	a
Vitamin E [mg/d]<br>8 mg/d[1]	9.36 (7.33, 12.69)<br>4.82–133.69	a	9.90 (7.89, 12.22)<br>5.23–95.69	a	8.09 (5.97, 11.94)<br>4.05–18.28	a	10.80 (8.20, 15.40)<br>5.05–20.02	a
Vitamin K [µg/d]<br>60–70 µg/d[1]	125.71 (71.18, 213.48)<br>48.39–584.43	a	128.12 (73.34, 225.88)<br>36.42–746.63	a	96.81 (49.44, 225.26)<br>37.87–386.00	a	165.24 (79.32, 249.60)<br>25.46–388.98	a
Thiamine [mg/d]<br>1.0–1.2 mg/d[1]	1.41 (1.02, 1.62)<br>0.80–12.54	a	1.41 (1.03, 2.04)<br>0.91–58.02	a	1.16 (0.90, 1.46)<br>0.73–2.04	a	1.24 (0.92, 1.71)<br>0.56–3.76	a
Vitamin B_2 [mg/d]<br>1.1–1.4 mg/d[1]	1.52 (1.25, 1.81)<br>0.85–4.29	a	1.63 (1.30, 2.03)<br>0.83–13.35	a	1.66 (1.12, 1.89)<br>0.50–2.34	a	1.64 (1.19, 1.94)<br>0.68–2.76	a
Niacin [mg/d]<br>12–15 mg/d[1]	36.75 (28.28, 40.15)<br>21.35–28.79	a	33.84 (27.60, 44.66)<br>19.87–49.73	a	30.06 (26.71, 40.09)<br>13.47–54.19	a	33.20 (29.79, 42.37)<br>18.71–69.15	a

Pantothenic acid [mg/d]<br>6 mg/d[1]	5.02 (4.21, 6.31)<br>3.48–41.24	a	5.22 (4.08, 5.95)<br>3.37–39.41	a	4.67 (2.77, 6.04)<br>1.26–7.89	a	4.98 (4.21, 6.70)<br>2.17–9.34	a
Vitamin B_6 [mg/d]<br>1.4–1.6 mg/d[1]	1.68 (1.42, 1.99)<br>1.06–7.84	a	1.85 (1.43, 2.19)<br>1.04–24.27	a	1.43 (0.95, 1.99)<br>0.67–2.74	a	1.57 (1.43, 2.26)<br>0.74–3.15	a
Biotin [μg/d]<br>40 μg/d[1]	53.32 (43.46, 61.49)<br>27.37–490.17	a	46.97 (39.86, 65.87)<br>31.52–326.14	a	52.62 (28.89, 61.44)<br>17.22–88.94	a	54.30 (49.14, 71.25)<br>26.84–92.22	a
Folic acid [μg/d]<br>300 μg/d[1]	274.44 (208.13, 345.65)<br>185.42–2416.75	a	248.77 (194.66, 348.59)<br>174.12–1895.70	a	241.88 (161.36, 345.85)<br>106.01–395.71	a	277.38 (204.80, 378.75)<br>126.13–417.84	a
Vitamin B_{12} [μg/d]<br>4 μg/d[1]	5.86 (4.43, 7.57)<br>1.21–18.18	a	5.97 (4.18, 8.88)<br>1.94–39.78	a	4.54 (2.29, 5.87)<br>1.35–13.30	a	5.00 (3.85, 6.58)<br>3.14–9.32	a
Vitamin C [mg/d]<br>95-110 mg/d[1]	122.56 (79.43, 163.92)<br>41.47–204.18	a	112.40 (76.39, 136.32)<br>23.13–572.31	a	83.21 (46.69, 116.61)<br>19.66–195.08	a	114.85 (96.80, 165.70)<br>16.46–227.00	a
Sodium [mg/d]<br>1500 mg/d[1]	2281.89 (1997.13, 2523.69)<br>950.94–4216.06	a	2636.03 (2261.24, 3969.79)<br>1114.25–37491.59	a	2251.00 (1601.73, 3148.51)<br>1405.17–7092.80	a	2358.27 (1578.68, 3287.67)<br>1145.56–4052.41	a
Potassium [mg/d]<br>4000 mg/d[1]	3163.17 (2356.31, 3733.38)<br>2189.80–12737.96	a	3180.45 (2608.68, 3945.57)<br>2139.79–27291.95	a	3030.10 (2481.55, 3559.50)<br>1489.26–4974.16	a	3463.43 (2752.61, 4044.26)<br>1539.77–5014.23	a
Magnesium [mg/d]<br>300–350 mg/d[1]	347.72 (281.77, 393.05)<br>224.76–2368.15	a	364.82 (304.50, 431.35)<br>206.15–2204.96	a	329.32 (271.46, 370.48)<br>143.80–458.02	a	366.24 (269.80, 401.43)<br>158.89–598.62	a
Calcium [mg/d]<br>1000 mg/d[1]	791.49 (677.01, 1065.23)<br>373.87–2397.47	a	886.89 (715.39, 1125.77)<br>387.70–5495.39	a	977.16 (735.95, 1368.15)<br>346.64–1503.28	a	923.02 (666.31, 1228.86)<br>496.16–1399.62	a
Iron [mg/d]<br>10–16 mg/d[1]	11.66 (10.15, 14.95)<br>7.70–35.86	a	12.71 (9.80, 14.96)<br>8.35–116.59	a	10.77 (9.63, 12.06)<br>4.73–14.85	a	11.05 (10.18, 14.96)<br>6.62–19.00	a
Iodine [μg/d]<br>180 μg/d[1]	102.76 (77.24, 126.88)<br>40.60–332.12	a	89.64 (68.27, 118.19)<br>55.03–871.30	a	82.40 (68.27, 119.37)<br>35.08–139.36	a	119.36 (92.65, 145.99)<br>45.05–229.76	a
Copper [mg/d]<br>1000–1500 μg/d[1]	1818.41 (1500.25, 2003.94)<br>1326.63–11443.57	a	1844.66 (1584.92, 2051.56)<br>796.70–7896.55	a	1775.67 (1345.24, 2132.79)<br>857.61–2942.79	a	1844.05 (1468.57, 2368.10)<br>1117.15–2981.35	a
Manganese [μg/d]<br>2000–5000 μg/d[1]	4281.52 (3499.34, 5461.68)<br>2100.81–23938.05	a	4673.77 (3447.51, 6255.78)<br>1145.57–23216.48	a	4203.61 (3088.69, 5717.72)<br>1682.74–7901.75	a	5072.89 (4262.72, 6724.41)<br>1895.33–11152.86	a

Zinc [mg/d]<br>7–16 mg/d[1]	11.06 (10.23, 13.37)<br>8.18–46.36	a	13.32 (9.11, 15.99)<br>7.32–214.99	a	10.47 (8.57, 12.22)<br>4.43–18.49	a	11.47 (8.56, 13.38)<br>6.11–18.22	a

[1] German Nutrition Society, Austrian Nutrition Society (eds.): Reference values for nutrient intake. Bonn, 3rd edition, 1st issue (2025)

Variable expressed as median (25th, 75th percentile); groups without a common letter are significantly different, $p < 0.05$; Abbreviations: SFA = saturated fatty acids, MUFA = monounsaturated fatty acids, PUFA = polyunsaturated fatty acids, ARA = arachidonic acid, EPA = eicosapentaenoic acid, DPA = docosapentaenoic acid DHA = docosahexaenoic acid, LA = linoleic acid, ALA = α-linolenic acid

Table S2: Fatty acid composition in erythrocytes during the intervention

	t	**Group 1** (*n*=18)	T[1]	G[2]	**Group 2** (*n*=19)	T	G	**Group 3** (*n*=15)	T	G	**Control** (*n*=16)	T	G
							**SFA**						
∑ SFA	0	35.50 (± 1.57)<br>35.20 (34.42, 35.92)	a	a	34.83 (± 1.20)<br>34.93 (34.06, 35.99)	a	a	35.52 (± 1.42)<br>35.23 (34.05, 36.65)	a	a	35.49 (± 1.33)<br>35.34 (34.54, 36.06)	a	a
	4	36.59 (± 3.05)<br>35.51 (35.09, 36.31)	a	a	35.84 (± 1.25)<br>35.56 (34.99, 36.14)	a,b	a	35.49 (± 0.89)<br>35.39 (34.85, 35.78)	a	a	35.70 (± 1.03)<br>35.72 (35.09, 36.27)	a	a
	8	35.22 (± 1.04)<br>35.05 (34.53, 35.77)	a	a	36.06 (± 1.99)<br>35.49 (34.95, 36.36)	a,b	a	35.63 (± 1.44)<br>35.50 (34.77, 36.90)	a	a	35.60 (± 1.14)<br>35.23 (34.77, 36.09)	a	a
	12	36.36 (± 1.27)<br>36.41 (35.41, 37.24)	a	a	36.81 (± 2.94)<br>35.92 (35.08, 37.13)	b	a	36.21 (± 1.23)<br>36.15 (35.23, 37.52)	a	a	36.20 (± 1.40)<br>36.31 (35.00, 37.37)	a	a
	16	35.42 (± 0.92)<br>35.40 (35.01, 36.06)	a	a	36.49 (± 2.02)<br>35.98 (35.67, 37.20)	b	a	36.70 (± 2.40)<br>36.59 (35.34, 37.47)	a	a	35.98 (± 1.20)<br>35.86 (34.94, 36.80)	a	a
	$\%^{0\to12}$	2.64 (± 6.04)<br>2.30 (0.24, 5.25)		a	5.81 (± 9.03)<br>5.20 (-1.23, 9.40)		a	2.08 (± 5.15)<br>2.45 (-0.68, 5.85)		a	2.19 (± 6.14)<br>2.93 (-1.72, 6.72)		a
	$\%^{0\to16}$	-0.06 (± 4.65)<br>0.63 (-2.72, 2.77)		a	4.80 (± 5.26)<br>3.61 (0.79, 6.78)		a	3.39 (± 6.49)<br>2.56 (-1.34, 4.71)		a	1.49 (± 4.55)<br>1.72 (-0.74, 4.25)		a
C14:0	0	0.35 (± 0.06)<br>0.34 (0.30, 0.40)	a	a	0.31 (± 0.13)<br>0.32 (0.25, 0.37)	a	a	0.31 (± 0.14)<br>0.33 (0.27, 0.44)	a	a	0.35 (± 0.08)<br>0.36 (0.29, 0.39)	a	a
	4	0.29 (± 0.05)<br>0.28 (0.26, 0.32)	a,b	a	0.28 (± 0.07)<br>0.28 (0.22, 0.34)	a	a	0.31 (± 0.06)<br>0.30 (0.28, 0.34)	a	a,b	0.36 (± 0.08)<br>0.36 (0.29, 0.42)	a	b
	8	0.28 (± 0.06)<br>0.28 (0.22, 0.32)	b	a	0.28 (± 0.07)<br>0.27 (0.22, 0.32)	a	a	0.29 (± 0.08)<br>0.29 (0.22, 0.35)	a	a	0.36 (± 0.06)<br>0.34 (0.33, 0.37)	a	b
	12	0.30 (± 0.07)<br>0.29 (0.25, 0.36)	a,b	a	0.29 (± 0.07)<br>0.28 (0.24, 0.33)	a	a	0.30 (± 0.07)<br>0.31 (0.23, 0.36)	a	a	0.36 (± 0.10)<br>0.34 (0.28, 0.40)	a	a
	16	0.30 (± 0.06)<br>0.30 (0.25, 0.33)	b	a	0.28 (± 0.10)<br>0.30 (0.22, 0.33)	a	a	0.34 (± 0.09)<br>0.32 (0.26, 0.41)	a	a	0.34 (± 0.07)<br>0.34 (0.28, 0.36)	a	a
	$\%^{0\to12}$	-10.60 (± 17.40)<br>-7.74 (-21.48, 1.05)		a	-10.11 (± 22.52)<br>-9.85 (-26.55, 1.18)		a	-13.43 (± 23.20)<br>-16.78 (-30.21, -5.04)		a	2.58 (± 20.60)<br>3.28 (-15.35, 21.38)		a
	$\%^{0\to16}$	-13.02 (± 15.98)<br>-16.75 (-25.34, -1.10)		a	-8.92 (± 19.61)<br>-9.26 (-23.34, 10.24)		a	-2.94 (± 22.73)<br>-2.61 (-17.22, 6.57)		a	-1.33 (± 25.13)<br>-8.04 (-22.95, 24.40)		a
C15:0	0	0.17 (± 0.04)<br>0.18 (0.14, 0.20)	a	a	0.17 (± 0.04)<br>0.17 (0.14, 0.18)	a	a	0.18 (± 0.04)<br>0.17 (0.16, 0.21)	a	a	0.18 (± 0.04)<br>0.18 (0.15, 0.20)	a	a
	4	0.18 (± 0.03)<br>0.18 (0.15, 0.19)	a	a	0.17 (± 0.04)<br>0.15 (0.13, 0.20)	a	a	0.18 (± 0.02)<br>0.18 (0.16, 0.19)	a	a	0.18 (± 0.03)<br>0.17 (0.16, 0.19)	a	a
	8	0.17 (± 0.03)<br>0.18 (0.15, 0.20)	a	a	0.17 (± 0.04)<br>0.17 (0.14, 0.19)	a	a	0.18 (± 0.03)<br>0.18 (0.15, 0.21)	a	a	0.20 (± 0.03)<br>0.20 (0.19, 0.21)	b	a
	12	0.18 (± 0.03)<br>0.19 (0.15, 0.20)	a	a	0.17 (± 0.04)<br>0.16 (0.14, 0.21)	a	a	0.18 (± 0.02)<br>0.18 (0.16, 0.20)	a	a	0.18 (± 0.04)<br>0.17 (0.15, 0.19)	a	a
	16	0.17 (± 0.03)<br>0.17 (0.14, 0.20)	a	a	0.16 (± 0.06)<br>0.17 (0.14, 0.19)	a	a	0.18 (± 0.04)<br>0.17 (0.16, 0.20)	a	a	0.17 (± 0.03)<br>0.17 (0.15, 0.19)	a	a

	%$^{0\rightarrow12}$	2.78 (± 15.04)<br>0.97 (-9.47, 12.49)		a	3.45 (± 17.05)<br>-0.42 (-5.83, 13.89)		a	0.98 (± 11.79)<br>-0.52 (-4.30, 10.24)		a	1.11 (± 7.99)<br>1.21 (-4.74, 6.99)		a
	%$^{0\rightarrow16}$	-2.88 (± 8.91)<br>-4.03 (-9.32, 2.12)		a	-2.65 (± 28.10)<br>-0.69 (-11.76, 9.66)		a	2.10 (± 14.95)<br>-1.92 (-8.95, 16.87)		a	-0.62 (± 10.32)<br>-0.51 (-7.73, 4.96)		a
C16:0	0	19.94 (± 1.01)<br>20.23 (19.00, 20.81)	a	a	19.82 (± 0.94)<br>19.98 (19.20, 20.55)	a	a	20.56 (± 1.35)<br>20.41 (19.38, 21.80)	a,b	a	20.24 (± 1.17)<br>20.09 (19.03, 21.23)	a,b	a
	4	20.26 (± 2.69)<br>19.52 (18.72, 20.59)	a	a	20.04 (± 1.56)<br>19.79 (18.78, 20.97)	a	a	19.87 (± 1.11)<br>19.85 (19.22, 20.43)	a	a	20.02 (± 1.18)<br>19.88 (19.22, 20.84)	a	a
	8	20.38 (± 1.21)<br>19.98 (19.79, 21.40)	a	a	21.30 (± 1.93)<br>20.99 (19.85, 22.00)	b	a	20.50 (± 1.40)<br>20.14 (19.67, 20.60)	a,b	a	21.27 (± 1.20)<br>21.19 (20.40, 22.09)	a,b	a
	12	21.18 (± 0.77)<br>21.28 (20.66, 21.75)	b	a	22.00 (± 1.92)<br>21.41 (20.48, 22.89)	c	a	21.21 (± 1.41)<br>21.37 (19.81, 22.44)	b	a	21.19 (± 1.11)<br>21.12 (20.48, 21.80)	b	a
	16	19.95 (± 0.78)<br>19.96 (19.39, 20.66)	a,b	a	21.01 (± 1.70)<br>20.53 (19.68, 21.64)	c	a,b	21.27 (± 1.42)<br>20.70 (20.21, 22.39)	b	b	20.68 (± 1.01)<br>20.48 (20.17, 20.86)	b	a,b
	%$^{0\rightarrow12}$	6.43 (± 6.15)<br>5.33 (1.90, 11.26)		a	11.31 (± 11.46)<br>6.80 (3.72, 18.78)		a	3.44 (± 7.96)<br>6.70 (-2.30, 9.18)		a	5.02 (± 7.56)<br>4.86 (-1.14, 11.78)		a
	%$^{0\rightarrow16}$	0.22 (± 5.42)<br>-0.12 (-4.49, 3.03)		a	6.08 (± 7.83)<br>5.28 (-0.10, 11.14)		b	3.73 (± 8.57)<br>1.61 (-2.53, 7.95)		b	2.45 (± 6.25)<br>3.52 (-0.37, 5.44)		b
C17:0	0	0.29 (± 0.04)<br>0.29 (0.26, 0.32)	a	a	0.28 (± 0.03)<br>0.28 (0.26, 0.30)	a	a	0.29 (± 0.03)<br>0.28 (0.26, 0.32)	a	a	0.29 (± 0.05)<br>0.29 (0.26, 0.31)	a,b	a
	4	0.30 (± 0.05)<br>0.30 (0.27, 0.32)	a,c	a	0.29 (± 0.03)<br>0.28 (0.27, 0.32)	a	a	0.30 (± 0.02)<br>0.30 (0.28, 0.32)	b	a	0.28 (± 0.04)<br>0.29 (0.24, 0.31)	a	a
	8	0.31 (± 0.05)<br>0.32 (0.27, 0.34)	b,c	a	0.30 (± 0.04)<br>0.30 (0.29, 0.33)	b	a	0.31 (± 0.03)<br>0.31 (0.27, 0.33)	b	a	0.31 (± 0.04)<br>0.30 (0.28, 0.35)	b	a
	12	0.31 (± 0.04)<br>0.32 (0.28, 0.35)	b	a	0.31 (± 0.05)<br>0.32 (0.28, 0.34)	b,c	a	0.31 (± 0.03)<br>0.33 (0.28, 0.33)	a,b	a	0.30 (± 0.04)<br>0.31 (0.27, 0.34)	c	a
	16	0.31 (± 0.04)<br>0.31 (0.27, 0.33)	b	a	0.30 (± 0.04)<br>0.30 (0.28, 0.33)	c	a	0.32 (± 0.05)<br>0.30 (0.28, 0.36)	a,b	a	0.30 (± 0.04)<br>0.30 (0.28, 0.33)	b,c	a
	%$^{0\rightarrow12}$	8.11 (± 10.10)<br>6.54 (1.79, 16.60)		a	12.47 (± 14.28)<br>13.38 (4.08, 15.92)		a	8.31 (± 7.63)<br>6.91 (3.99, 14.89)		a	5.11 (± 6.85)<br>6.54 (2.39, 8.42)		a
	%$^{0\rightarrow16}$	5.69 (± 9.31)<br>4.35 (0.33, 9.96)		a	9.00 (± 7.40)<br>9.06 (3.29, 12.46)		a	10.02 (± 12.20)<br>8.25 (0.90, 17.69)		a	6.22 (± 9.36)<br>5.09 (0.68, 11.08)		a
C18:0	0	12.77 (± 1.25)<br>12.31 (11.91, 13.98)	a,b	a	12.44 (± 1.06)<br>12.41 (11.58, 13.12)	a	a	12.39 (± 1.18)<br>12.13 (11.47, 13.11)	a	a	12.49 (± 1.20)<br>12.62 (11.43,13.24)	a	a
	4	13.78 (± 1.37)<br>13.76 (12.67, 14.60)	a	a	12.88 (± 0.90)<br>12.56 (12.27, 13.90)	a	b	12.82 (± 0.98)<br>12.67 (12.21, 13.73)	a	b	12.79 (± 0.70)<br>12.69 (12.46,13.30)	a	b
	8	12.29 (± 1.16)<br>12.15 (11.31, 13.17)	b	a	12.12 (± 0.86)<br>12.33 (11.16, 12.69)	a	a	12.37 (± 1.02)<br>12.03 (11.65, 12.80)	a	a	11.81 (± 0.76)<br>11.70 (11.26,12.21)	a	a
	12	12.57 (± 1.35)<br>12.64 (11.38, 13.44)	a	a	12.35 (± 2.10)<br>12.31 (10.91, 13.40)	a	a	12.25 (± 1.10)<br>12.36 (11.65, 13.12)	a	a	12.38 (± 1.65)<br>12.25 (11.34,13.17)	a	a
	16	12.67 (± 0.79)<br>12.74 (12.15, 13.28)	b	a	12.65 (± 1.45)<br>12.49 (11.79, 13.41)	a	a	12.67 (± 1.66)<br>12.62 (11.50, 13.26)	a	a	12.54 (± 1.08)<br>12.60 (11.74,13.11)	a	a
	%$^{0\rightarrow12}$	-0.45 (± 15.84)		a	0.02 (± 18.61)		a	-0.47 (± 12.15)		a	0.10 (± 16.99)		a

		-4.03 (-12.41, 15.54)			-0.39 (-15.54, 13.39)			0.08 (-11.20, 9.61)			-1.07 (-14.30,13.15)		
	$\%^{0\rightarrow16}$	0.01 (± 10.46)<br>0.58 (-10.78, 7.99)		a	2.47 (± 15.12)<br>1.73 (-10.14, 17.27)		a	2.76 (± 13.87)<br>1.13 (-10.80, 15.02)		a	1.14 (± 11.51)<br>3.37 (-6.21,8.22)		a
**MUFA**													
∑ MUFA	0	15.51 (± 0.77)<br>15.45 (14.77, 16.14)	a	a	15.77 (± 0.90)<br>15.63 (15.10, 16.51)	a,b	a	16.10 (± 0.90)<br>15.78 (15.55, 16.54)	a	a	15.59 (± 0.89)<br>15.70 (14.92, 16.33)	a	a
	4	14.90 (± 0.98)<br>14.90 (14.25, 15.43)	b	a	15.28 (± 0.73)<br>15.24 (14.61, 15.70)	a	a	15.04 (± 0.84)<br>15.25 (14.29, 15.83)	b	a	15.25 (± 1.08)<br>15.21 (14.58, 15.96)	a	a
	8	15.91 (± 0.82)<br>15.97 (15.23, 16.60)	a,c	a	16.34 (± 1.19)<br>16.26 (15.59, 16.65)	b,c	a	15.93 (± 0.82)<br>16.09 (15.03, 16.56)	a	a	16.13 (± 0.64)<br>16.18 (15.58, 16.44)	b,c	a
	12	16.47 (± 0.73)<br>16.44 (16.08, 16.91)	b	a	16.49 (± 1.39)<br>16.57 (15.64, 17.50)	c	a	16.51 (± 0.99)<br>16.17 (15.85, 17.41)	a	a	16.25 (± 1.14)<br>16.21 (15.42, 16.87)	b	a
	16	15.73 (± 0.94)<br>15.73 (15.16, 16.27)	a,c	a	16.26 (± 1.12)<br>16.09 (15.43, 16.69)	b,c	a	16.28 (± 0.70)<br>16.48 (15.40, 16.87)	a	a	16.16 (± 0.96)<br>16.42 (15.15, 16.95)	c	a
	$\%^{0\rightarrow12}$	6.40 (± 6.06)<br>5.75 (1.52, 9.54)		a	4.60 (± 7.34)<br>4.06 (0.59, 8.48)		a	2.75 (± 6.64)<br>2.71 (1.41, 7.23)		a	4.22 (± 4.20)<br>4.48 (2.09, 7.09)		a
	$\%^{0\rightarrow16}$	1.49 (± 5.50)<br>1.56 (-1.82, 5.04)		a	3.19 (± 5.40)<br>2.55 (-2.10, 6.55)		a	1.36 (± 5.77)<br>1.22 (-1.79, 3.23)		a	3.72 (± 4.71)<br>3.24 (0.94, 6.94)		a
C16:1*n*-7	0	0.05 (± 0.01)<br>0.04 (0.04, 0.06)	a	a	0.04 (± 0.01)<br>0.04 (0.04, 0.05)	a,c	a	0.05 (± 0.01)<br>0.04 (0.04, 0.05)	a,b	a	0.05 (± 0.01)<br>0.05 (0.04, 0.06)	a	a
	4	0.04 (± 0.02) 0<br>.04 (0.03, 0.06)	a,b	a	0.04 (± 0.02)<br>0.04 (0.03, 0.06)	b,c	a	0.05 (± 0.02)<br>0.05 (0.04, 0.06)	a,b	a	0.05 (± 0.02)<br>0.04 (0.04, 0.06)	a,b	a
	8	0.03 (± 0.01)<br>0.03 (0.02, 0.03)	b,c	a	0.03 (± 0.01)<br>0.03 (0.02, 0.04)	b	a	0.03 (± 0.01)<br>0.03 (0.03, 0.04)	a	a,b	0.04 (± 0.01)<br>0.04 (0.03, 0.04)	b	b
	12	0.03 (± 0.01)<br>0.03 (0.02, 0.03)	c	a	0.03 (± 0.01)<br>0.03 (0.02, 0.04)	b	a	0.04 (± 0.01)<br>0.03 (0.03, 0.04)	a,b	a	0.03 (± 0.02)<br>0.03 (0.02, 0.04)	a	a
	16	0.07 (± 0.08)<br>0.05 (0.03, 0.09)	a	a	0.06 (± 0.02)<br>0.05 (0.04, 0.07)	a	a	0.11 (± 0.16)<br>0.05 (0.04, 0.09)	b	a	0.07 (± 0.06)<br>0.05 (0.04, 0.07)	a	a
	$\%^{0\rightarrow12}$	-38.97 (± 36.07)<br>-40.28 (-58.82, -23.21)		a	-31.61 (± 39.61)<br>-37.50 (-55.10, -12.82)		a	-12.59 (± 33.85)<br>-19.23 (-39.29, 15.38)		a	-29.33 (± 42.38)<br>-32.61 (-54.38, 0.07)		a
	$\%^{0\rightarrow16}$	86.23 (± 302.71)<br>7.83 (-26.40, 71.57)		a	42.69 (± 69.01)<br>35.90 (-5.00, 56.25)		a	183.72 (± 440.27)<br>12.82 (-23.26, 94.74)		a	68.48 (± 196.99)<br>-8.82 (-25.00, 71.21)		a
C18:1*n*-9	0	14.08 (± 0.75)<br>13.94 (13.43, 14.67)	a	a	14.36 (± 0.91)<br>14.25 (13.65, 14.99)	a,c	a	14.62 (± 0.94)<br>14.34 (14.07, 15.12)	a	a	14.12 (± 0.87)<br>14.10 (13.52, 14.93)	a,c	a
	4	13.36 (± 0.95)<br>13.39 (12.78, 13.86)	b	a	13.79 (± 0.75)<br>13.68 (13.17, 14.24)	a	a	13.49 (± 0.88)<br>13.59 (12.67, 14.40)	b	a	13.78 (± 1.08)<br>13.63 (13.06, 14.51)	a	a
	8	14.34 (± 0.79)<br>14.34 (13.73, 15.06)	a	a	14.79 (± 1.16)<br>14.73 (14.10, 15.12)	b,c	a	14.33 (± 0.81)<br>14.31 (13.34, 14.98)	a	a	14.56 (± 0.65)<br>14.55 (13.99, 15.07)	b	a
	12	14.89 (± 0.71)<br>14.87 (14.65, 15.18)	b	a	14.93 (± 1.35)<br>14.97 (14.08, 16.13)	b	a	14.87 (± 1.01)<br>14.54 (14.17, 15.68)	a	a	14.69 (± 1.11)<br>14.68 (13.86, 15.28)	b	a
	16	14.17 (± 0.89)<br>14.15 (13.72, 14.74)	a,b	a	14.74 (± 1.09)<br>14.57 (14.01, 15.01)	b,c	a	14.61 (± 0.75)<br>14.50 (14.01, 15.29)	a	a	14.59 (± 0.95)<br>14.64 (13.63, 15.44)	b,c	a

	%$^{0\to12}$	5.89 (± 6.00)<br>5.49 (0.92, 9.90)		a	4.03 (± 7.44)<br>3.80 (0.12, 8.12)		a	1.92 (± 7.38)<br>2.30 (0.77, 7.01)		a	4.03 (± 4.41)<br>4.58 (0.87, 6.94)		a
	%$^{0\to16}$	0.67 (± 5.38)<br>1.42 (-2.63, 4.66)		a	2.73 (± 5.56)<br>2.90 (-3.11, 6.94)		a	0.17 (± 6.06)<br>0.40 (-2.95, 2.96)		a	3.43 (± 4.89)<br>4.15 (1.14, 6.56)		a
							**PUFA**						
∑ PUFA	0	39.11 (± 1.93)<br>39.32 (38.53, 40.07)	a	a	39.35 (± 1.00)<br>39.40 (38.51, 40.13)	a	a	39.20 (± 2.22)<br>39.59 (38.46, 40.61)	a,b	a	39.46 (± 1.03)<br>39.47 (38.71, 39.92)	a	a
	4	39.05 (± 2.27)<br>39.33 (37.86, 40.76)	a	a	39.74 (± 0.65)<br>39.68 (39.33, 40.27)	a	a	39.78 (± 1.20)<br>39.82 (39.28, 40.64)	a	a	39.56 (± 1.33)<br>39.50 (38.47, 40.70)	a	a
	8	39.78 (± 0.95)<br>40.02 (39.36, 40.29)	a	a	39.10 (± 1.79)<br>39.30 (38.90, 39.98)	a	a	39.41 (± 1.07)<br>39.66 (38.85, 39.99)	a,b	a	39.75 (± 1.53)<br>39.81 (38.66, 40.91)	a	a
	12	38.53 (± 1.51)<br>38.85 (38.06, 39.43)	a	a	38.13 (± 1.83)<br>38.47 (37.65, 39.15)	b	a	38.90 (± 1.23)<br>39.05 (37.64, 39.98)	a,b	a	38.89 (± 1.66)<br>39.13 (37.55, 40.20)	a	a
	16	39.74 (± 1.21)<br>39.51 (38.78, 40.56)	a	a	38.94 (± 1.24)<br>39.14 (38.30, 39.61)	a,b	a,b	38.53 (± 1.37)<br>38.81 (37.49, 39.35)	b	b	39.21 (± 1.20)<br>38.99 (38.24, 40.06)	a	a
	%$^{0\to12}$	-1.23 (± 6.85)<br>-2.15 (-4.59, 1.01)		a	-3.04 (± 5.21)<br>-2.06 (-4.06, 0.90)		a	-0.36 (± 8.03)<br>-1.13 (-6.46, 2.31)		a	-1.37 (± 4.88)<br>-2.15 (-5.17, 2.77)		a
	%$^{0\to16}$	1.86 (± 6.45)<br>1.48 (-1.74, 3.20)		a	-0.98 (± 3.94)<br>-0.12 (-4.24, 2.10)		a	-1.40 (± 6.61)<br>0.31 (-5.42, 2.31)		a	-0.59 (± 3.56)<br>-1.77 (-2.85, 1.43)		a
∑ *n*-6 PUFA	0	30.84 (± 1.90)<br>30.63 (29.46, 32.78)	a,b	a	31.90 (± 1.43)<br>32.20 (30.79, 33.21)	a	a	31.52 (± 2.23)<br>31.86 (30.68, 32.82)	a	a	31.26 (± 1.44)<br>31.33 (30.23, 32.43)	a	a
	4	30.29 (± 2.20)<br>29.97 (28.83, 31.85)	a,b	a	31.44 (± 1.09)<br>31.61 (30.69, 32.11)	a	a	31.12 (± 1.16)<br>30.74 (30.23, 32.55)	a	a	31.11 (± 1.53)<br>31.30 (30.62, 32.04)	a	a
	8	31.29 (± 1.71)<br>31.64 (29.94, 32.45)	a	a	31.36 (± 1.45)<br>31.32 (30.61, 32.49)	a	a	31.32 (± 1.24)<br>31.36 (30.55, 32.47)	a	a	32.27 (± 1.85)<br>32.56 (31.83, 33.54)	a	a
	12	30.63 (± 1.84)<br>30.44 (29.62, 31.86)	b	a	30.73 (± 1.88)<br>30.65 (29.30, 32.68)	a	a	31.05 (± 1.34)<br>30.76 (30.05, 32.23)	a	a	31.49 (± 1.81)<br>31.60 (30.49, 32.93)	a	a
	16	30.80 (± 2.02)<br>30.26 (29.42, 33.06)	a,b	a	30.99 (± 1.37)<br>30.91 (30.12, 32.22)	a	a	30.62 (± 1.56)<br>30.31 (29.37, 31.30)	a	a	31.37 (± 1.57)<br>31.68 (30.84, 32.62)	a	a
	%$^{0\to12}$	-0.51 (± 5.91)<br>-0.13 (-4.45, 2.98)		a	-3.59 (± 5.58)<br>-2.35 (-5.54, -0.43)		a	-0.91 (± 9.67)<br>-4.17 (-4.79, -0.43)		a	0.75 (± 4.47)<br>0.02 (-2.84, 4.04)		a
	%$^{0\to16}$	0.07 (± 7.00)<br>-1.34 (-4.41, 3.96)		a	-2.78 (± 3.90)<br>-3.73 (-6.28, 1.50)		a	-2.39 (± 8.41)<br>-1.74 (-9.89, 0.46)		a	0.41 (± 4.59)<br>-1.31 (-3.15, 5.32)		a
LA	0	10.53 (± 1.13)<br>10.64 (9.72, 11.54)	a	a	10.59 (± 0.67)<br>10.41 (10.12, 11.16)	a	a	11.18 (± 1.21)<br>11.36 (10.13, 12.30)	a	a	10.58 (± 0.92)<br>10.81 (10.10, 11.04)	a	a
	4	9.41 (± 1.23)<br>9.81 (8.51, 10.18)	b	a	9.93 (± 0.88)<br>9.92 (9.44, 10.50)	b	a	10.38 (± 1.30)<br>10.63 (9.34, 11.44)	b	a	10.15 (± 0.77)<br>10.27 (9.81, 10.72)	a	a
	8	10.25 (± 1.52)<br>10.42 (9.25, 11.23)	a	a	10.74 (± 1.03)<br>10.77 (10.10, 11.22)	a	a	10.55 (± 1.21)<br>10.73 (9.74, 11.67)	b	a	10.87 (± 1.02)<br>10.86 (10.42, 11.69)	a	a
	12	10.30 (± 1.27)<br>10.19 (9.86, 11.14)	a	a	10.48 (± 1.23)<br>10.38 (9.48, 11.22)	a	a	10.84 (± 1.33)<br>11.52 (9.34, 12.00)	a,b	a	10.76 (± 1.14)<br>11.13 (9.80, 11.74)	a	a
	16	10.51 (± 1.19)<br>10.27 (9.61, 11.51)	a	a	10.59 (± 0.80)<br>10.58 (10.11, 11.32)	a	a	11.06 (± 1.14)<br>11.36 (10.05, 11.94)	a	a	10.47 (± 1.16)<br>10.91 (9.79, 11.31)	a	a

	$\%_{0\rightarrow12}$	-1.82 (± 10.56)<br>-0.82 (-10.45, 5.44)		a	-0.97 (± 10.30)<br>-3.50 (-6.33, 5.17)		a	-2.90 (± 7.44)<br>-2.62 (-9.33, 2.65)		a	1.90 (± 9.71)<br>2.25 (-5.57, 8.17)		a
	$\%_{0\rightarrow16}$	0.04 (± 8.36)<br>0.31 (-7.04, 5.78)		a	0.32 (± 8.52)<br>-0.15 (-8.64, 7.59)		a	-0.75 (± 7.95)<br>-0.33 (-7.88, 4.71)		a	-1.01 (± 8.02)<br>2.17 (-6.83, 5.27)		a
GLA	0	0.05 (± 0.02)<br>0.05 (0.04, 0.05)	a	a	0.05 (± 0.01)<br>0.05 (0.04, 0.07)	a	a	0.05 (± 0.02)<br>0.04 (0.03, 0.06)	a	a	0.04 (± 0.02)<br>0.04 (0.03, 0.06)	a	a
	4	0.03 (± 0.01)<br>0.03 (0.02, 0.04)	b	a	0.03 (± 0.01)<br>0.03 (0.03, 0.04)	b	a	0.03 (± 0.02)<br>0.03 (0.02, 0.04)	b	a	0.04 (± 0.02)<br>0.04 (0.02, 0.06)	a	a
	8	0.03 (± 0.01)<br>0.03 (0.02, 0.04)	b	a	0.03 (± 0.01)<br>0.03 (0.02, 0.04)	b	a	0.03 (± 0.03)<br>0.03 (0.02, 0.03)	b	a	0.03 (± 0.01)<br>0.04 (0.02, 0.04)	a	a
	12	0.03 (± 0.02)<br>0.03 (0.02, 0.05)	a,b	a	0.03 (± 0.01)<br>0.03 (0.03, 0.04)	b	a	0.03 (± 0.02)<br>0.03 (0.02, 0.05)	b	a	0.04 (± 0.02)<br>0.03 (0.02, 0.05)	a	a
	16	0.04 (± 0.01)<br>0.03 (0.02, 0.05)	c	a	0.03 (± 0.01)<br>0.03 (0.02, 0.04)	b	a	0.04 (± 0.02)<br>0.03 (0.02, 0.05)	b	a	0.04 (± 0.02)<br>0.03 (0.02, 0.06)	a	a
	$\%_{0\rightarrow12}$	-28.93 (± 34.84)<br>-32.26 (-51.63, -9.36)		a	-32.37 (± 28.49)<br>-30.30 (-47.69, -17.14)		a	-25.39 (± 29.46)<br>-29.79 (-53.66, -6.06)		a	-0.35 (± 38.80)<br>-4.55 (-29.17, 21.94)		a
	$\%_{0\rightarrow16}$	-27.79 (± 21.91)<br>-33.33 (-42.39, -12.50)		a,b	-37.13 (± 18.56)<br>-35.71 (-48.84, -26.42)		a	-19.57 (± 20.94)<br>-21.79 (-31.03, 0.00)		b	5.03 (± 75.50)<br>-22.73 (-33.72, 5.85)		b
C20:3*n*-6	0	1.64 (± 0.28)<br>1.67 (1.34, 1.78)	a	a	1.67 (± 0.27)<br>1.64 (1.40, 1.92)	a	a	1.97 (± 0.42)<br>2.09 (1.52, 2.30)	a	a	1.83 (± 0.46)<br>1.79 (1.53, 2.09)	a	a
	4	1.48 (± 0.30)<br>1.44 (1.26, 1.74)	b	a,b	1.41 (± 0.27)<br>1.39 (1.20, 1.58)	b	b	1.76 (± 0.39)<br>1.80 (1.48, 2.01)	a,b	a	1.78 (± 0.49)<br>1.67 (1.45, 2.04)	a	a
	8	1.46 (± 0.31)<br>1.40 (1.18, 1.75)	b	a,c	1.37 (± 0.31)<br>1.32 (1.15, 1.55)	b	a	1.73 (± 0.38)<br>1.72 (1.46, 2.00)	b	b,c	1.78 (± 0.51)<br>1.66 (1.34, 2.08)	a	b
	12	1.49 (± 0.30)<br>1.54 (1.25, 1.76)	b	a,b	1.35 (± 0.29)<br>1.28 (1.15, 1.50)	b	a	1.69 (± 0.41)<br>1.76 (1.33, 1.95)	b	b	1.76 (± 0.47)<br>1.64 (1.46, 2.03)	a	b
	16	1.50 (± 0.29)<br>1.54 (1.27, 1.69)	b	a,c	1.44 (± 0.27)<br>1.38 (1.23, 1.52)	b	a	1.73 (± 0.36)<br>1.85 (1.38, 2.02)	b	b,c	1.76 (± 0.44)<br>1.70 (1.51, 1.94)	a	b
	$\%_{0\rightarrow12}$	-8.69 (± 10.12)<br>-9.03 (-17.29, -2.06)		a,c	-19.25 (± 9 .71)<br>-21.26 (-28.20, -10.18)		b	-14.36 (± 13.28)<br>-15.86 (-24.63, -5.71)		b,c	-4.18 (± 9.12)<br>-6.31 (-9.59, 0.69)		a
	$\%_{0\rightarrow16}$	-8.46 (± 8.85)<br>-7.37 (-16.45, -2.72)		a,c	-13.71 (± 9.73)<br>-13.99 (-22.42, -4.91)		b	-11.90 (± 11.15)<br>-12.51 (-19.92, -5.39)		b,c	-3.22 (± 11.77)<br>-4.42 (-11.21, 3.12)		c
ARA	0	14.80 (± 0.82)<br>14.89 (14.01, 15.45)	a	a	15.40 (± 0.80)<br>15.39 (14.69, 15.89)	a,b	a	14.40 (± 1.98)<br>14.61 (13.73, 15.45)	a,b	a	14.72 (± 1.03)<br>14.84 (14.00, 15.56)	a	a
	4	15.33 (± 1.29)<br>15.61 (14.93, 16.17)	a	a	15.81 (± 0.92)<br>15.73 (15.08, 16.89)	a	a	14.92 (± 1.01)<br>14.88 (14.17, 15.75)	a,b	a	14.93 (± 1.00)<br>15.17 (14.23, 15.72)	a	a
	8	15.43 (± 1.22)<br>15.09 (14.39, 16.38)	a	a	15.18 (± 1.11)<br>15.26 (14.50, 16.31)	a,b	a	15.06 (± 1.13)<br>14.69 (14.41, 15.93)	a	a	15.16 (± 1.14)<br>15.50 (14.79, 15.73)	a	a
	12	14.92 (± 1.02)<br>14.79 (14.23, 15.81)	a	a	14.95 (± 1.04)<br>14.89 (14.28, 15.78)	b	a	14.78 (± 1.25)<br>14.87 (13.22, 15.89)	b	a	14.78 (± 0.92)<br>14.70 (14.37, 15.53)	a	a
	16	14.93 (± 1.08)<br>14.86 (14.24, 15.95)	a	a	15.02 (± 1.01)<br>14.93 (14.16, 15.93)	b	a	14.24 (± 1.22)<br>14.30 (13.23, 15.40)	b	a	14.95 (± 1.24)<br>14.87 (14.09, 16.09)	a	a
	$\%_{0\rightarrow12}$	1.02 (± 8.06)		a	-2.76 (± 6.63)		a	4.91 (± 21.21)		a	0.63 (± 6.67)		a

		1.88 (-5.13, 6.68)			-1.69 (-5.23, 0.82)			-1.86 (-9.11, 7.94)			-0.67 (-3.68, 6.28)		
	%$^{0\rightarrow16}$	1.06 (± 7.77)<br>2.05 (-4.62, 5.64)		a	-2.39 (± 5.05)<br>-1.48 (-6.37, 1.01)		a	0.84 (± 18.12)<br>-3.86 (-10.03, 6.68)		a	1.72 (± 7.94)<br>0.64 (-3.74, 6.62)		a
C22:4*n*-6	0	3.30 (± 0.67)<br>3.37 (2.99, 3.71)	a,b	a	3.64 (± 0.74)<br>3.53 (3.11, 4.35)	a	a	3.36 (± 0.41)<br>3.26 (3.06, 3.60)	a	a	3.48 (± 0.51)<br>3.36 (3.01, 3.92)	a	a
	4	3.53 (± 0.69)<br>3.47 (3.09, 3.96)	a	a	3.69 (± 0.72)<br>3.60 (3.22, 4.42)	a	a	3.39 (± 0.35)<br>3.30 (3.12, 3.72)	a	a	3.58 (± 0.56)<br>3.55 (3.11, 4.07)	a	a
	8	3.59 (± 0.66)<br>3.61 (3.03, 4.08)	b	a	3.53 (± 0.66)<br>3.48 (3.08, 3.90)	a,b	a	3.39 (± 0.41)<br>3.44 (3.06, 3.65)	a,b	a	3.80 (± 0.72)<br>3.82 (3.43, 4.33)	a	a
	12	3.40 (± 0.68)<br>3.37 (2.83, 3.86)	a,c	a	3.43 (± 0.63)<br>3.38 (3.07, 4.14)	b	a	3.21 (± 0.40)<br>2.98 (2.86, 3.43)	b	a	3.59 (± 0.59)<br>3.50 (3.13, 3.88)	a	a
	16	3.34 (± 0.71)<br>3.14 (2.79, 3.88)	c	a	3.42 (± 0.66)<br>3.24 (3.13, 4.16)	b	a	3.09 (± 0.52)<br>2.94 (2.64, 3.35)	b	a	3.54 (± 0.51)<br>3.56 (3.13, 3.70)	a	a
	%$^{0\rightarrow12}$	5.07 (± 23.39)<br>-0.86 (-5.65, 6.81)		a,b	-5.29 (± 7.78)<br>-4.05 (-8.76, -0.75)		a	-4.32 (± 9.13)<br>-7.22 (-8.84, -0.22)		a	2.93 (± 4.98)<br>2.17 (-1.15, 5.85)		b
	%$^{0\rightarrow16}$	3.05 (± 24.10)<br>-0.07 (-9.93, 4.21)		a,c	-5.84 (± 5.54)<br>-6.81 (-9.92, -0.07)		b,c	-8.10 (± 11.12)<br>-9.52 (-14.27, -3.98)		b	1.94 (± 5.56)<br>2.59 (-2.34, 6.02)		a
C22:5*n*-6	0	0.52 (± 0.14)<br>0.51 (0.43, 0.57)	a,b	a	0.54 (± 0.12)<br>0.55 (0.45, 0.65)	a,b	a,b	0.55 (± 0.19)<br>0.53 (0.44, 0.72)	a	b	0.60 (± 0.11)<br>0.62 (0.51, 0.69)	a	c
	4	0.50 (± 0.12)<br>0.49 (0.44, 0.59)	b	a	0.57 (± 0.11)<br>0.55 (0.49, 0.68)	a	a,b	0.60 (± 0.13)<br>0.60 (0.55, 0.67)	a	a,b	0.63 (± 0.12)<br>0.62 (0.54, 0.71)	a	b
	8	0.52 (± 0.14)<br>0.50 (0.43, 0.59)	b	a	0.49 (± 0.13)<br>0.48 (0.43, 0.58)	c	a	0.55 (± 0.11)<br>0.53 (0.49, 0.59)	a	a	0.61 (± 0.11)<br>0.59 (0.52, 0.69)	a	a
	12	0.45 (± 0.15)<br>0.49 (0.35, 0.54)	a	a	0.48 (± 0.10)<br>0.43 (0.40, 0.58)	c,d	a	0.51 (± 0.12)<br>0.50 (0.43, 0.56)	a	a	0.57 (± 0.14)<br>0.53 (0.48, 0.66)	a	a
	16	0.49 (± 0.16)<br>0.46 (0.42, 0.57)	a	a	0.48 (± 0.12)<br>0.48 (0.38, 0.59)	d	a	0.48 (± 0.16)<br>0.48 (0.37, 0.57)	b	a	0.58 (± 0.12)<br>0.60 (0.50, 0.65)	a	a
	%$^{0\rightarrow12}$	-12.93 (± 20.85)<br>-9.69 (-23.11, 4.51)		a	-8.78 (± 19.53)<br>-14.38 (-20.66, -1.54)		a	-12.96 (± 12.94)<br>-15.58 (-22.50, -5.85)		a	-4.72 (± 19.47)<br>-3.57 (-7.92, 5.96)		a
	%$^{0\rightarrow16}$	-5.29 (± 15.36)<br>-2.79 (-15.22, 5.70)		a	-10.15 (± 20.66)<br>-14.64 (-18.57, -0.90)		a	-18.84 (± 21.15)<br>-10.63 (-30.75, -6.16)		a	-3.82 (± 13.25)<br>-1.00 (-7.17, 3.48)		a
%*n*-6[3]	0	71.57 (± 4.58)<br>71.44 (66.74, 75.25)	a	a	74.49 (± 3.48)<br>74.13 (71.25, 77.38)	a	a	72.94 (± 3.76)<br>73.77 (70.82, 75.88)	a	a	72.02 (± 3.29)<br>72.58 (69.55, 74.73)	a	a
	4	71.22 (± 4.75)<br>71.57 (66.85, 74.17)	a	a	72.90 (± 3.77)<br>73.11 (68.47, 76.16)	a	a	71.41 (± 2.83)<br>71.50 (69.23, 73.76)	b	a	71.85 (± 4.06)<br>72.11 (68.81, 75.88)	a	a
	8	72.16 (± 5.17)<br>72.78 (68.28, 75.67)	a,b	a	73.68 (± 4.27)<br>72.58 (69.55, 77.19)	a	a	72.90 (± 2.56)<br>72.53 (71.09, 74.83)	a,b	a	74.61 (± 3.89)<br>74.80 (72.08, 77.98)	b	a
	12	72.85 (± 4.68)<br>72.34 (68.82, 74.67)	b	a	74.16 (± 4.27)<br>73.30 (69.77, 77.82)	a	a	73.13 (± 4.24)<br>71.11 (69.84, 77.34)	a,b	a	74.34 (± 3.65)<br>74.63 (71.69, 76.67)	b	a
	16	70.13 (± 5.36)<br>69.93 (65.93, 74.55)	a	a	72.77 (± 4.15)<br>71.41 (70.49, 74.27)	a	a	72.09 (± 4.25)<br>71.81 (69.79, 75.59)	a,b	a	73.16 (± 3.92)<br>73.45 (71.07, 75.79)	a,b	a
	%$^{0\rightarrow12}$	1.93 (± 5.34)		a,b	-0.45 (± 3.35)		a	0.37 (± 5.45)		a,b	3.25 (± 2.76)		b

		2.92 (-0.82, 5.07)			-1.50 (-2.37, 1.90)			-0.32 (-4.21, 4.32)			2.15 (1.51, 6.11)		
	$\%^{0\rightarrow16}$	-1.98 (± 4.56)<br>0.15 (-4.64, 0.99)		a,b	-2.28 (± 3.77)<br>-1.04 (-4.93, 0.48)		a	-1.02 (± 6.27)<br>-1.22 (-5.07, 2.17)		a,b	1.60 (± 3.16)<br>1.36 (-0.55, 3.91)		b
∑ *n*-3 PUFA	0	8.26 (± 1.56)<br>8.48 (7.07, 9.73)	a,b	a	7.43 (± 1.03)<br>7.38 (6.87, 8.24)	a	a	7.67 (± 1.10)<br>7.37 (6.81, 8.80)	a	a	8.18 (± 0.94)<br>8.12 (7.31, 9.00)	a	a
	4	8.71 (± 1.84)<br>8.53 (6.75, 10.29)	a	a	8.26 (± 1.26)<br>8.10 (7.52, 9.39)	b	a	8.63 (± 1.20)<br>8.84 (7.46, 9.20)	b	a	8.39 (± 1.29)<br>8.41 (7.04, 9.30)	b	a
	8	8.43 (± 1.83)<br>8.46 (7.20, 9.83)	a,b	a	7.69 (± 1.48)<br>8.07 (6.90, 8.68)	a,b	a	8.03 (± 0.99)<br>8.21 (7.16, 8.74)	a	a	7.44 (± 1.04)<br>7.58 (6.33, 8.48)	a,c	a
	12	7.84 (± 1.59)<br>7.96 (7.02, 8.54)	b	a	7.36 (± 1.46)<br>7.88 (5.99, 8.70)	a	a	7.77 (± 1.47)<br>8.31 (6.60, 8.70)	a,b	a	7.34 (± 1.19)<br>7.52 (6.52, 7.89)	c	a
	16	8.93 (± 1.74)<br>9.01 (7.91, 10.24)	a	a	7.94 (± 1.46)<br>8.08 (7.28, 8.87)	a,b	a	7.90 (± 1.35)<br>7.76 (6.90, 9.16)	a	a	7.82 (± 1.12)<br>7.78 (6.85, 8.57)	a,c	a
	$\%^{0\rightarrow12}$	-3.51 (± 19.82)<br>-7.51 (-17.14, 7.57)		a	-1.13 (± 13.69)<br>-4.03 (-12.14, 10.33)		a	1.60 (± 15.06)<br>3.23 (-11.38, 14.50)		a	-10.33 (± 10.00)<br>-6.74 (-19.67, -2.11)		a
	$\%^{0\rightarrow16}$	8.99 (± 15.97)<br>5.16 (-3.33, 10.53)		a	7.01 (± 15.25)<br>1.90 (-2.15, 16.10)		a	3.40 (± 13.66)<br>2.29 (-8.97, 15.22)		a,b	-4.38 (± 8.66)<br>-5.95 (-10.50, 2.64)		b
ALA	0	0.16 (± 0.04)<br>0.15 (0.12, 0.18)	a	a	0.14 (± 0.06)<br>0.13 (0.12, 0.15)	a	a	0.15 (± 0.04)<br>0.14 (0.13, 0.18)	a	a	0.16 (± 0.05)<br>0.15 (0.12, 0.19)	a	a
	4	0.22 (± 0.12)<br>0.17 (0.13, 0.28)	a,b	a,c	0.26 (± 0.11)<br>0.24 (0.19, 0.36)	b	b,c	0.30 (± 0.13)<br>0.28 (0.23, 0.45)	b	b	0.17 (± 0.09)<br>0.16 (0.12, 0.18)	a	a
	8	0.26 (± 0.15)<br>0.18 (0.14, 0.39)	b,c	a	0.28 (± 0.14)<br>0.27 (0.18, 0.33)	b	a	0.29 (± 0.14)<br>0.26 (0.18, 0.40)	b	a	0.17 (± 0.06)<br>0.17 (0.13, 0.21)	a	a
	12	0.24 (± 0.13)<br>0.20 (0.13, 0.37)	b,c	a,b	0.26 (± 0.17)<br>0.18 (0.15, 0.31)	b	a,b	0.31 (± 0.15)<br>0.29 (0.21, 0.44)	b	a	0.18 (± 0.07)<br>0.16 (0.15, 0.19)	a	b
	16	0.28 (± 0.18)<br>0.22 (0.17, 0.29)	c	a	0.27 (± 0.13)<br>0.25 (0.17, 0.35)	b	a	0.31 (± 0.17)<br>0.27 (0.21, 0.43)	b	a	0.17 (± 0.05)<br>0.17 (0.13, 0.22)	a	b
	$\%^{0\rightarrow12}$	54.23 (± 75.00)<br>41.69 (1.41, 79.80)		a,b	91.92 (± 114.28)<br>50.85 (10.45, 189.29)		a	106.31 (± 89.79)<br>100.93 (29.91, 170.14)		a	10.77 (± 25.19)<br>4.76 (-9.22, 29.58)		b
	$\%^{0\rightarrow16}$	77.04 (± 96.34)<br>52.72 (11.39, 98.32)		a	102.97 (± 95.61)<br>79.14 (36.29, 171.09)		a	106.79 (± 115.89)<br>79.39 (34.87, 123.36)		a	11.71 (± 30.04)<br>11.30 (-19.18, 38.60)		b
C20:4*n*-3	0	0.06 (± 0.02)<br>0.06 (0.05, 0.08)	a	a	0.06 (± 0.02)<br>0.05 (0.04, 0.07)	a	a	0.07 (± 0.02)<br>0.07 (0.05, 0.08)	a	a	0.07 (± 0.03)<br>0.07 (0.05, 0.09)	a	a
	4	0.05 (± 0.02)<br>0.04 (0.04, 0.06)	a	a	0.06 (± 0.03)<br>0.05 (0.03, 0.08)	a	a,c	0.08 (± 0.03)<br>0.09 (0.07, 0.10)	a	b	0.08 (± 0.04)<br>0.07 (0.04, 0.11)	a	b,c
	8	0.05 (± 0.03)<br>0.04 (0.04, 0.06)	a	a	0.06 (± 0.03)<br>0.05 (0.04, 0.09)	a	a	0.07 (± 0.04)<br>0.07 (0.05, 0.09)	a	a	0.07 (± 0.03)<br>0.06 (0.04, 0.08)	a	a
	12	0.06 (± 0.04)<br>0.04 (0.03, 0.07)	a	a	0.05 (± 0.03)<br>0.04 (0.03, 0.09)	a	a	0.08 (± 0.05)<br>0.07 (0.05, 0.11)	a	a	0.06 (± 0.04)<br>0.05 (0.03, 0.07)	a	a
	16	0.07 (± 0.03)<br>0.07 (0.04, 0.07)	a	a	0.06 (± 0.03)<br>0.06 (0.04, 0.08)	a	a	0.09 (± 0.06)<br>0.08 (0.05, 0.11)	a	a	0.07 (± 0.05)<br>0.05 (0.04, 0.10)	a	a
	$\%^{0\rightarrow12}$	-6.94 (± 47.79)		a	-11.41 (± 42.24)		a	24.22 (± 63.26)		a	-17.20 (± 24.89)		a

		-21.78 (-42.20, 36.13)			-22.73 (-35.00, 11.67)			16.42 (-35.71, 40.00)			-17.74 (-36.64, -2.45)		
	%$^{0\to16}$	6.99 (± 47.52)<br>-3.24 (-27.64, 29.91)		a	9.75 (± 39.13)<br>6.52 (-16.67, 26.04)		a	31.53 (± 76.58)<br>11.54 (-8.62, 31.03)		a	-5.40 (± 35.32)<br>-11.11 (-36.50, 22.50)		a
EPA	0	0.85 (± 0.30)<br>0.81 (0.65, 1.07)	a	a	0.70 (± 0.23)<br>0.67 (0.53, 0.87)	a	a	0.66 (± 0.14)<br>0.68 (0.56, 0.75)	a	a	0.68 (± 0.19)<br>0.68 (0.51, 0.84)	a	a
	4	0.82 (± 0.32)<br>0.71 (0.60, 1.14)	a,b	a	0.79 (± 0.26)<br>0.79 (0.58, 1.02)	a	a	0.81 (± 0.21)<br>0.81 (0.67, 0.92)	b	a	0.68 (± 0.21)<br>0.67 (0.51, 0.90)	a,b	a
	8	0.81 (± 0.33)<br>0.71 (0.64, 0.92)	a,b	a	0.76 (± 0.26)<br>0.77 (0.56, 0.96)	a	a	0.72 (± 0.22)<br>0.69 (0.60, 0.94)	a,b	a	0.62 (± 0.18)<br>0.58 (0.45, 0.77)	a,b	a
	12	0.82 (± 0.41)<br>0.75 (0.55, 0.93)	b	a	0.72 (± 0.25)<br>0.78 (0.49, 0.96)	a	a	0.75 (± 0.26)<br>0.68 (0.50, 0.93)	a,b	a	0.59 (± 0.22)<br>0.56 (0.46, 0.68)	b	a
	16	0.93 (± 0.45)<br>0.83 (0.66, 1.23)	a	a	0.82 (± 0.26)<br>0.87 (0.72, 1.02)	a	a	0.73 (± 0.25)<br>0.63 (0.53, 0.97)	a,b	a	0.66 (± 0.21)<br>0.64 (0.50, 0.87)	a,b	a
	%$^{0\to12}$	1.69 (± 52.72)<br>-13.92 (-34.55, 15.23)		a	3.12 (± 22.36)<br>-0.88 (-13.67, 11.62)		a	17.06 (± 43.12)<br>12.43 (-10.39, 31.43)		a	-14.30 (± 13.00)<br>-15.07 (-18.77, -6.71)		a
	%$^{0\to16}$	12.49 (± 46.73)<br>-0.82 (-18.16, 25.37)		a	20.66 (± 37.23)<br>4.62 (-9.79, 62.95)		a	12.61 (± 39.88)<br>4.16 (-16.11, 24.36)		a	-0.47 (± 23.08)<br>-1.74 (-17.23, 12.07)		a
DPA	0	2.55 (± 0.36)<br>2.53 (2.35, 2.76)	a,b	a	2.43 (± 0.30)<br>2.44 (2.15, 2.57)	a,b	a	2.60 (± 0.39)<br>2.49 (2.33, 2.98)	a	a	2.49 (± 0.36)<br>2.39 (2.29, 2.75)	a	a
	4	2.59 (± 0.42)<br>2.50 (2.32, 2.93)	a,b	a	2.55 (± 0.32)<br>2.50 (2.33, 2.87)	a	a	2.73 (± 0.38)<br>2.78 (2.37, 2.94)	a	a	2.54 (± 0.39)<br>2.41 (2.24, 2.95)	a	a
	8	2.63 (± 0.39)<br>2.53 (2.36, 2.83)	a,b	a	2.39 (± 0.43)<br>2.33 (2.21, 2.67)	a,b	a	2.57 (± 0.48)<br>2.73 (2.14, 2.87)	a	a	2.27 (± 0.55)<br>2.27 (2.09, 2.61)	a	a
	12	2.44 (± 0.35)<br>2.44 (2.14, 2.70)	a	a	2.32 (± 0.38)<br>2.37 (1.93, 2.62)	b	a	2.44 (± 0.35)<br>2.47 (2.18, 2.69)	a	a	2.30 (± 0.45)<br>2.28 (1.97, 2.57)	a	a
	16	2.74 (± 0.42)<br>2.71 (2.41, 3.02)	b	a	2.47 (± 0.37)<br>2.54 (2.09, 2.76)	a,b	a	2.52 (± 0.36)<br>2.50 (2.38, 2.76)	a	a	2.40 (± 0.42)<br>2.37 (2.17, 2.66)	a	a
	%$^{0\to12}$	-3.41 (± 14.35)<br>-4.37 (-12.15, 3.92)		a	-4.25 (± 11.92)<br>-5.71 (-14.19, 6.56)		a	-4.80 (± 16.09)<br>-4.09 (-17.05, 11.43)		a	-7.67 (± 10.00)<br>-4.95 (-13.75, 1.70)		a
	%$^{0\to16}$	7.92 (± 12.71)<br>4.89 (-1.70, 17.50)		a	2.23 (± 12.04)<br>2.09 (-4.82, 11.86)		a	-1.68 (± 18.03)<br>1.52 (-12.25, 9.06)		a	-3.90 (± 7.06)<br>-3.88 (-9.64, 1.55)		a
DHA	0	4.59 (± 1.06)<br>4.72 (3.72, 5.55)	a,c	a	4.05 (± 0.77)<br>3.87 (3.50, 4.54)	a	a	4.13 (± 1.09)<br>3.92 (3.13, 5.13)	a	a	4.72 (± 0.84)<br>4.60 (4.11, 5.24)	a,c	a
	4	4.98 (± 1.29)<br>5.08 (3.58, 5.99)	b	a	4.56 (± 0.89)<br>4.45 (3.85, 4.77)	b	a	4.63 (± 1.11)<br>4.59 (3.88, 5.30)	b	a	4.87 (± 1.07)<br>4.67 (4.25, 5.79)	a	a
	8	4.65 (± 1.29)<br>4.89 (3.79, 5.72)	a,b	a	4.16 (± 0.96)<br>3.98 (3.55, 5.07)	b	a	4.34 (± 0.79)<br>4.15 (3.75, 4.97)	b	a	4.26 (± 0.82)<br>4.11 (3.70, 5.05)	b	a
	12	4.25 (± 1.02)<br>4.34 (3.85, 4.77)	a	a	3.96 (± 0.95)<br>3.66 (3.23, 4.95)	a,b	a	4.14 (± 1.08)<br>4.22 (3.13, 5.04)	a,b	a	4.17 (± 0.80)<br>4.12 (3.72, 4.66)	b	a
	16	4.83 (± 0.97)<br>5.07 (4.53, 5.46)	b,c	a	4.24 (± 1.02)<br>3.86 (3.57, 4.77)	a	a	4.17 (± 0.98)<br>4.16 (3.50, 4.71)	a	a	4.45 (± 0.81)<br>4.47 (3.87, 5.04)	b,c	a
	%$^{0\to12}$	-5.62 (± 21.06)<br>-13.17 (-19.90, 11.78)		a	-2.12 (± 14.41)<br>-6.50 (-11.13, 6.93)		a	1.28 (± 16.29)<br>1.82 (-9.82, 12.48)		a	-11.20 (± 11.01)<br>-7.71 (-20.99, -2.81)		a

	$\%^{0\rightarrow16}$	6.85 (± 16.62)<br>2.52 (-2.45, 7.91)		a	4.63 (± 14.60)<br>1.23 (-4.60, 10.02)		a	2.51 (± 14.41)<br>3.37 (-8.25, 12.23)		a	-5.38 (± 11.25) –<br>9.58 (-12.95, 4.28)		a
n-3 LCPUFA	0	7.99 (± 1.53)<br>8.20 (6.84, 9.36)	a,b	a	7.17 (± 0.98)<br>7.16 (6.65, 7.92)	a	a	7.40 (± 1.09)<br>7.04 (6.58, 8.58)	a	a	7.89 (± 0.91)<br>7.71 (7.05, 8.72)	a	a
	4	8.39 (± 1.80)<br>8.28 (6.46, 9.79)	a,b	a	7.89 (± 1.18)<br>7.82 (7.17, 8.83)	b	a	8.18 (± 1.16)<br>8.21 (6.87, 8.82)	b	a	8.09 (± 1.26)<br>8.13 (6.75, 8.74)	a	a
	8	8.08 (± 1.77)<br>8.17 (6.96, 9.51)	a,b	a	7.31 (± 1.43)<br>7.64 (6.68, 8.18)	a,b	a	7.62 (± 0.96)<br>7.71 (6.83, 8.34)	a,b	a	7.15 (± 0.99)<br>7.33 (6.10, 8.08)	b	a
	12	7.50 (± 1.51)<br>7.49 (6.74, 8.25)	a	a	7.00 (± 1.38)<br>7.05 (5.74, 8.40)	b	a	7.33 (± 1.37)<br>7.65 (6.32, 8.26)	a	a	7.06 (± 1.10)<br>7.23 (6.31, 7.61)	b	a
	16	8.50 (± 1.59)<br>8.68 (7.55, 9.51)	b	a	7.53 (± 1.39)<br>7.70 (6.91, 8.11)	a,b	a	7.41 (± 1.19)<br>7.24 (6.53, 8.27)	a	a	7.51 (± 1.08)<br>7.43 (6.62, 8.16)	b	a
	$\%^{0\rightarrow12}$	-4.37 (± 19.92)<br>-8.78 (-17.97, 7.49)		a	-2.53 (± 13.23)<br>-5.05 (-13.36, 6.56)		a	-0.52 (± 14.14)<br>-0.18 (-11.19, 11.14)		a	-10.40 (± 10.17)<br>-6.95 (-20.11, -2.28)		a
	$\%^{0\rightarrow16}$	7.53 (± 15.36)<br>3.55 (-3.18, 8.89)		a	5.16 (± 14.64)<br>1.70 (-4.32, 12.21)		a	0.85 (± 12.76)<br>1.44 (-9.43, 13.39)		a,b	-4.77 (± 8.85)<br>-7.15 (-10.51, 2.79)		b
n-3 Index	0	5.44 (± 1.28)<br>5.67 (4.33, 6.41)	a,b	a	4.75 (± 0.89)<br>4.58 (4.27, 5.38)	a	a	4.79 (± 1.17)<br>4.58 (3.69, 5.95)	a	a	5.40 (± 0.91)<br>5.18 (4.72, 6.16)	a,c	a
	4	5.80 (± 1.56)<br>5.83 (3.94, 7.20)	a,b	a	5.35 (± 1.04)<br>5.19 (4.58, 5.87)	b	a	5.45 (± 1.21)<br>5.58 (4.63, 5.98)	b	a	5.55 (± 1.19)<br>5.45 (4.72, 6.25)	a,c	a
	8	5.45 (± 1.51)<br>5.67 (4.45, 6.68)	a,b	a	4.92 (± 1.13)<br>5.08 (4.40, 5.84)	a,b	a	5.05 (± 0.90)<br>4.84 (4.37, 6.03)	a,b	a	4.88 (± 0.91)<br>5.08 (4.11, 5.65)	b	a
	12	5.06 (± 1.27)<br>5.03 (4.57, 5.84)	a	a	4.68 (± 1.13)<br>4.58 (3.81, 5.86)	b	a	4.89 (± 1.29)<br>4.90 (3.72, 6.09)	a	a	4.76 (± 0.95)<br>4.64 (4.23, 5.28)	b	a
	16	5.77 (± 1.31)<br>5.99 (5.20, 6.39)	b	a	5.06 (± 1.20)<br>4.98 (4.32, 5.58)	a,b	a	4.90 (± 1.18)<br>4.77 (3.99, 5.54)	a	a	5.11 (± 0.93)<br>5.08 (4.46, 5.81)	b,c	a
	$\%^{0\rightarrow12}$	-4.84 (± 22.82)<br>-12.19 (-20.49, 9.94)		a	-1.51 (± 14.33)<br>-2.79 (-11.62, 7.47)		a	2.73 (± 16.24)<br>-1.35 (-5.83, 11.99)		a	-11.64 (± 10.63)<br>-8.68 (-21.74, -3.32)		a
	$\%^{0\rightarrow16}$	7.35 (± 17.39)<br>0.84 (-4.42, 9.35)		a	6.71 (± 16.74)<br>1.53 (-3.90, 15.53)		a	3.25 (± 14.46)<br>1.72 (-9.47, 15.34)		a,b	-5.01 (± 11.35)<br>-8.99 (-12.60, 4.59)		b
%*n*-3[4]	0	28.28 (± 4.59)<br>28.41 (24.59, 33.11)	a	a	25.34 (± 3.48)<br>25.67 (22.48, 28.59)	a	a	26.92 (± 3.75)<br>26.10 (23.98, 29.02)	a	a	27.82 (± 3.29)<br>27.24 (25.09, 30.27)	a	a
	4	28.65 (± 4.75)<br>28.29 (25.70, 32.91)	a	a	26.96 (± 3.75)<br>26.76 (23.74, 31.29)	a	a	28.43 (± 2.83)<br>28.38 (26.03, 30.66)	b	a	28.03 (± 4.05)<br>27.79 (23.99, 31.07)	a	a
	8	27.75 (± 5.18)<br>27.13 (24.24, 31.63)	a,b	a	26.21 (± 4.27)<br>27.30 (22.71, 30.35)	a	a	27.00 (± 2.57)<br>27.37 (25.08, 28.83)	a,b	a	25.30 (± 3.89)<br>25.09 (21.90, 27.84)	b	a
	12	27.06 (± 4.69)<br>27.58 (25.25, 31.11)	b	a	25.74 (± 4.27)<br>26.60 (22.07, 30.15)	a	a	26.78 (± 4.24)<br>28.79 (22.57, 30.07)	a,b	a	25.58 (± 3.65)<br>25.27 (23.24, 28.21)	b	a
	16	29.66 (± 5.32)<br>29.84 (25.29, 33.83)	a	a	27.04 (± 4.14)<br>28.40 (25.65, 29.20)	a	a	27.69 (± 4.19)<br>27.97 (24.32, 30.01)	a,b	a	26.66 (± 3.91)<br>26.44 (24.03, 28.75)	a,b	a
	$\%^{0\rightarrow12}$	-3.65 (± 13.77)<br>-7.99 (-12.59, 2.59)		a,b	1.53 (± 9.78)<br>3.88 (-5.28, 7.47)		a	-0.09 (± 13.11)<br>1.35 (-14.45, 12.42)		a,b	-8.12 (± 6.77)<br>-6.75 (-13.48, -3.57)		b

	$\%^{0\rightarrow16}$	5.19 (± 12.37)<br>-0.35 (-3.77, 12.91)		a,b	6.87 (± 11.00)<br>2.18 (-1.09, 16.08)		a	3.52 (± 13.84)<br>3.66 (-5.85, 13.98)		a,b	-4.18 (± 8.39)<br>-3.60 (-10.51, 1.32)		b
**TFA**													
TFA	0	0.83 (± 0.26)<br>0.83 (0.63, 0.96)	a	a	0.85 (± 0.39)<br>0.81 (0.52, 1.31)	a	a	0.87 (± 0.32)<br>0.80 (0.63, 1.10)	a,b	a	0.89 (± 0.28)<br>0.80 (0.67, 1.06)	a	a
	4	0.75 (± 0.33)<br>0.62 (0.54, 0.93)	a	a	0.60 (± 0.17)<br>0.57 (0.48, 0.68)	b	a	0.69 (± 0.25)<br>0.61 (0.53, 0.71)	a	a	0.84 (± 0.35)<br>0.71 (0.60, 0.94)	a	a
	8	0.97 (± 0.36)<br>0.94 (0.65, 1.37)	a	a	0.88 (± 0.34)<br>0.86 (0.59, 1.19)	a	a	0.83 (± 0.23)<br>0.80 (0.67, 1.00)	a,b	a	1.00 (± 0.25)<br>1.05 (0.88, 1.14)	a	a
	12	0.97 (± 0.35)<br>0.95 (0.69, 1.22)	a	a	0.82 (± 0.27)<br>0.85 (0.55, 1.08)	a	a	1.04 (± 0.40)<br>1.11 (0.70, 1.44)	b	a	1.13 (± 0.42)<br>1.06 (0.72, 1.46)	a	a
	16	0.82 (± 0.27)<br>0.74 (0.62, 1.00)	a	a	0.75 (± 0.28)<br>0.70 (0.53, 0.93)	a	a	1.08 (± 0.36)<br>0.95 (0.77, 1.41)	b	a	0.93 (± 0.26)<br>0.96 (0.63, 1.08)	a	a
	$\%^{0\rightarrow12}$	29.95 (± 65.27)<br>17.39 (-16.29, 65.98)		a	16.00 (± 67.60)<br>-8.27 (-32.32, 44.96)		a	28.43 (± 59.46)<br>11.96 (-17.93, 52.77)		a	42.85 (± 81.39)<br>1.99 (-20.88, 84.91)		a
	$\%^{0\rightarrow16}$	9.16 (± 58.67)<br>-5.31 (-27.26, 19.50)		a	-1.04 (± 36.52)<br>-0.28 (-35.21, 28.34)		a	35.27 (± 51.29)<br>25.36 (7.98, 61.71)		a	12.53 (± 46.28)<br>-4.53 (-21.54, 48.17)		a
**CLA**													
CLA	0	0.17 (± 0.06)<br>0.17 (0.13, 0.21)	a	a	0.19 (± 0.05)<br>0.18 (0.15, 0.22)	a,b	a	0.18 (± 0.05)<br>0.17 (0.15, 0.21)	a	a	0.20 (± 0.06)<br>0.20 (0.15, 0.23)	a,b	a
	4	0.17 (± 0.07)<br>0.19 (0.15, 0.22)	a	a	0.20 (± 0.06)<br>0.20 (0.19, 0.22)	b	a	0.19 (± 0.07)<br>0.19 (0.18, 0.25)	a	a	0.21 (± 0.04)<br>0.21 (0.17, 0.24)	a	a
	8	0.16 (± 0.04)<br>0.17 (0.14, 0.19)	a	a	0.16 (± 0.05)<br>0.16 (0.12, 0.19)	a	a	0.18 (± 0.07)<br>0.20 (0.17, 0.22)	a	a	0.17 (± 0.06)<br>0.16 (0.13, 0.22)	b	a
	12	0.17 (± 0.07)<br>0.17 (0.16, 0.20)	a	a	0.16 (± 0.07)<br>0.18 (0.12, 0.21)	a	a	0.19 (± 0.07)<br>0.17 (0.16, 0.21)	a	a	0.17 (± 0.04)<br>0.18 (0.13, 0.20)	a,b	a
	16	0.19 (± 0.05)<br>0.19 (0.16, 0.22)	a	a	0.17 (± 0.05)<br>0.18 (0.12, 0.21)	a,b	a	0.17 (± 0.08)<br>0.18 (0.15, 0.20)	a	a	0.19 (± 0.06)<br>0.19 (0.13, 0.23)	a,b	a
	$\%^{0\rightarrow12}$	6.06 (± 53.27)<br>2.65 (-20.23, 39.88)		a	-15.31 (± 37.03)<br>-19.11 (-33.70, 10.00)		a	34.67 (± 121.23)<br>-4.24 (-14.36, 38.54)		a	-9.33 (± 32.27)<br>-11.11 (-37.72, 12.23)		a
	$\%^{0\rightarrow16}$	10.73 (± 43.04)<br>0.38 (-28.61, 47.02)		a	-1.51 (± 41.89)<br>-8.47 (-39.06, 22.93)		a	-2.78 (± 43.50)<br>-10.34 (-36.19, 29.14)		a	-1.44 (± 44.45)<br>-13.67 (-37.07, 21.10)		a
**Ratios**													
n-6/*n*-3	0	3.89 (± 0.91)<br>3.61 (3.02, 4.71)	a,b	a	4.39 (± 0.79)<br>4.25 (3.66, 4.98)	a	a	4.20 (± 0.73)<br>4.26 (3.49, 4.83)	a	a	3.88 (± 0.55)<br>3.96 (3.51, 4.37)	a	a
	4	3.65 (± 0.92)<br>3.49 (2.94, 4.38)	a	a	3.91 (± 0.72)<br>3.92 (3.28, 4.25)	b	a	3.68 (± 0.59)<br>3.63 (3.29, 4.19)	b	a	3.80 (± 0.69)<br>3.71 (3.13, 4.45)	a	a
	8	3.93 (± 1.12)<br>3.77 (3.06, 4.57)	a,b	a	4.27 (± 1.09)<br>3.82 (3.53, 4.80)	a,b	a	3.97 (± 0.61)<br>3.85 (3.39, 4.43)	a,b	a	4.44 (± 0.78)<br>4.13 (3.84, 5.04)	b	a
	12	4.11 (± 1.14)<br>3.91 (3.29, 4.30)	b	a	4.38 (± 1.14)<br>4.12 (3.53, 5.34)	a,b	a	4.16 (± 0.94)<br>3.70 (3.51, 4.89)	a	a	4.42 (± 0.86)<br>4.36 (3.79, 4.88)	b	a

	16	3.62 (± 0.98)<br>3.36 (2.91, 4.08)	a	a	4.06 (± 0.97)<br>3.84 (3.49, 4.40)	a,b	a	4.00 (± 0.83)<br>3.91 (3.38, 4.46)	a,b	a	4.10 (± 0.72)<br>4.05 (3.62, 4.74)	a,b	a
	$\%^{0\rightarrow12}$	6.79 (± 20.27)<br>8.90 (-7.46, 21.22)		a,b	-0.54 (± 16.73)<br>-7.23 (-11.39, 10.71)		a	-0.50 (± 16.94)<br>-3.64 (-16.69, 16.22)		a,b	13.57 (±12.44)<br>9.57 (4.41, 23.34)		b
	$\%^{0\rightarrow16}$	-6.63 (± 13.04)<br>-3.87 (-17.84, 3.46)		a	-7.37 (± 13.73)<br>-3.61 (-16.53, 2.27)		a	-3.84 (± 17.02)<br>-5.83 (-19.08, 6.31)		a	5.94 (± 11.93)<br>3.32 (-1.69, 14.15)		a
ARA/EPA	0	19.88 (± 7.89)<br>19.37 (13.62, 22.87)	a	a	24.45 (± 8.56)<br>22.56 (17.61, 29.82)	a	a	22.79 (± 5.71)<br>22.75 (18.95, 26.02)	a	a	23.88 (± 8.14)<br>24.08 (16.69, 28.92)	a	a
	4	22.06 (± 10.84)<br>19.66 (13.42, 26.51)	a,b	a	23.07 (± 10.70)<br>19.05 (16.32, 26.11)	a	a	19.53 (± 5.29)<br>19.00 (16.27, 22.38)	b	a	24.59 (± 9.67)<br>23.18 (16.31, 28.61)	a	a
	8	22.16 (± 8.97)<br>21.77 (17.35, 24.85)	a,b	a	23.43 (± 11.64)<br>19.56 (15.24, 24.92)	a	a	23.36 (± 8.83)<br>20.83 (15.98, 28.75)	a	a	26.80 (± 8.14)<br>27.38 (17.90, 35.99)	a	a
	12	22.63 (± 11.03)<br>20.56 (15.27, 27.72)	b	a	24.51 (± 12.65)<br>18.62 (16.49, 32.74)	a	a	22.18 (± 8.68)<br>21.51 (14.93, 29.17)	a	a	29.34 (± 14.39)<br>27.39 (20.42, 32.45)	a	a
	16	19.70 (± 9.90)<br>17.94 (11.65, 24.16)	a	a	21.58 (± 11.75)<br>17.10 (14.99, 20.42)	a	a	22.05 (± 8.37)<br>21.16 (14.65, 29.28)	a	a	25.43 (± 10.39)<br>23.13 (17.55, 30.20)	a	a
	$\%^{0\rightarrow12}$	19.06 (± 46.27)<br>23.99 (-6.15, 37.18)		a	-1.51 (± 22.44)<br>-8.20 (-25.14, 11.27)		a	2.03 (± 43.90)<br>-7.21 (-30.91, 35.44)		a	20.58 (± 24.77)<br>16.56 (10.75, 23.53)		a
	$\%^{0\rightarrow16}$	0.56 (± 31.31)<br>1.01 (-23.50, 27.63)		a	-11.79 (± 25.97)<br>-9.96 (-41.29, 8.04)		a	2.66 (± 51.35)<br>-12.85 (-29.40, 25.50)		a	8.79 (± 33.14)<br>5.63 (-12.58, 21.15)		a
ARA/DHA	0	3.42 (± 0.94)<br>3.29 (2.62, 4.00)	a,b	a	3.95 (± 0.85)<br>3.83 (3.24, 4.66)	a	a	3.72 (± 1.03)<br>3.63 (2.93, 4.48)	a	a	3.23 (± 0.70)<br>3.11 (2.77, 3.62)	a,c	a
	4	3.30 (± 1.03)<br>3.15 (2.54, 3.67)	a	a	3.60 (± 0.77)<br>3.43 (3.13, 4.24)	b	a	3.37 (± 0.75)<br>3.24 (2.85, 3.71)	a	a	3.21 (± 0.78)<br>3.17 (2.77, 3.56)	a	a
	8	3.68 (± 1.48)<br>3.27 (2.57, 3.97)	a,b	a	3.85 (± 0.98)<br>3.79 (3.01, 4.36)	a,b	a	3.58 (± 0.69)<br>3.58 (3.02, 4.07)	a	a	3.71 (± 0.88)<br>3.84 (3.02, 4.10)	b,c	a
	12	3.75 (± 1.15)<br>3.39 (3.12, 3.93)	b	a	3.98 (± 0.97)<br>4.13 (3.06, 4.45)	a	a	3.83 (± 1.16)<br>3.45 (3.04, 4.26)	a	a	3.67 (± 0.79)<br>3.68 (3.14, 3.98)	b,c	a
	16	3.27 (± 0.99)<br>2.98 (2.60, 3.52)	a	a	3.74 (± 0.95)<br>3.57 (2.96, 4.29)	a,b	a	3.63 (± 1.07)<br>3.35 (2.98, 4.24)	a	a	3.50 (± 0.85)<br>3.46 (2.96, 3.77)	c	a
	$\%^{0\rightarrow12}$	10.64 (± 18.64)<br>14.80 (-1.85, 22.02)		a,b	0.93 (± 13.27)<br>1.03 (-8.10, 5.29)		a	5.52 (± 24.02)<br>3.19 (-5.39, 10.29)		a,b	14.56 (± 12.42)<br>13.35 (3.99, 26.31)		b
	$\%^{0\rightarrow16}$	-4.03 (± 11.83)<br>-0.51 (-13.27, 5.13)		a	-5.36 (± 11.35) –<br>4.12 (-10.07, 3.43)		a	0.87 (± 28.01)<br>-8.30 (-16.97, 11.69)		a	8.91 (± 15.89)<br>4.67 (-2.28, 18.45)		a
D9D	0	1.12 (± 0.15)<br>1.12 (0.98, 1.23)	a	a	1.16 (± 0.13)<br>1.21 (1.07, 1.26)	a	a	1.19 (± 0.14)<br>1.18 (1.09, 1.34)	a,b	a	1.14 (± 0.12)<br>1.13 (1.05, 1.23)	a,b	a
	4	0.98 (± 0.13)<br>0.96 (0.88, 1.08)	b	a	1.07 (± 0.08)<br>1.09 (1.04, 1.14)	b	a	1.06 (± 0.11)<br>1.02 (0.98, 1.18)	a	a	1.08 (± 0.12)<br>1.05 (0.99, 1.11)	a	a
	8	1.18 (± 0.13)<br>1.15 (1.11, 1.30)	b	a	1.22 (± 0.08)<br>1.23 (1.16, 1.27)	b	a	1.17 (± 0.13)<br>1.23 (1.06, 1.24)	a,b	a	1.24 (± 0.09)<br>1.27 (1.18, 1.30)	b	a
	12	1.20 (± 0.13)<br>1.18 (1.08, 1.31)	b	a	1.24 (± 0.21)<br>1.29 (1.16, 1.34)	b	a	1.22 (± 0.14)<br>1.22 (1.10, 1.31)	b	a	1.20 (± 0.17)<br>1.18 (1.06, 1.29)	a,b	a
	16	1.12 (± 0.12)	b	a	1.18 (± 0.14)	b	a	1.17 (± 0.14)	a,b	a	1.17 (± 0.13)	a,b	a

		1.12 (1.04, 1.19)			1.18 (1.08, 1.29)			1.20 (1.05, 1.25)			1.18 (1.09, 1.26)		
	$\%^{0\rightarrow12}$	9.12 (± 19.72)<br>5.32 (-5.13, 24.22)		a	8.25 (± 25.27)<br>3.81 (-9.45, 20.41)		a	4.06 (± 16.45)<br>3.50 (-10.09, 17.16)		a	6.93 (± 19.65)<br>4.88 (-10.13, 23.98)		a
	$\%^{0\rightarrow16}$	2.03 (± 14.81)<br>-1.03 (-8.50, 15.16)		a	2.61 (± 17.73)<br>2.49 (-14.68, 16.02)		a	-0.82 (± 15.21)<br>-4.18 (-11.61, 12.26)		a	3.52 (± 13.09)<br>-0.69 (-5.73, 8.60)		a
n-6 D5D	0	9.30 (± 1.64)<br>9.25 (8.08, 10.81)	a	a	9.48 (± 1.93)<br>9.16 (7.70, 11.34)	a	a	7.66 (± 2.20)<br>7.23 (5.97, 9.25)	a	a	8.51 (± 2.26)<br>8.07 (6.88, 9.81)	a	a
	4	10.69 (± 1.96)<br>11.06 (8.89, 12.28)	b	a,b	11.72 (± 2.76)<br>11.36 (9.53, 13.35)	b	a	8.97 (± 2.53)<br>8.80 (7.06, 9.92)	b,c	b	8.98 (± 2.51)<br>8.55 (7.52, 11.09)	a	b
	8	11.06 (± 2.53)<br>10.89 (8.44, 13.35)	b	a,b	11.61 (± 2.74)<br>12.43 (9.51, 13.79)	b	a	9.26 (± 2.86)<br>8.19 (7.29, 10.92)	b	b	9.15 (± 2.73)<br>8.29 (7.45, 11.42)	a	b
	12	10.34 (± 1.96)<br>9.66 (8.50, 11.95)	b	a,b	11.57 (± 2.88)<br>12.35 (9.53, 12.93)	b	a	9.37 (± 2.92)<br>8.51 (7.42, 10.96)	c	b	8.97 (± 2.52)<br>8.73 (7.26, 10.24)	a	b
	16	10.30 (± 1.95)<br>10.34 (8.35, 11.41)	b	a	10.79 (± 2.18)<br>10.78 (9.36, 12.85)	b	a	8.72 (± 2.54)<br>7.72 (6.66, 10.99)	c	a	8.99 (± 2.46)<br>8.53 (7.70, 10.55)	a	a
	$\%^{0\rightarrow12}$	11.47 (± 10.88)<br>14.15 (5.01, 20.62)		a,b	22.04 (± 16.88)<br>23.72 (6.14, 33.00)		a	23.60 (± 21.08)<br>18.52 (8.64, 36.72)		b	5.60 (± 9.38)<br>5.47 (-3.79, 14.36)		b
	$\%^{0\rightarrow16}$	10.97 (± 9.49)<br>11.63 (5.95, 17.28)		a	14.71 (± 15.97)<br>13.41 (-0.83, 28.86)		a	15.26 (± 19.30)<br>9.95 (3.54, 19.42)		a	6.45 (± 15.15)<br>1.31 (-1.22, 15.01)		a
n-3 D5D	0	13.83 (± 4.47)<br>14.03 (10.58, 16.94)	a	a	13.35 (± 4.01)<br>13.37 (10.65, 14.28)	a	a	10.48 (± 4.08)<br>9.65 (6.83, 12.81)	a	a	10.44 (± 4.17)<br>9.47 (7.11, 13.18)	a	a
	4	17.58 (± 7.15)<br>16.49 (13.52, 22.91)	a	a	16.94 (± 6.93)<br>15.94 (11.81, 19.68)	a	a	10.81 (± 5.14)<br>10.21 (7.19, 12.17)	a	b	11.24 (± 5.93)<br>9.65 (6.10, 16.13)	a	b
	8	16.34 (± 5.37)<br>17.25 (12.74, 21.41)	a	a	14.30 (± 5.07)<br>13.74 (9.51, 19.34)	a	a,b	11.55 (± 6.31)<br>11.31 (6.36, 13.33)	a	b	10.24 (± 3.98)<br>9.93 (6.65, 12.59)	a	b
	12	15.50 (± 4.72)<br>16.00 (11.43, 19.41)	a	a	15.79 (± 5.36)<br>16.19 (9.81, 20.78)	a	a	11.01 (± 5.91)<br>8.80 (7.68, 13.78)	a	b	11.72 (± 6.19)<br>10.07 (6.83, 17.80)	a	b
	16	14.79 (± 4.64)<br>13.39 (10.89, 18.73)	a	a	15.00 (± 4.68)<br>13.93 (10.84, 18.98)	a	a	10.43 (± 6.70)<br>9.07 (5.58, 12.31)	a	b	12.18 (± 6.84)<br>10.52 (6.74, 16.02)	a	a,b
	$\%^{0\rightarrow12}$	19.40 (± 43.33)<br>23.55 (-21.26, 43.44)		a	22.22 (± 42.88)<br>13.99 (-12.06, 50.50)		a	5.14 (± 33.94)<br>1.31 (-22.04, 14.29)		a	10.41 (± 29.62)<br>6.10 (-6.23, 29.79)		a
	$\%^{0\rightarrow16}$	8.82 (± 20.36)<br>9.10 (-7.60, 18.60)		a	16.05 (± 35.77)<br>14.59 (-13.16, 33.57)		a	-6.29 (± 23.83)<br>-10.66 (-21.40, 7.23)		a	14.33 (± 35.14)<br>15.40 (-9.97, 30.47)		a
n-6 D6D	0	0.16 (± 0.04)<br>0.15 (0.13, 0.19)	a	a	0.16 (± 0.03)<br>0.16 (0.14, 0.17)	a	a	0.18 (± 0.04)<br>0.18 (0.14, 0.21)	a	a	0.18 (± 0.05)<br>0.17 (0.14, 0.21)	a	a
	4	0.16 (± 0.04)<br>0.15 (0.13, 0.19)	a	a	0.14 (± 0.02)<br>0.15 (0.13, 0.16)	b	a	0.17 (± 0.03)<br>0.18 (0.15, 0.20)	a	a	0.18 (± 0.05)<br>0.16 (0.14, 0.22)	a	a
	8	0.14 (0.12, 0.18)	b	a,b	0.13 (± 0.03)<br>0.13 (0.11, 0.15)	c	a	0.16 (± 0.03)<br>0.16 (0.14, 0.19)	a	b	0.17 (± 0.05)<br>0.16 (0.13, 0.17)	a	b
	12	0.15 (± 0.04)<br>0.14 (0.12, 0.17)	a,b	a,b	0.13 (± 0.02)<br>0.13 (0.12, 0.14)	b,c	a	0.16 (± 0.04)<br>0.16 (0.12, 0.19)	a	a,b	0.17 (± 0.05)<br>0.15 (0.13, 0.20)	a	b
	16	0.14 (± 0.03)<br>0.14 (0.12, 0.17)	b	a	0.14 (± 0.02)<br>0.14 (0.13, 0.15)	b,c	a	0.16 (± 0.03)<br>0.16 (0.12, 0.18)	a	a	0.17 (± 0.05)<br>0.15 (0.13, 0.21)	a	a

	$\%_{0\rightarrow12}$	-6.04 (± 14.00)<br>-6.96 (-15.63, 3.84)		a	-18.15 (± 8.95)<br>-18.87 (-25.56, -9.95)		b	-11.75 (± 12.25)<br>-16.21 (-22.08, 0.19)		a,b	-5.46 (± 9.91)<br>-6.71 (-12.98, 0.97)		a
	$\%_{0\rightarrow16}$	-8.04 (± 10.27)<br>-6.61 (-16.49, 1.91)		a,b	-13.82 (± 8.22)<br>-11.95 (-18.74, -8.18)		a	-10.96 (± 11.34)<br>-9.82 (-21.48, -2.48)		a,b	-2.04 (± 10.52)<br>-3.05 (-10.21, 4.33)		b
n-3 D6D	0	5.58 (± 2.01)<br>5.31 (4.21, 6.42)	a	a	5.04 (± 1.28)<br>4.97 (4.20, 5.79)	a	a	4.47 (± 1.16)<br>4.15 (3.73, 5.02)	a	a	4.30 (± 0.86)<br>4.24 (3.50, 4.98)	a	a
	4	4.36 (± 2.14)<br>4.10 (2.72, 5.97)	a,b	a	3.29 (± 1.03)<br>3.20 (2.53, 3.87)	b	a	3.03 (± 1.20)<br>2.73 (2.28, 4.04)	b	a	4.26 (± 1.34)<br>4.29 (3.20, 4.67)	a	a
	8	3.80 (± 1.77)<br>3.21 (2.62, 5.27)	b	a	3.09 (± 1.31)<br>2.85 (2.25, 3.38)	b	a	2.80 (± 1.08)<br>2.58 (2.13, 3.88)	b	a	3.62 (± 0.65)<br>3.74 (3.14, 4.03)	a,b	a
	12	3.81 (± 1.74)<br>3.35 (2.62, 4.48)	b	a	3.37 (± 1.53)<br>3.13 (2.11, 4.65)	b	a	2.90 (± 1.54)<br>2.46 (1.74, 4.08)	b	a	3.38 (± 0.78)<br>3.33 (2.83, 3.78)	b	a
	16	3.78 (± 1.39)<br>3.66 (2.84, 4.12)	b	a	3.26 (± 1.12)<br>3.02 (2.38, 4.19)	b	a	2.75 (± 1.28)<br>2.42 (1.67, 3.48)	b	a	3.88 (± 0.88)<br>3.92 (3.49, 4.09)	a,b	b
	$\%_{0\rightarrow12}$	-29.37 (± 28.21)<br>-29.78 (-48.97, -17.51)		a	-28.25 (± 41.97)<br>-36.59 (-63.18, -14.79)		a	-36.70 (± 26.74)<br>-45.38 (-53.19, -34.59)		a	-19.91 (± 17.84)<br>-22.03 (-35.04, -7.49)		a
	$\%_{0\rightarrow16}$	-28.48 (± 26.98)<br>-28.54 (-40.87, -21.94)		a	-33.61 (± 21.38)<br>-32.68 (-48.75, -12.55)		a	-38.18 (± 24.79)<br>-46.29 (-56.06, -22.86)		a	-7.72 (± 20.59)<br>-11.89 (-21.39, 8.99)		b

Variable expressed as mean (±SD) and as median (25th, 75th percentile); [1]comparison of time points within the respective diet group (T; column by column); [2]comparison between diet groups at individual time points (G; line by line); time points or groups without a common letter are significantly different, $p < 0.05$; Abbreviations: t = time point (week), % = percentual change from start of intervention, SFA = saturated fatty acids, MUFA = monounsaturated fatty acids, PUFA = polyunsaturated fatty acids, *n* = omega, LC = long-chain, HUFA = highly unsaturated fatty acids, TFA = trans fatty acids, CLA = conjugated linoleic acids, ARA = arachidonic acid, EPA = eicosapentaenoic acid, DPA = docosapentaenoic acid, DHA = docosahexaenoic acid, LA = linoleic acid, ALA = α-linolenic acid, D9D = Δ9-desaturase, D5D = Δ5-desaturase, D6D = Δ6-desaturase

[3]Percentage of *n*-6 PUFA in fatty acids with three or more double bonds

[4]Percentage of *n*-3 PUFA in fatty acids with three or more double bonds

Table S3: Subgroup Analysis of relevant fatty acids in the intervention groups

Fatty acid	Subgroup	*n*	Change from baseline after 16 weeks (%)	*p*-Value
ARA	**m**	12	-0.24 (-5.76, 6.28)	n.s
	**w**	40	-2.77 (-7.92, 3.45)	
	**BMI** (< 31)	25	-3.74 (-5.51, 2.09)	n.s
	**BMI** (≥ 31)	23	-1.48 (-8.86, 3.56)	
	**Age** (< 41)	26	-2.77 (-8.52, 4.44)	n.s
	**Age** (≥ 41)	26	-1.90 (-4.53, 4.13)	
	**LA** (< 10.7 %FAME)	26	-2.02 (-5.03, 4.47)	n.s
	**LA** (≥ 10.7 % FAME)	26	-2.65 (-8.54, 4.10)	
	**ARA** (< 15.1 %FAME)	26	-0.74 (-4.53, 7.65)	n.s
	**ARA** (≥ 15.1 %FAME)	26	-3.69 (-8.52, 2.30)	
	**EPA** (< 0.70 % FAME)	26	-1.80 (-9.07, 4.10)	n.s
	**EPA** (≥ 0.70 %FAME)	26	-2.65 (-6.40, 4.47)	
C22:4*n*-6	**m**	12	-6.28 (-10.56, 0.68)	n.s
	**w**	40	-6.39 (-11.11, 2.25)	
	**BMI** (< 31)	25	-7.38 (-12.90, -2.64)	n.s
	**BMI** (≥ 31)	23	-3.29 (-9.74, 2.45)	
	**Age** (< 41)	26	-6.46 (-11.39, 0.29)	n.s
	**Age** (≥ 41)	26	-6.39 (-10.85, 2.73)	
	**LA** (< 10.7 %FAME)	26	-4.33 (-10.85, 1.70)	n.s
	**LA** (≥ 10.7 % FAME)	26	-6.97 (-11.29, 1.96)	
	**ARA** (< 15.1 %FAME)	26	-6.40 (-11.29, 2.92)	n.s
	**ARA** (≥ 15.1 %FAME)	26	-6.37 (-10.17, 0.29)	
	**EPA** (< 0.70 % FAME)	26	-7.10 (-12.90, 1.96)	n.s
	**EPA** (≥ 0.70 %FAME)	26	-5.59 (-10.13, 1.70)	
C22:5*n*-6	**m**	12	-8.45 (-13.36, 2.50)	n.s
	**w**	39	-14.31 (-25.28, -0.90)	
	**BMI** (< 31)	25	-14.64 (-26.66, -2.75)	n.s
	**BMI** (≥ 31)	23	-9.98 (-17.82, 1.23)	
	**Age** (< 41)	26	-9.61 (-18.73, 0.05)	n.s
	**Age** (≥ 41)	25	-11.83 (24.25, 0.70)	
	**LA** (< 10.7 %FAME)	26	-13.07 (-20.18, -2.14)	n.s
	**LA** (≥ 10.7 % FAME)	25	-7.47 (-20.23, 1.24)	
	**ARA** (< 15.1 %FAME)	25	-9.16 (-23.64, 2.00)	n.s
	**ARA** (≥ 15.1 %FAME)	26	-11.86 (-18.17, -0.76)	
	**EPA** (< 0.70 % FAME)	26	-8.04 (-26.35, 1.67)	n.s
	**EPA** (≥ 0.70 %FAME)	25	-14.31 (-18.15, -4.35)	
EPA	**m**	12	3.61 (-9.34, 10.56)	n.s
	**w**	40	5.17 (-15.46, 40.14)	
	**BMI** (< 31)	25	16.78 (-2.92, 63.76)	0.004
	**BMI** (≥ 31)	23	-2.09 (-20.17, 12.11)	
	**Age** (< 41)	26	4.39 (-6.83, 34.47)	n.s
	**Age** (≥ 41)	26	1.76 (-18.16, 29.44)	
	**LA** (< 10.7 %FAME)	26	3.68 (-18.16, 31.38)	n.s
	**LA** (≥ 10.7 % FAME)	26	5.42 (-9.03, 28.08)	
	**ARA** (< 15.1 %FAME)	26	3.59 (-16.45, 29.92)	n.s
	**ARA** (≥ 15.1 %FAME)	26	4.39 (-11.27, 31.38)	
	**EPA** (< 0.70 % FAME)	26	11.85 (-6.35, 66.2)6	0.020
	**EPA** (≥ 0.70 %FAME)	26	1.29 (-18.16, 18.66)	

DPA	**m**	12	5.15 (-3.09, 9.44)	n.s
	**w**	40	1.32 (-5.15, 11.6)	
	**BMI** (< 31)	25	7.94 (1.09, 16.48)	0.001
	**BMI** (≥ 31)	23	-1.58 (-7.10, 2.49)	
	**Age** (< 41)	26	2.77 (-5.28, 12.67)	n.s
	**Age** (≥ 41)	26	1.99 (-4.94, 7.96)	
	**LA** (< 10.7 %FAME)	26	-0.17 (-5.46, 12.83)	n.s
	**LA** (≥ 10.7 % FAME)	26	3.72 (-1.45, 9.16)	
	**ARA** (< 15.1 %FAME)	26	3.60 (-2.49, 12.30)	n.s
	**ARA** (≥ 15.1 %FAME)	26	-0.70 (-5.05, 6.18)	
	**EPA** (< 0.70 % FAME)	26	4.19 (-5.39, 12.13)	n.s
	**EPA** (≥ 0.70 %FAME)	26	0.78 (-4.83, 8.47)	
DHA	**m**	12	1.92 (-1.44, 16.04)	n.s
	**w**	40	2.52 (-4.89, 7.07)	
	**BMI** (< 31)	25	4.44 (-1.18, 15.55)	0.018
	**BMI** (≥ 31)	23	-1.92 (-5.37, 3.83)	
	**Age** (< 41)	26	1.89 (-3.61, 10.57)	n.s
	**Age** (≥ 41)	26	2.52 (-4.88, 8.30)	
	**LA** (< 10.7 %FAME)	26	-0.17 (-5.46, 12.83)	n.s
	**LA** (≥ 10.7 % FAME)	26	3.72 (-1.45, 9.16)	
	**ARA** (< 15.1 %FAME)	26	3.60 (-2.49, 12.30)	n.s
	**ARA** (≥ 15.1 %FAME)	26	-0.70 (-5.05, 6.18)	
	**EPA** (< 0.70 % FAME)	26	6.43 (-1.44, 18.04)	0.006
	**EPA** (≥ 0.70 %FAME)	26	-1.51 (-5.46, 3.87)	
LC *n*-3 PUFA	**m**	12	3.73 (-2.21, 14.27)	n.s
	**w**	40	1.35 (-4.51, 12.03)	
	**BMI** (< 31)	25	5.49 (-2.84, 17.88)	0.001
	**BMI** (≥ 31)	23	-0.57 (-5.17, 3.92)	
	**Age** (< 41)	26	3.73 (-4.50, 13.07)	n.s
	**Age** (≥ 41)	26	1.35 (-4.32, 12.51)	
	**LA** (< 10.7 %FAME)	26	-3.10 (-5.35, 15.25)	n.s
	**LA** (≥ 10.7 % FAME)	26	3.76 (0.09, 10.78)	
	**ARA** (< 15.1 %FAME)	26	3.76 (-2.81, 13.71)	n.s
	**ARA** (≥ 15.1 %FAME)	26	0.75 (-5.35, 11.65)	
	**EPA** (< 0.70 % FAME)	26	7.73 (-3.27, 17.88)	0.022
	**EPA** (≥ 0.70 %FAME)	26	0.46 (-5.06, 4.42)	

Variable expressed as median (25th, 75th percentile); Abbreviations: m = men, w = women BMI = Body mass index, PUFA = polyunsaturated fatty acids, EPA = eicosapentaenoic acid, LA = linoleic acid, ARA = arachidonic acid, DPA =docosapentaenoic acid, DHA = docosahexaenoic acid LC = long-chain, *n* = omega

Table S4: Micronutrient status during the intervention

		**Group 1** (*n*=18)	T[1]	G[2]	**Group 2** (*n*=19)	T	G	**Group 3** (*n*=15)	T	G	**Control** (*n*=16)	T	G
						**Vitamins**							
Folic acid [ng/ml]	0	7.76 (± 2.32)<br>7.30 (6.20, 9.20)	a,b	a	6.75 (± 2.59)<br>5.90 (4.60, 9.10)	a	a	7.17 (± 2.70)<br>7.80 (4.90, 9.20)	a	a	7.73 (± 3.72)<br>6.75 (5.13, 9.70)	a	a
	4	8.67 (± 2.64)<br>8.70 (6.70, 11.30)	a	a	8.17 (± 2.50)<br>8.20 (6.50, 8.80)	b	a	7.74 (± 2.89)<br>7.40 (6.25, 10.45)	a	a	8.94 (± 3.78)<br>8.70 (5.65, 11.50)	a	a
	8	7.67 (± 2.67)<br>7.60 (5.60, 9.60)	a,b	a	6.81 (± 2.36)<br>6.40 (5.70, 7.30)	a	a	7.07 (± 3.22)<br>7.90 (4.45, 9.65)	a	a	7.78 (± 3.36)<br>6.60 (6.05, 10.18)	a	a
	12	7.35 (± 2.40)<br>7.50 (5.60, 8.20)	a,b	a	6.46 (± 2.21)<br>6.20 (5.10, 7.90)	a	a	7.89 (± 3.27)<br>7.30 (5.10, 10.80)	a	a	7.89 (± 4.96)<br>6.20 (5.28, 8.65)	a	a
	16	7.18 (± 2.75)<br>6.70 (5.30, 7.40)	b	a	6.36 (± 2.14)<br>5.70 (5.30, 7.40)	a	a	7.35 (± 3.44)<br>6.70 (5.45, 8.75)	a	a	7.54 (± 3.42)<br>6.60 (5.28, 9.55)	a	a
	$\%_{0\rightarrow12}$	-2.24 (± 27.27)<br>-10.35 (-23.80, 22.91)		a	1.07 (± 31.33)<br>-6.56 (-27.05, 18.03)		a	11.44 (± 22.21)<br>9.26 (-4.00, 29.49)		a	1.37 (± 28.28)<br>-1.98 (-17.40, 23.31)		a
	$\%_{0\rightarrow16}$	-6.74 (± 27.32)<br>-4.84 (-30.04, 10.52)	a	a	-3.23 (± 24.40)<br>-8.01 (-18.86, 15.34)		a	4.52 (± 30.14)<br>-2.91 (-22.48, 30.95)		a	2.07 (± 38.64)<br>-2.11 (-35.85, 20.47)		a
Vitamin B_{12} [pmol/l]	0	353.94 (± 113.55)<br>313.00 (288.00, 383.00)	a,b	a	293.24 (± 66.16)<br>307.00 (257.00, 321.00)	a	a	310.07 (± 88.74)<br>303.00 (262.00, 370.00)	a,b	a	279.79 (± 107.32)<br>262.50 (194.50, 341.00)	a	a
	4	363.71 (± 109.29)<br>369.00 (286.00, 414.00)	b	a	317.53 (± 65.13)<br>326.00 (286.00, 348.00)	b	a	327.87 (± 96.60)<br>338.00 (285.00, 381.00)	b	a	280.43 (± 99.44)<br>251.00 (192.75, 347.25)	a	a
	8	321.47 (± 96.35)<br>326.00 (255.00, 375.00)	c	a	265.29 (± 58.36)<br>252.00 (232.00, 308.00)	c	a	297.93 (± 73.39)<br>295.00 (272.50, 343.00)	a	a	276.86 (± 135.83)<br>219.00 (181.00, 317.00)	a	a
	12	341.12 (± 120.57)<br>305.00 (251.00, 387.00)	a,c	a	263.29 (± 63.89)<br>282.00 (225.00, 308.00)	c	a	295.13 (± 81.08)<br>287.00 (271.00, 357.00)	a	a	260.14 (± 96.87)<br>243.00 (171.25, 326.00)	a	a
	16	317.35 (± 82.73)<br>284.00 (250.00, 381.00)	a,c	a	266.71 (± 58.90)<br>261.00 (219.00, 309.00)	c	a	486.33 (± 665.13)<br>292.00 (277.00, 384.50)	a,b	a	258.07 (± 88.01)<br>227.50 (188.00, 319.75)	a	a
	$\%_{0\rightarrow12}$	-2.77 (± 19.40)<br>-4.36 (-15.96, 9.64)		a	-11.31 (± 10.41)<br>-12.15 (-19.05, -3.91)		a	-4.14 (± 10.82)<br>-5.88 (-10.55, -0.57)		a	-6.23 (± 16.65)<br>-1.83 (-21.40, 0.16)		a
	$\%_{0\rightarrow16}$	-7.50 (± 18.73)<br>-6.90 (-13.93, -1.52)		a	-9.53 (± 8.28)<br>-9.43 (-16.00, -2.37)		a	79.72 (± 304.46)<br>-1.57 (-6.83, 2.45)		a	-6.04 (± 15.07)<br>-11.33 (-19.04, 6.67)		a
Holo-Transcobalamin [pmol/l]	0	83.54 (± 35.19)<br>76.90 (63.10, 93.10)	a	a	80.38 (± 23.73)<br>80.50 (60.00, 102.00)	a,b	a	80.96 (± 23.11)<br>68.60 (61.60, 91.35)	a	a	80.78 (± 38.90)<br>73.40 (62.60, 88.10)	a	a
	4	92.79 (± 43.13)<br>81.30 (69.90, 113.00)	a	a	83.21 (± 26.95)<br>88.10 (62.20, 101.00)	b	a	75.67 (± 19.70)<br>79.60 (63.90, 89.85)	a	a	75.45 (± 29.62)<br>75.20 (59.50, 87.50)	a	a
	8	90.21 (± 40.70)<br>83.50 (70.00, 96.00)	a	a	76.27 (± 29.86)<br>78.00 (49.40, 91.60)	a,b	a	71.67 (± 18.90)<br>70.60 (64.90, 77.50)	a	a	78.41 (± 53.91)<br>68.20 (49.40, 78.70)	a	a
	12	97.57 (± 51.70)<br>90.00 (60.20, 104.00)	a	a	71.10 (± 22.28)<br>73.50 (60.80, 79.60)	a	a	72.98 (± 22.22)<br>76.80 (57.00, 90.10)	a	a	72.74 (± 36.49)<br>64.40 (56.60, 74.60)	a	a
	16	81.29 (± 30.24)<br>81.10 (60.00, 92.30)	a	a	72.19 (± 24.81)<br>76.00 (52.30, 89.00)	a	a	89.55 (± 60.80)<br>75.70 (60.45, 89.15)	a	a	69.69 (± 24.63)<br>70.40 (57.50, 78.90)	a	a

	$\%^{0\rightarrow 12}$	28.10 (± 92.46)<br>2.02 (-10.58, 14.38)		a	-8.83 (± 24.05)<br>-11.35 (-24.39, -1.79)		a	-9.28 (± 19.39)<br>-8.63 (-18.32, 1.89)		a	-9.21 (± 17.02)<br>-11.84 (-23.40, 1.24)		a
	$\%^{0\rightarrow 16}$	4.68 (± 45.77)<br>-10.64 (-16.12, 3.92)		a	-7.22 (± 22.58)<br>-7.65 (-23.25, -1.38)		a	19.18 (± 108.91)<br>-9.03 (-14.04, -3.50)		a	-9.90 (± 20.31)<br>-10.44 (-30.39, 5.45)		a
Vitamin D_3<br>[nmol/l]	0	55.56 (± 21.02)<br>50.70 (43.50, 66.60)	a	a,b	62.64 (± 39.60)<br>49.00 (44.70, 70.20)	a	a	42.98 (± 12.80)<br>38.80 (33.15, 51.95)	a	b	53.31 (± 39.93)<br>43.55 (32.77, 48.70)	a	a,b
	4	58.66 (± 23.64)<br>57.30 (47.80, 65.80)	a,b	a	67.46 (± 35.99)<br>55.50 (51.10, 72.50)	a	a	46.99 (± 12.74)<br>46.30 (40.10, 52.90)	b	a	52.26 (± 31.07)<br>43.80 (38.60, 55.80)	a	a
	8	63.35 (± 20.69)<br>61.00 (53.00, 80.60)	b	a	67.65 (± 25.22)<br>62.20 (55.00, 73.60)	a,c	a	49.43 (± 14.63) b<br>46.40 (40.90, 62.40)	b,d	a	57.37 (± 29.71)<br>50.65 (43.10, 59.40)	a	a
	12	74.09 (± 20.80)<br>69.30 (59.60, 91.80)	c	a,b	81.98 (± 20.00)<br>80.00 (74.80, 85.60)	b	b	60.53 (± 13.33)<br>65.00 (51.10, 67.55)	c	a	67.36 (± 30.52)<br>62.60 (51.88, 70.43)	b	a
	16	69.75 (± 17.74)<br>68.60 (57.60, 87.80)	d	a	72.64 (± 14.28)<br>69.40 (63.30, 81.60)	b,c	a	54.01 (± 13.13)<br>52.70 (46.20, 62.50)	d	b	69.49 (± 29.05)<br>64.25 (49.20, 78.65)	b	a,b
	$\%^{0\rightarrow 12}$	39.07 (± 24.04)<br>39.30 (30.49, 48.00)		a	45.03 (± 34.93)<br>54.45 (22.56, 66.22)		a	45.77 (± 27.77)<br>36.31 (22.99, 61.76)		a	43.89 (± 33.68)<br>47.26 (20.61, 58.42)		a
	$\%^{0\rightarrow 16}$	33.97 (± 27.74)<br>33.99 (21.63, 49.48)		a	31.62 (± 32.31)<br>39.32 (12.10, 50.88)		a	30.44 (± 30.72)<br>25.46 (6.83, 45.86)		a	48.54 (± 43.90)<br>37.96 (20.10, 72.95)		a
**Minerals and trace elements**													
fT3<br>[pmol/l]	0	4.65 (± 0.51)<br>4.56 (4.32, 5.04)	a	a	4.83 (± 0.60)<br>4.87 (4.30, 5.29)	a	a	4.98 (± 0.60)<br>5.06 (4.54, 5.30)	a	a	4.83 (± 0.39)<br>4.95 (4.46, 5.09)	a	a
	4	4.54 (± 0.48)<br>4.40 (4.31, 4.92)	a	a	4.75 (± 0.52)<br>4.62 (4.56, 4.94)	a	a	4.70 (± 0.43)<br>4.55 (4.32, 5.03)	a,c	a	4.78 (± 0.43)<br>4.86 (4.44, 5.16)	a	a
	8	4.62 (± 0.53)<br>4.53 (4.26, 4.97)	a	a	7.53 (± 10.95)<br>4.88 (4.64, 5.08)	a	a	4.69 (± 0.58)<br>4.49 (4.24, 5.08)	a,c	a	4.93 (± 0.49)<br>5.08 (4.70, 5.23)	a	a
	12	4.13 (± 0.56)<br>4.20 (3.99, 4.33)	b	a	4.28 (± 0.44)<br>4.36 (3.95, 4.56)	b	a	4.02 (± 0.41)<br>3.85 (3.72, 4.26)	b	a	4.29 (± 0.63)<br>4.27 (3.84, 4.73)	b	a
	16	4.19 (± 0.73)<br>4.38 (3.58, 4.71)	b	a	4.37 (± 0.45)<br>4.35 (3.99, 4.59)	b	a	4.34 (± 0.47)<br>4.55 (3.94, 4.64)	b,c	a	4.15 (± 0.75)<br>4.10 (3.48, 4.83)	b	a
	$\%^{0\rightarrow 12}$	-11.51 (± 8.80)<br>-11.12 (-17.72, -5.21)		a	-11.09 (± 6.41)<br>-12.57 (-14.17, -6.65)		a	-18.74 (± 7.16)<br>-18.34 (-23.47, -13.25)		b	-11.45 (± 8.97)<br>-14.36 (-16.33, -7.25)		b
	$\%^{0\rightarrow 16}$	-10.39 (± 11.36)<br>-12.67 (-16.08, -3.73)		a	-8.10 (± 9.20)<br>-8.62 (-13.28, -5.13)		a	-12.37 (± 8.87)<br>-11.20 (-18.20, -7.32)		a	-14.26 (± 12.10)<br>-11.85 (-20.64, -6.50)		a
fT4<br>[pmol/l]	0	16.56 (± 2.67)<br>16.00 (14.80, 18.60)	a	a	17.57 (± 2.85)<br>17.00 (15.80, 19.70)	a,b	a	16.63 (± 2.29)<br>17.30 (15.50, 17.80)	a	a	16.92 (± 2.58)<br>16.15 (15.23, 17.45)	a	a
	4	17.12 (± 3.17)<br>16.20 (15.80, 18.50)	a	a	18.49 (± 3.12)<br>17.60 (16.40, 21.50)	a	a	17.37 (± 2.45)<br>17.50 (16.35, 18.85)	a	a	16.83 (± 1.71)<br>16.85 (15.23, 18.15)	a	a
	8	15.13 (± 2.68)<br>14.80 (13.50, 15.80)	b	a	17.10 (± 4.26)<br>15.90 (14.30, 18.40)	b,c	a	14.74 (± 2.39)<br>14.80 (13.65, 16.20)	b	a	15.00 (± 2.30)<br>14.40 (13.45, 15.65)	b	a
	12	15.35 (± 3.16)<br>15.40 (13.40, 16.80)	b	a	16.05 (± 3.26)<br>15.30 (13.90, 16.80)	c,d	a	14.66 (± 2.12)<br>15.80 (13.30, 16.35)	b	a	14.47 (± 2.07)<br>14.80 (12.83, 15.88)	b,c	a
	16	15.05 (± 3.04)<br>14.70 (13.10, 16.20)	b	a	15.89 (± 2.99)<br>15.30 (14.10, 17.30)	d	a	14.81 (± 2.33)<br>14.70 (13.60, 16.05)	b	a	14.29 (± 1.96)<br>14.05 (13.05, 14.58)	c	a
	$\%^{0\rightarrow 12}$	-7.42 (± 7.09)<br>-7.93 (-12.64, -0.79)		a	-8.36 (± 11.40)<br>-10.17 (-12.98, -4.95)		a	-11.79 (± 5.46)<br>-11.81 (-14.59, -8.26)		a	-13.79 (± 9.45)<br>-14.70 (-22.17, -6.25)		a

	$\%^{0\rightarrow16}$	-9.11 (± 7.16)<br>-9.87 (-12.90, -5.79)		a	-8.03 (± 11.08)<br>-9.83 (-13.97, -3.67)		a	-10.72 (± 8.64)<br>-12.15 (-16.34, -6.51)		a	-14.33 (± 7.14)<br>-11.93 (-20.06, -9.33)		a
TSH [mU/l]	0	2.23 (± 0.76)<br>2.10 (1.75, 2.79)	a	a	1.85 (± 0.70)<br>1.81 (1.46, 2.18)	a	a	4.42 (± 7.53)<br>2.45 (1.55, 3.43)	a	a	2.69 (± 1.50)<br>2.30 (1.59, 3.55)	a	a
	4	1.94 (± 0.73)<br>1.84 (1.53, 2.65)	a	a	1.79 (± 1.01)<br>1.59 (1.15, 2.49)	a	a	4.19 (± 8.22)<br>2.28 (1.39, 2.73)	a	a	2.47 (± 1.36)<br>2.00 (1.74, 2.91)	a	a
	8	1.99 (± 0.80)<br>2.03 (1.37, 2.62)	a	a	2.02 (± 0.97)<br>2.12 (1.56, 2.37)	a	a	3.81 (± 6.75)<br>2.06 (1.27, 2.95)	a	a	2.44 (± 1.22)<br>2.32 (1.48, 2.89)	a	a
	12	1.93 (± 0.76)<br>1.77 (1.28, 2.50)	a	a	1.80 (± 0.80)<br>1.91 (1.09, 2.16)	a	a	4.71 (± 9.73)<br>2.27 (1.43, 2.79)	a	a	2.64 (± 0.93)<br>2.62 (2.27, 3.47)	a	a
	16	1.81 (± 0.70)<br>1.76 (1.47, 2.05)	a	a	1.92 (± 0.98)<br>1.90 (1.26, 2.26)	a	a	3.92 (± 6.31)<br>2.26 (1.47, 3.01)	a	a	2.37 (± 1.04)<br>2.56 (1.43, 2.69)	a	a
	$\%^{0\rightarrow12}$	-11.59 (± 28.09)<br>-18.46 (-27.64, -3.81)		a	5.13 (± 41.93)<br>0.59 (-14.12, 14.20)		a	-7.78 (± 32.19)<br>-11.85 (-34.63, 10.55)		a	19.29 (± 65.38)<br>1.10 (-24.38, 41.81)		a
	$\%^{0\rightarrow16}$	-12.70 (± 33.07)<br>-17.24 (-26.41, -4.51)		a	7.04 (± 41.77)<br>4.42 (-5.68, 15.75)		a	-2.66 (± 29.48)<br>-5.28 (-26.14, 18.44)		a	8.74 (± 73.30)<br>-15.15 (-32.75, 29.22)		a
Ferritin [µg/l]	0	179.85 (± 315.61)<br>90.30 (39.80, 164.20)	a	a	127.85 (± 108.57)<br>81.40 (49.50, 222.30)	a	a	97.19 (± 66.68)<br>104.80 (38.85, 120.05)	a	a	123.99 (± 192.16)<br>52.75 (30.18, 108.55)	a	a
	4	159.95 (± 253.96)<br>121.00 (36.60, 162.10)	a	b	123.78 (± 108.83)<br>72.30 (39.30, 194.00)	a	a	78.68 (± 58.61)<br>67.80 (31.40, 98.60)	b	a	108.84 (± 195.36)<br>44.65 (19.13, 70.75)	b, e	a
	8	143.42 (± 214.38)<br>90.40 (34.80, 148.50)	a,b	a	120.92 (± 110.81)<br>80.30 (33.50, 204.40)	a,b	a	80.07 (± 74.44)<br>73.70 (21.45, 100.95)	b	a	103.22 (± 178.07)<br>39.05 (19.75, 86.73)	c, e	a
	12	149.20 (± 260.13)<br>69.50 (36.00, 150.00)	b,c	a	106.67 (± 104.89)<br>59.30 (26.20, 175.90)	b,c	a	66.97 (± 56.17)<br>66.70 (21.10, 87.75)	c	a	96.31 (± 183.28)<br>28.70 (15.38, 68.45)	d, e	a
	16	127.99 (± 223.34)<br>83.20 (25.10, 123.10)	c	a	97.01 (± 102.29)<br>52.50 (22.10, 136.50)	c	a	62.57 (± 48.23)<br>45.20 (24.45, 84.70)	c	b	97.79 (± 192.37)<br>26.75 (11.48, 59.50)	e	a
	$\%^{0\rightarrow12}$	-16.48 (± 27.88)<br>-20.84 (-25.88, -9.24)		a	-29.18 (± 26.08)<br>-27.38 (-51.54, -13.92)		a	-36.20 (± 14.62)<br>-40.22 (-43.01, -24.58)		a	-38.68 (± 19.31)<br>-36.07 (-52.78, -29.07)		a
	$\%^{0\rightarrow16}$	-30.25 (± 22.20)<br>-26.06 (-39.97, -21.82)		a	-35.53 (± 25.75)<br>-38.60 (-47.13, -27.93)		a	-34.98 (± 16.72)<br>-35.64 (-50.40, -18.28)		a	-37.87 (± 24.80)<br>-37.40 (-50.10, -21.56)		a
Transferrin [g/l]	0	2.70 (± 0.48)<br>2.60 (2.50, 2.80)	a,c	a	2.64 (± 0.38)<br>2.60 (2.30, 2.80)	a	a	2.66 (± 0.41)<br>2.70 (2.40, 2.85)	a,b	a	2.99 (± 0.62)<br>2.90 (2.50, 3.48)	a	a
	4	2.58 (± 0.39)<br>2.50 (2.40, 2.60)	b		2.56 (± 0.38)<br>2.50 (2.30, 2.90)	a	a	2.56 (± 0.40)<br>2.60 (2.30, 2.75)	a	a	2.87 (± 0.57)<br>2.70 (2.40, 3.23)	a	a
	8	2.58 (± 0.43)<br>2.50 (2.40, 2.70)	a,b	a	2.46 (± 0.31)<br>2.40 (2.20, 2.70)	b	a	2.55 (± 0.40)<br>2.50 (2.30, 2.85)	a,b	a	2.86 (± 0.60)<br>2.85 (2.30, 3.23)	a	a
	12	2.66 (± 0.45)<br>2.60 (2.30, 2.80)	a,b,c	a	2.61 (± 0.38)<br>2.60 (2.30, 2.90)	a	a	2.55 (± 0.34)<br>2.70 (2.35, 2.80)	a,b	a	2.92 (± 0.57)<br>2.85 (2.43, 3.05)	a	a
	16	2.76 (± 0.47)<br>2.60 (2.50, 2.90)	c	a	2.62 (± 0.41)<br>2.50 (2.30, 3.00)	a	a	2.69 (± 0.45)<br>2.70 (2.40, 2.95)	b	a	3.06 (± 0.67)<br>2.90 (2.45, 3.53)	a	a
	$\%^{0\rightarrow12}$	-1.32 (± 8.25)<br>-2.17 (-7.62, 3.39)		a	-0.53 (± 7.27)<br>0.00 (-5.67, 4.08)		a	-3.42 (± 8.58)<br>0.00 (-10.26, 0.00)		a	-1.19 (± 5.95) 0.00 (-5.56,<br>4.04)		a
	$\%^{0\rightarrow16}$	2.51 (± 5.99)<br>0.00 (0.00, 4.57)		a	0.73 (± 7.89)<br>0.00 (-3.92, 3.77)		a	1.28 (± 7.71)<br>2.86 (-3.94, 4.58)		a	1.66 (± 7.62)<br>0.00 (-4.04, 4.09)		a

Potassium [mmol/l]	0	3.91 (± 0.19)<br>3.93 (3.79, 4.01)	a	a	3.95 (± 0.27)<br>3.98 (3.80, 4.04)	a	a	3.86 (± 0.23)<br>3.94 (3.72, 4.01)	a	a	3.94 (± 0.21)<br>3.89 (3.78, 3.98)	a	a
	4	4.01 (± 0.16)<br>4.02 (3.86, 4.10)	a	a	3.97 (± 0.23)<br>3.99 (3.80, 4.12)	a	a	3.88 (± 0.22)<br>3.95 (3.78, 4.04)	a	a	3.99 (± 0.14)<br>4.05 (3.90, 4.09)	a	a
	8	3.96 (± 0.16)<br>3.96 (3.84, 4.07)	a	a	3.95 (± 0.18)<br>4.01 (3.81, 4.03)	a	a	3.99 (± 0.26)<br>4.05 (3.74, 4.19)	a	a	4.02 (± 0.20)<br>4.05 (3.87, 4.13)	a	a
	12	3.97 (± 0.21)<br>3.97 (3.82, 4.08)	a	a	3.96 (± 0.14)<br>3.94 (3.90, 3.96)	a	a	3.94 (± 0.19)<br>3.97 (3.92, 4.04)	a	a	4.02 (± 0.18)<br>3.99 (3.92, 4.09)	a	a
	16	3.96 (± 0.23)<br>3.93 (3.83, 4.05)	a	a	3.95 (± 0.21)<br>3.98 (3.78, 4.08)	a	a	3.90 (± 0.22)<br>3.89 (3.81, 4.05)	a	a	4.01 (± 0.28)<br>3.92 (3.79, 4.22)	a	a
	$\%^{0\rightarrow12}$	1.58 (± 4.72)<br>1.46 (-0.20, 4.79)		a	1.19 (± 6.52)<br>1.56 (-1.30, 4.89)		a	2.29 (± 3.92)<br>1.27 (0.00, 5.82)		a	1.53 (± 4.51)<br>2.66 (0.71, 2.86)		a
	$\%^{0\rightarrow16}$	1.23 (± 5.71)<br>0.51 (-3.83, 6.55)		a	1.32 (± 5.83)<br>0.25 (-1.39, 4.37)		a	1.27 (± 6.33)<br>1.78 (-2.25, 3.87)		a	1.47 (± 6.80)<br>0.14 (-2.84, 2.40)		a
Calcium [mmol/l]	0	2.29 (± 0.07)<br>2.30 (2.24, 2.33)	a	a	2.29 (± 0.09)<br>2.27 (2.25, 2.33)		a	2.33 (± 0.09)<br>2.33 (2.27, 2.36)	a	a	2.28 (± 0.08)<br>2.26 (2.23, 2.32)	a	a
	4	2.27 (± 0.06)<br>2.25 (2.24, 2.29)	a	a	2.23 (± 0.06)<br>2.22 (2.19, 2.25)	b	a	2.25 (± 0.07)<br>2.24 (2.20, 2.30)	b	a	2.25 (± 0.09)<br>2.27 (2.18, 2.33)	a	a
	8	2.28 (± 0.06)<br>2.27 (2.24, 2.32)	a	a	2.26 (± 0.07)<br>2.24 (2.20, 2.31)	a,b	a	2.27 (± 0.09)<br>2.28 (2.21, 2.33)	a,b	a	2.26 (± 0.07)<br>2.24 (2.21, 2.32)	a	a
	12	2.27 (± 0.06)<br>2.26 (2.23, 2.30)	a	a	2.24 (± 0.06)<br>2.24 (2.18, 2.27)	b	a	2.24 (± 0.06)<br>2.27 (2.21, 2.29)	b	a	2.22 (± 0.08)<br>2.22 (2.20, 2.28)	a	a
	16	2.28 (± 0.05)<br>2.28 (2.27, 2.32)	a	a	2.26 (± 0.05)<br>2.27 (2.22, 2.28)	a,b	a	2.28 (± 0.06)<br>2.29 (2.23, 2.33)	a,b	a	2.29 (± 0.06)<br>2.29 (2.25, 2.31)	a	a
	$\%^{0\rightarrow12}$	-1.14 (± 3.10)<br>-1.73 (-3.86, 0.92)		a	-2.16 (± 2.77)<br>-1.82 (-4.11, -0.67)		a	-3.85 (± 3.31)<br>-2.99 (-7.38, -1.57)		a	-2.63 (± 2.62)<br>-2.19 (-4.29, -0.89)		a
	$\%^{0\rightarrow16}$	-0.39 (± 3.67)<br>-0.18 (-3.08, 1.77)		a	-1.16 (± 2.47)<br>-0.88 (-2.59, 0.00)		a	-2.32 (± 3.76)<br>-1.33 (-4.74, 0.86)		a	0.01 (± 2.27)<br>-0.43 (-1.79, 1.12)		a
Magnesium 24h urin [mmol/24h]	0	4.49 (± 1.41)<br>4.10 (3.50, 5.20)	a	a	4.67 (± 1.34)<br>4.60 (3.70, 5.10)	a	a	4.91 (± 1.58)<br>4.80 (4.00, 4.95)	a	a	4.16 (± 1.39)<br>3.70 (3.30, 5.00)	a	a
	16	4.04 (± 1.59)<br>3.80 (2.90, 4.70)	a	a	4.22 (± 1.34)<br>4.00 (3.40, 4.90)	a	a	4.49 (± 1.33)<br>4.40 (3.50, 5.30)	a	a	4.60 (± 0.94)<br>4.40 (3.70, 5.20)	a	a
	$\%^{0\rightarrow16}$	-2.49 (± 52.84)<br>-14.67 (-25.48, 12.86)		b	-8.24 (± 18.77)<br>-7.84 (-21.43, 7.14)		a	-2.76 (± 28.90)<br>0.00 (-14.35, 16.33)		a	13.64 (± 28.52)<br>8.00 (-4.33, 34.06)		a
Sodium 24h urin [mmol/24h]	0	174.44 (± 84.84)<br>145.00 (128.50, 204.25)	a	a	172.88 (± 73.40)<br>148.00 (122.00, 185.00)	a	a	180.33 (± 66.76)<br>173.00 (140.50, 202.00)	a	a	155.2 (± 50.9)<br>143.0 (114.0, 192.5)	a	a
	16	140.56 (± 66.29)<br>128.00 (104.50, 143.50)	b	a	173.47 (± 72.17)<br>165.00 (112.00, 206.00)	a	a,b	148.33 (± 47.06)<br>146.00 (117.50, 163.50)	a	a,b	192.3 (± 60.0)<br>202.0 (150.0, 233.5)	b	b
	$\%^{0\rightarrow16}$	-14.23 (± 34.20)<br>-15.26 (-38.47, 0.00)			5.57 (± 36.45)<br>-8.33 (-21.82, 34.77)		a,b	-7.90 (± 39.09)<br>-4.51 (-42.11, 16.10)		a	28.3 (± 36.6)<br>23.73 (7.09, 48.73		b

Variable expressed as mean (±SD) and as median (25th, 75th percentile); [1]comparison of time points within the respective diet group (T; column by column); [2]comparison between diet groups at individual time points (G; line by line); time points or groups without a common letter are significantly different, $p < 0.05$. Abbreviations: fT3 = free tri-iodothyronine, fT4 = free thyroxine, TSH = thyroid stimulating hormone
